# Gene bionetworks that regulate ovarian primordial follicle assembly

**DOI:** 10.1186/1471-2164-14-496

**Published:** 2013-07-23

**Authors:** Eric Nilsson, Bin Zhang, Michael K Skinner

**Affiliations:** 1 Center for Reproductive Biology, School of Biological Sciences, Washington State University, Pullman, WA 99164-4236, USA; 2 Department of Genetics & Genomic Sciences, Mount Sinai School of Medicine, New York, NY, USA

**Keywords:** Ovary, Primordial follicle, Assembly, Ovarian development, PCO, POI, Transcriptome, Female fertility, Genomics, System biology

## Abstract

**Background:**

Primordial follicle assembly is the process by which ovarian primordial follicles are formed. During follicle assembly oocyte nests break down and a layer of pre-granulosa cells surrounds individual oocytes to form primordial follicles. The pool of primordial follicles formed is the source of oocytes for ovulation during a female’s reproductive life.

**Results:**

The current study utilized a systems approach to detect all genes that are differentially expressed in response to seven different growth factor and hormone treatments known to influence (increase or decrease) primordial follicle assembly in a neonatal rat ovary culture system. One novel factor, basic fibroblast growth factor (FGF2), was experimentally determined to inhibit follicle assembly. The different growth factor and hormone treatments were all found to affect similar physiological pathways, but each treatment affected a unique set of differentially expressed genes (signature gene set). A gene bionetwork analysis identified gene modules of coordinately expressed interconnected genes and it was found that different gene modules appear to accomplish distinct tasks during primordial follicle assembly. Predictions of physiological pathways important to follicle assembly were validated using ovary culture experiments in which ERK1/2 (MAPK1) activity was increased.

**Conclusions:**

A number of the highly interconnected genes in these gene networks have previously been linked to primary ovarian insufficiency (POI) and polycystic ovarian disease syndrome (PCOS). Observations have identified novel factors and gene networks that regulate primordial follicle assembly. This systems biology approach has helped elucidate the molecular control of primordial follicle assembly and provided potential therapeutic targets for the treatment of ovarian disease.

## Background

Complex and interconnected networks of gene expression, cellular signaling and other processes within cells and organs are what control biological processes. This raises the concern that the common reductionist experimental approach to biomedical research may not be adequate to fully understand the systems that control these processes. Reductionist experiments will commonly impose single treatments onto the biological entity under study and measure a single response parameter compared to controls. A relevant example from the authors’ own laboratory is the study of the effect that treatment of neonatal rat ovaries with anti-Müllerian hormone (AMH) has on the proportion of primordial follicles formed [1]. Results from these types of experiments can provide clear information about candidate regulatory factors, but typically do not elucidate the network of factors or signals that are required for a normal biological process. A systems biology experimental approach to studying normal development can be a powerful tool that is complementary to the more reductionist methods. The goal of the current study is to use a systems biology approach to identify gene expression networks involved in the formation of ovarian primordial follicles (primordial follicle assembly), and to identify putative regulatory factors involved in this developmental process.

Primordial follicle assembly is the process by which ovarian primordial follicles are formed. A primordial follicle is composed of an oocyte arrested in prophase of the first meiotic division and surrounded by a single layer of pre-granulosa cells [2]. Follicle assembly in mammals occurs either during gestation (e.g. cattle and humans) or shortly after birth (e.g. rodents). The pool of assembled primordial follicles is the source of oocytes for follicle growth and ovulation over the course of a female’s reproductive life [3]. When the primordial follicle pool is depleted reproduction ceases and women enter menopause [2-7]. Prior to follicle assembly, mitotic proliferation of germ cells creates groups of cells linked by intracellular bridges and surrounded by an epithelial/mesenchymal cell layer and the structures are called oocyte nests and when the surrounding stromal cells are considered ovigerous cords [8-10]. The mitotically arrested germ cells within the nests enter meiosis and progress to the diplotene stage of prophase one of meiosis and arrest at that stage until such time as ovulation occurs [5,6,11].

During the follicle assembly process oocyte nests break down and a single layer of pre-granulosa cells surrounds individual oocytes to form primordial follicles [3]. The majority of oocytes in each nest undergo apoptosis during follicle assembly [3,6,12,13]. Abnormalities in the follicle assembly process can lead to a reduced primordial follicle pool size and reproductive capacity. Abnormal pool size may lead to some types of infertility such as Primary Ovarian Insufficiency (POI) in which the follicle pool is depleted early in life and women undergo early menopause [14,15]. Previous research has shown that several extracellular signaling molecules (e. g. growth factors and hormones) can regulate follicle assembly [3,5-7]. These studies have primarily used a reductionist approach to test candidate factors one at a time for their ability to affect the assembly process. Growth factors and hormones that have been shown to regulate primordial follicle assembly include anti-Müllerian hormone (AMH) (decrease) [1], connective tissue growth factor (CTGF) (increase) [16], estradiol (E2) (increase) [17-21], activin A (increase) [21], progesterone (P4) (decrease) [17,18,22,23], tumor necrosis factor alpha (TNFa) (decrease) [23-25], members of the notch/jagged signaling pathway (increase) [26], members of the brain derived neurotrophic factor (BDNF) / NTRK2 neurotrophin signaling pathway [27,28] and kit ligand (KITL) and growth differentiation factor-9 (GDF9) (increase) [29]. Evidence suggests fibroblast growth factor-2 (FGF2) may also be a regulator of follicle assembly [1,30], although this has not been confirmed experimentally.

A systems biology experimental approach was employed in the current study to expand upon the results of these previous experiments that examined single gene effects on the follicle assembly process. A similar systems biology approach has been used previously to identify coordinately and interconnected expressed gene modules and gene networks that regulate the primordial to primary follicle transition which is the subsequent stage of ovarian follicle development [31]. This previous study used a systems approach to elucidate the suite of genes involved in initiating the development of arrested primordial follicles to initiate folliculogenesis. In the current investigation, whole rat ovaries from zero-day old rats were cultured *in vitro* in a manner that allows primordial follicle assembly to occur. The ovaries were treated with one of several different extracellular signaling factors that have been shown to regulate follicle assembly. Messenger RNA was isolated from the ovaries and used for microarray transcriptome analysis to globally survey gene expression under these different treatment conditions. The effects of each signaling factor treatment were analyzed to determine similarities and differences in gene expression between the treatments. A gene bionetwork analysis subjected all the differentially expressed genes across all treatments to a weighted co-expression cluster analysis to identify groups of genes (i.e. modules) whose expression was regulated in a coordinated and interconnected manner [32-35]. In this type of analysis biological systems are surveyed with microarrays multiple times with and without perturbations that cause the system to change. The coordinately and interconnected expressed gene modules identified are often associated with specific physiological processes and have been used to identify potential therapeutic targets [32,36,37]. In addition, the various groups and modules of genes identified were subjected to an unbiased gene network analysis that compared gene lists to databases of known gene binding and/or functional relationships. The gene expression analyses can then be interpreted from the standpoint of physiological function and important regulatory gene networks. The objective of the investigation was to use a systems biology experimental approach to identify gene expression networks involved in regulating primordial follicle assembly. Novel regulatory factors and potential therapeutic targets were identified that correlate with normal follicle assembly and associated ovarian disease.

## Results

### Actions on primordial follicle assembly

**Figure 1:**
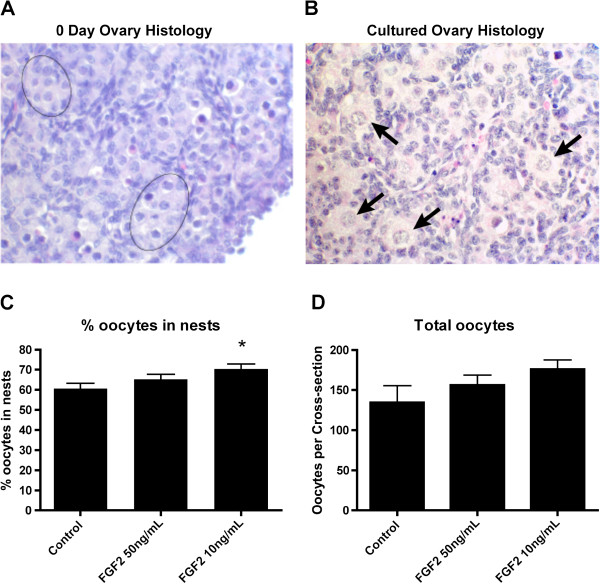
**Validation of FGF2 as a growth factor that affects primordial follicle assembly. A)** Representative image of a 0-day old rat ovary, showing oocytes in nests (circle nests). **B)** Representative image of an ovary after two days in culture, showing some oocytes assembled into primordial follicles (arrows). **C)** Proportion of oocytes still retained in nests after two days of culture with or without FGF2 treatment. **D)** Total number of oocytes per ovarian cross-section after culture. * = p<0.05 compared to untreated Control by Student’s *t*-test.

In the selection of regulatory factors to be used in the current study one novel factor was considered. Previous research [1,30] indicated that FGF2 might be a regulatory growth factor for the follicle assembly process. In order to determine if FGF2 would be included as a treatment in the current study, organ culture experiments were performed to empirically test the effects of FGF2 on follicle assembly. Ovaries from zero-day old rats containing un-assembled oocytes in nests (Figure 1A) were placed into an organ culture system and cultured for two days with or without FGF2. After culture ovaries were fixed, sectioned, stained and evaluated morphologically (Figure 1B). The number of oocytes in oocyte nests and assembled into primordial follicles was observed (see Methods). Results indicate that treatment with 10ng/ml FGF2 resulted in a modest but statistically significant increase in the proportion of oocytes retained in unassembled nests (Figure 1C). A larger duration culture of four days promotes a greater magnitude response, but is a combined effect of oocyte survival and follicle assembly, such that the shorter duration culture was used to focus on follicle assembly. A 50 ng/ml dose did not have an effect in comparison with the 10 ng/ml dose which is assumed to be due to negative feedback regulation during the 2 day culture period required. There was no statistical difference in the total number of oocytes per ovarian cross-section with FGF2 treatment (Figure 1D), although there was a trend for oocyte numbers to rise. Observations suggest FGF2 acts to inhibit the follicle assembly process. Based on these results it was decided that FGF2 be included as a treatment in the follicle assembly network experiments.

### Growth factor and hormone regulation of the ovarian transcriptome

To determine the gene networks and processes involved in follicle assembly ovaries from zero-day old rats were placed into an organ culture system and exposed to different regulatory factors. The ovaries were treated for 24 hours with one of each of the following regulatory extracellular signaling factors: AMH, CTGF, E2, FGF2, activin A, P4, TNFa, or were untreated as Controls. CTGF, activin A, estradiol have been shown the increase assembly, while AMH, progesterone and TNFa decrease assembly. A 24 hour culture period was used to minimize the impact of differences in follicle numbers (morphological impact), due to the required 2 days of culture to observe detectable morphological differences. After culture the ovaries receiving the same treatment from one culture well were pooled and RNA isolated. There were three different experiments involving different ovaries for each of the seven treatment compounds, and seven different experiments with different ovaries for the controls, for a total of 28 samples (see Methods). Gene expression in the RNA samples was evaluated using Affymetrix© Rat Gene 1.0 ST microarrays. Array data pre-processing and evaluation determined that one array (P4-treated) was abnormal and an outlier, so that array was eliminated from further analysis (Additional file 1: Figure S1).

**Figure 2:**
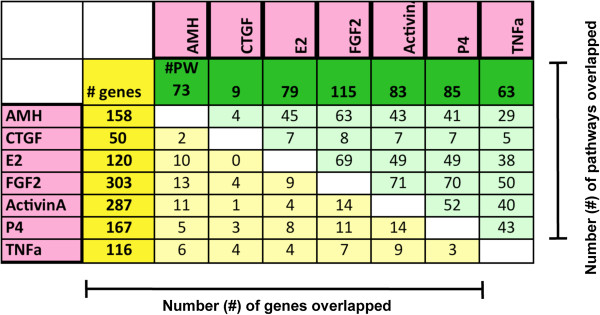
**Number of genes and physiological pathways overlapped between treatment group (signature) lists.** Total number of differentially expressed genes for each treatment is shown in dark yellow column, while numbers of genes in common between each pair of treatment groups are in light yellow columns. Total number of KEGG pathways affected by each treatment is shown in dark green row; numbers of KEGG pathways in common for each pair of treatment groups are shown in light green rows. A KEGG pathway is considered affected if ≥ 3 differentially expressed genes are present in the pathway.

**Figure 3:**
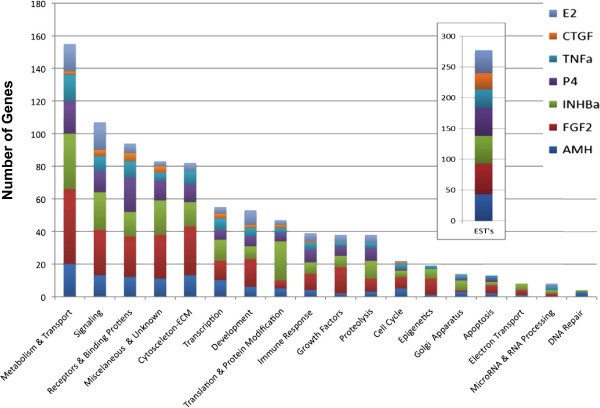
**Numbers of genes with mRNA expression levels significantly different between Control and treated ovaries after two days of culture.** Genes are placed into functional categories.

The sets of differentially expressed genes, defined as signature lists, in the treated ovary groups compared to controls were identified using criteria as described in Methods. A total of 1081 genes were differentially expressed in ovaries treated with these known regulators of follicle assembly, suggesting these genes are involved in the ovarian primordial follicle assembly process. Whether the specific genes have an increase or decrease in expression is shown in Additional file 2: Table S1. Each treatment resulted in 50 to 303 genes being differentially expressed compared to controls. Interestingly, there were relatively few (5-10%) differentially expressed genes in common between the different treatments (Figure 2) as indicated for specific genes in Additional file 2: Table S1. Only one gene, the growth factor staniocalcin 1, was differentially expressed in as many as four different treatments, and no other genes were differentially expressed in more than three treatments (Additional file 2: Table S1). These genes in these signature lists were categorized into gene functional categories. The metabolism and transport, signaling, and receptors and binding proteins were predominant categories for all treatments (Figure 3 and Additional file 1: Table S1).

### Primordial follicle assembly pathway analysis

**Figure 4:**
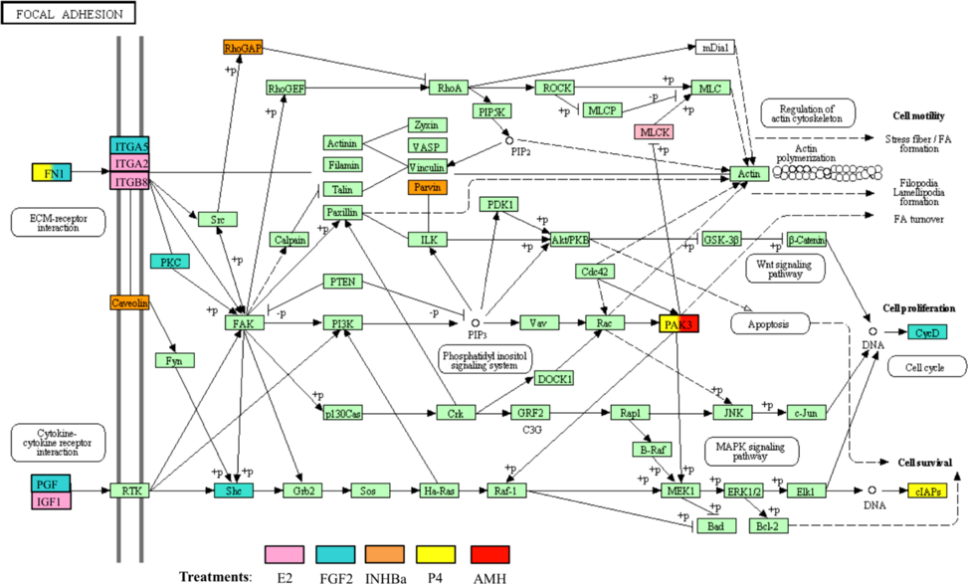
**Focal adhesion signaling pathway highlighting those genes that are differentially expressed compared to untreated control ovaries and identifying by color the treatment group(s) from which the differentially expressed genes came.** Adapted from KEGG pathway rno04510, Kanehisa Laboratories, Kyoto University, Japan.

The complete list of 1081 differentially expressed genes from the signature lists were correlated to curated cellular pathway and process gene lists from the KEGG database (see Methods) to identify physiological processes and pathways that may be regulated during follicle assembly. Pathways that were statistically over-represented within the differentially expressed gene lists included focal adhesion, chemokine signaling, cytochrome P450 metabolism, glutathione metabolism, ECM-receptor interaction and ribosomal components (Additional file 3: Table S2). There was a high degree of overlap of affected pathways between different treatments (Figure 2). The statistical analysis used both a hypergeometric probability calculation and Fishers exact test calculation to identify statistical likelihood of over-representation of differentially expressed genes in pathways (Additional file 3: Table S2). This analysis reduces the variable of data set size and potential for artifact generation by identifying those pathways with over-representation having differentially expressed genes from several treatments. As can be seen, not all pathways had statistical significance while others consistently did (Additional file 4: Figure S2). From 44% to 87% of affected pathways were common between treatments. According to Fisher’s Exact test the probability that our list of differentially expressed genes randomly overlaps with the pathways is negligible (~1/2000 chance), particularly since we had many genes falling in more than one pathway. As can be seen in Additional file 3: Table S2 and Figure 4, most affected pathways contained differentially expressed genes from several different treatments. As shown in the focal adhesion pathway, five of the factors affected genes in the same pathway (Figure 4). Therefore, each of these extracellular signaling factors that were used as treatments affected similar pathways via different genes. There were isolated exceptions to this trend. For example, all of the genes present in the ribosome process pathway were induced by activin A treatment, and four of five genes in the GABAergic synapse pathway were induced by FGF2 (Additional file 3: Table S2).

### Primordial follicle assembly bionetwork analysis

**Figure 5:**
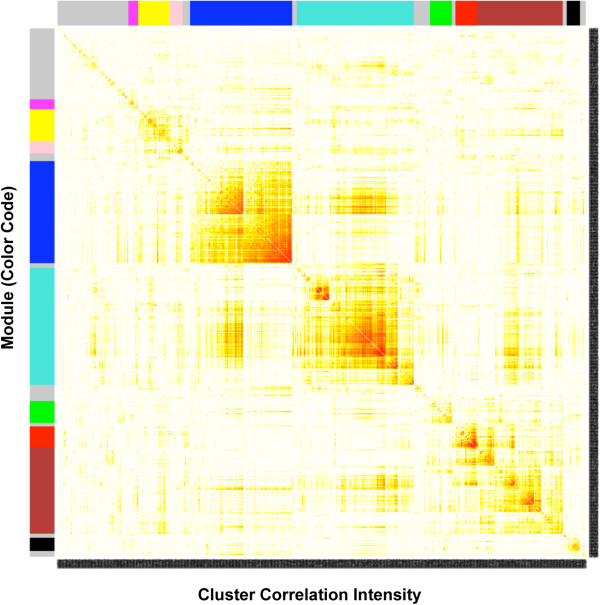
**Gene cluster analysis and corresponding gene modules.** A topological overlap matrix of the gene co-expression network consisting of the 1081 genes regulated by the various ovary treatments is shown. Genes in the rows and columns are sorted by an agglomerative hierarchical clustering algorithm (see Methods). The different shades of color signify the strength of the connections between the nodes (from white signifying not significantly correlated to red signifying highly significantly correlated). The hierarchical clustering and the topological overlap matrix indicate highly interconnected subsets of genes (modules). Modules identified are colored along both column and row and are boxed.

Gene expression level data from the entire set of 1081 differentially expressed genes was subjected to a cluster analysis to identify groups (i.e. modules) of genes whose expression is regulated in a coordinated and interconnected manner (see Methods). The cluster analysis scores each gene according to how well across different treatments its changes in gene expression are correlated with the changes in expression of every other gene. High connectivity scores indicate that expression of a particular gene changes in concert with that of many other genes. In addition, the cluster expression analysis identifies gene modules in which the member genes have similar changes in expression in response to the various experimental treatments. Such gene modules are often associated with specific biological processes [32]. To identify gene modules, a topological overlap matrix [37] was generated that reflected connectivity scores and sorted genes based on an agglomerative hierarchical clustering algorithm (see Methods). The topological overlap matrix map with gene modules color-coded for the nine modules identified is shown in Figure 5. The module to which each gene belongs can be found in Additional file 2: Table S1. The nine modules contained collectively 851 genes with the remaining 230 genes (colored as grey) not falling into any module. The list of differentially expressed genes in each module was correlated to signaling pathway and cellular process databases to determine if specific physiological processes were associated with particular modules (see Methods). Those pathways and processes statistically over-represented for each module are shown in Table 1. The turquoise module predominately contains genes coding for ribosomal components and genes involved in protein and glutathione metabolism. The blue module contains genes involved in processes related to tissue morphogenesis. The red module has genes involved with germ cells and meiosis. Some physiological processes, such as apoptosis and extracellular matrix function, were over-represented in several modules. However, in general the genes of different modules were over-represented in different cellular pathways and processes (Table 1).

**Table 1:** Gene category overlap with modules

**Module**	**Gene category**	**Gene**	**Over**
		**number**	**representation fisher**
		**module**	**fisher P-value**^**†**^
		overlap*	
Turquoise	Ribosome	10	7.52E-08
Turquoise	cytosolic ribosome	7	2.40E-06
Turquoise	Neural tube closure	4	0.00041
Turquoise	Negative regulation of binding	5	0.00045
Turquoise	Primary neural tube formation	4	0.00056
Turquoise	Protein biosynthesis	15	0.00071
Turquoise	Glutathione metabolism	5	0.0011
Blue	Response to oxidative stress	12	1.30E-06
Blue	Regulation of anatomical structure morphogenesis	13	2.20E-06
Blue	Mesoderm development	18	1.90E-05
Blue	Angiogenesis	6	2.20E-05
Blue	Response to carbohydrate stimulus	6	3.60E-05
Blue	Regulation of axonogenesis	6	5.00E-05
Blue	Membrane raft	9	7.80E-05
Blue	negative chemotaxis	3	1.00E-04
Blue	Axon guidance	6	3.70E-03
Blue	Glutathione metabolism	3	3.10E-02
Blue	Focal adhesion	6	3.20E-02
Blue	Fc gamma R-mediated phagocytosis	4	3.60E-02
Blue	MAPK signaling pathway	6	8.50E-02
Brown	Germ cell nucleus	3	7.00E-04
Brown	Male meiosis	3	0.0015
Brown	Neurite regeneration	3	0.0031
Brown	Condensed nuclear chromosome	3	0.0041
Brown	Other oncogenesis	3	0.0074
Brown	Cell cycle	4	0.026
Brown	Olfactory transduction	12	0.065
Yellow	Hematopoietic cell lineage	3	0.0027
Yellow	Positive regulation of apoptosis	5	0.009
Yellow	Coenzyme and prosthetic group metabolism	3	0.015
yellow	Mitochondrial lumen	3	0.018
Green	proteinaceous extracellular matrix	3	0.0072
Black	Extracellular matrix	4	0.00015
Black	extracellular region	7	0.00042
Black	Response to stress	8	0.002
Black	Cell communication	6	0.0029
Black	Negative regulation of apoptosis	4	0.0029
Pink	Apical plasma membrane	3	0.0011
Pink	Protein glycosylation	3	0.0018
Magenta	Calcium mediated signaling	2	0.0066

*Gene Number Module Overlap is the number of genes from that module that are in common with those in the listed physiological process or pathway (see Methods).

^†^Fisher’s Exact test was used to calculate a p-value reflecting the probability that the specified module and pathway/process would have listed number of overlapped genes.

**Figure 6:**
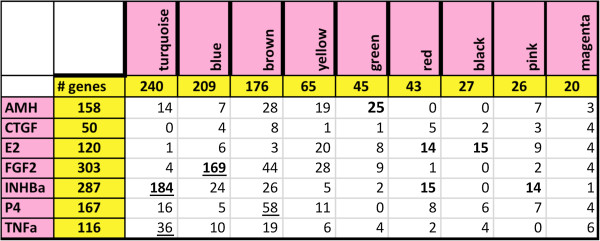
**Numbers of genes found to be in common when comparing genes differentially expressed in various ovary culture treatment groups (left column) with differentially expressed genes assigned to co-expression modules (top row).** Numbers in bold type indicate a high number of genes in common with a treatment compared to others for that module (column). Underlined numbers indicate a high number of genes in common with a module compared to others for that treatment (row).

Genes whose expression altered in response to treatments were correlated with the genes assigned to each module to determine if specific modules were heavily influenced by particular regulatory factors (Figure 6). In most cases, differentially expressed genes from each treatment group could be found in each of the modules. However, some modules had strikingly high numbers of genes in common with specific treatments. For example, among the 240 genes of the turquoise module and the 287 genes of the activin A treatment group, 184 genes were in common. Interestingly, these included 10 of the 11 differentially expressed genes that populated the ribosomal component process. In addition, among the 209 genes of the blue module and the 303 genes of the FGF2 treatment group, 169 genes were in common. Similarly, there were 58 genes in common between the brown module and the P4 treatment group, and these included 8 of the 19 genes that populated the olfactory transduction pathway (Figure 6).

### Primordial follicle assembly gene network analysis

In order to determine what functional relationships and gene networks exist between the differentially expressed genes identified, the complete list of 1081 genes was subjected to an automated unbiased analysis of published literature using Pathway Studio software (Elsevier Inc. Rockville, MD USA), (see Methods). A total of 326 genes were found to form a gene network that linked neighboring genes together with regulatory or binding relationships. While this network was too large and complex for easy visual interpretation (Additional file 4: Figure S2), inferences about the genes involved can be obtained. Genes with the greatest number of putative regulatory connections to neighbors in the network were considered to be important in controlling the follicle assembly process, either as upstream regulators of the assembly network or as downstream targets of the network. The genes with the most connections to neighbors in the network of 326 genes were *Il1b* (interleukin 1 beta; 144 connections), *Fn1* (fibronectin 1; 100 connections) and *Igf1* (insulin-like growth factor 1; 99 connections) (Additional file 4: Figure S2).

**Figure 7:**
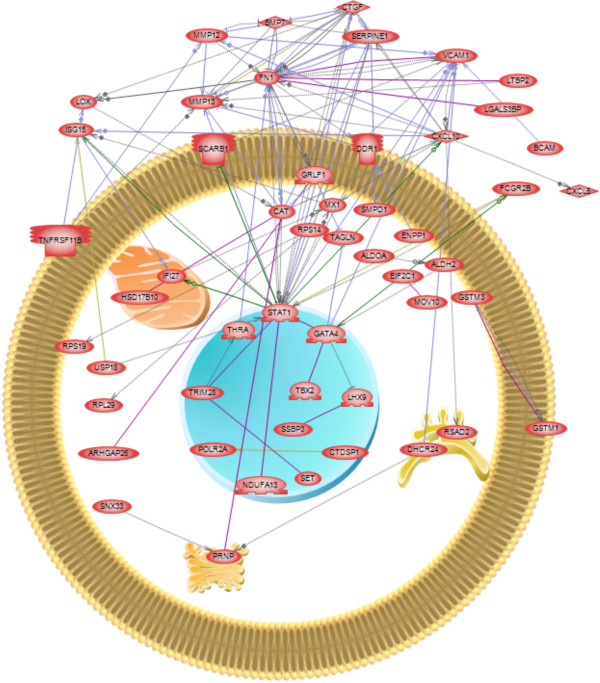
**Gene network of known relationships among those differentially expressed genes assigned to the turquoise module.** Node shapes code: oval – protein; diamond – ligand; irregular polygon – phosphatase; circle/oval on tripod platform – transcription factor; ice cream cone – receptor. Grey arrows represent regulation, lilac – expression, green – promoter binding, olive – protein modification, purple - binding. Plasma membrane, cell nucleus, mitochndria, golgi apparatus and endoplasmic reticulum are indicated for gene expression localization.

Each module of coordinately regulated genes was subjected to gene network analysis to determine which genes within a module formed a gene network as shown in Figure 7. A network for the turquoise module identified *Fn1, Stat1* (signal transducer and activator of transcription 1) and *Vcam* (vascular cell adhesion molecule 1) as having many connections, suggesting they may play a role in controlling the physiological processes mediated by the turquoise module. For the blue module (Additional file 5: Figure S3A) *Cav1* (caveolin), *Anxa2* (annexin A2), *F3* (coagulation factor 3, thromboplastin) and *Ccnd1* (cyclin D1) have the most neighbor connections. *F3* and *Ccnd1* are seen to be primarily the recipients of the regulatory relationships suggesting their regulation may be an important output of the blue module network. Although relatively small, the black module also formed a network of connected genes (Additional file 5: Figure S3B). The growth factors *Igf1* and *Il1b* have the most neighbor connections. The remaining modules did not form significant networks of genes related to each other, even though many of these modules had more genes than were present in the black module.

Each treatment signature list of differentially expressed genes was analyzed to determine which genes formed a gene network of regulatory relationships. For the genes of the E2 treatment group, *Igf1* is seen to have many neighbor connections (Additional file 6: Figure S4A). For the FGF2 treatment network *Vcam, Vim* (vimentin) and *Tgfb2* (transforming growth factor beta 2) have many neighbor connections, while *Ccnd1* and *F3* appear to be outputs of the network (Additional file 6: Figure S4B). For the activin A treatment group, the transcription factor *Stat1* and the growth factor *Cxcl10* have many connections, while *Fn1* appears to be an output (Additional file 6: Figure S4C).

**Figure 8:**
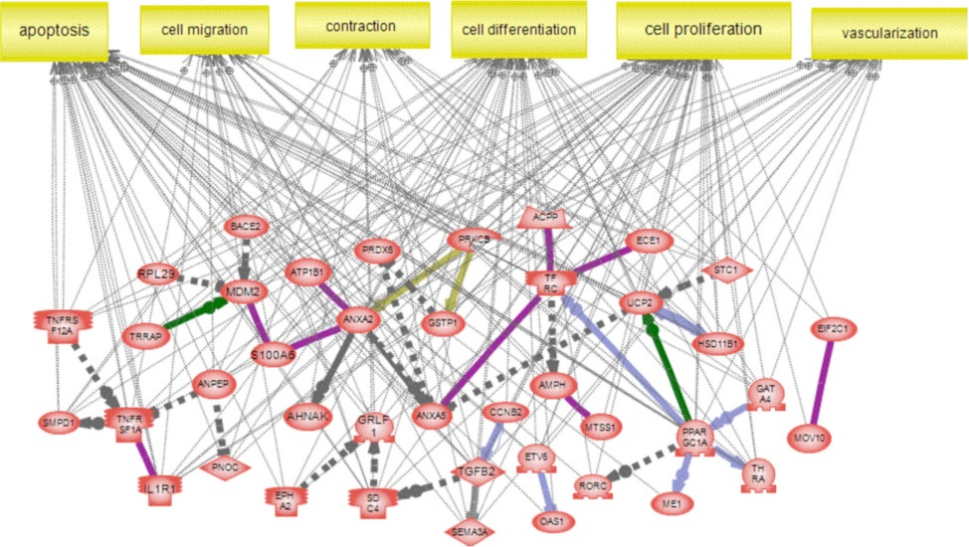
**Gene network of known relationships and associations with specific cell processes for those differentially expressed genes from the top 10% of each module that also had the most neighbor connections (see Table **2**for list).** Node shapes code: oval and circle – protein; diamond – ligand; irregular polygon – phosphatase; circle/oval on tripod platform – transcription factor; ice cream cone – receptor. Grey rectangles represent cell processes; arrows with plus sign show positive regulation/activation, arrows with minus sign – negative regulation/inhibition. Grey arrows represent regulation, lilac – expression, green – promoter binding, olive – protein modification.

In order to determine those genes whose actions are likely to be the most important in regulating the control of primordial follicle assembly, a combined approach was taken that determined the gene network associated with the most highly interconnected genes. For each module, the top 10% of genes with the highest connectivity index (k.in.) (i.e*.*, those that are the most tightly co-regulated within their module) were selected. These genes were then screened for whether they were present in the large network of 326 genes with known regulatory connections derived from the entire list of 1081 differentially expressed genes (Additional file 4: Figure S2). Those genes from the top 10% of each module (tightly co-expressed) that also had the most neighbor connections (regulatory relationships) are presented in Table 2. These included the transcriptions factors *Ppargc1a* and *Gata4*, the growth factor *Tgfb2*, the transferrin receptor (*Tfrc*), *Mdm2* and *Prkcb* (protein kinase C beta). These genes formed a regulatory network among themselves, and were associated with specific pathways and cellular processes. As can be seen in Figure 8, these genes have been previously shown to influence the processes of apoptosis, vascularization, contraction, cell migration, proliferation and differentiation.

**Table 2:** Gene connectivity ranking information

**Name /**	**Gene description**	**Local**
**symbol gene**		connectivity *
TNFRSF1A	Tumor necrosis factor receptor superfamily, member 1A	37
TGFB2	Transforming growth factor, beta 2	36
MDM2	Mdm2 p53 binding protein homolog (mouse)	27
GATA4	GATA binding protein 4	27
PPARGC1A	Peroxisome proliferator-activated receptor gamma, coactivator 1 alpha	26
PRKCB	Protein kinase C, beta	25
TFRC	Transferrin receptor (p90, CD71)	23
ANXA5	Annexin A5	20
ANXA2	Annexin A2	20
SDC4	Syndecan 4	16
LRP2	Low density lipoprotein-related protein 2	16
GRLF1	Glucocorticoid receptor DNA binding factor 1	15
GSTP1	Glutathione S-transferase pi 1	14
HSD11B1	Hydroxysteroid (11-beta) dehydrogenase 1	13
UCP2	Uncoupling protein 2 (mitochondrial, proton carrier)	12
THRA	Thyroid hormone receptor, alpha (erythroblastic leukemia viral (v-erb-a) oncogene homolog, avian)	10
ANPEP	Alanyl (membrane) aminopeptidase	10
IL1R1	Interleukin 1 receptor, type I	9
EPHA2	EPH receptor A2	9
CTSK	Cathepsin K	8
SMPD1	Sphingomyelin phosphodiesterase 1, acid lysosomal	8

*Local connectivity is the number of regulatory connections which that gene has with other genes, identified in published literature, within the gene network of 326 genes derived from the complete set of 1081 differentially expressed genes (see Additional file 4: Figure S2).

**Figure 9:**
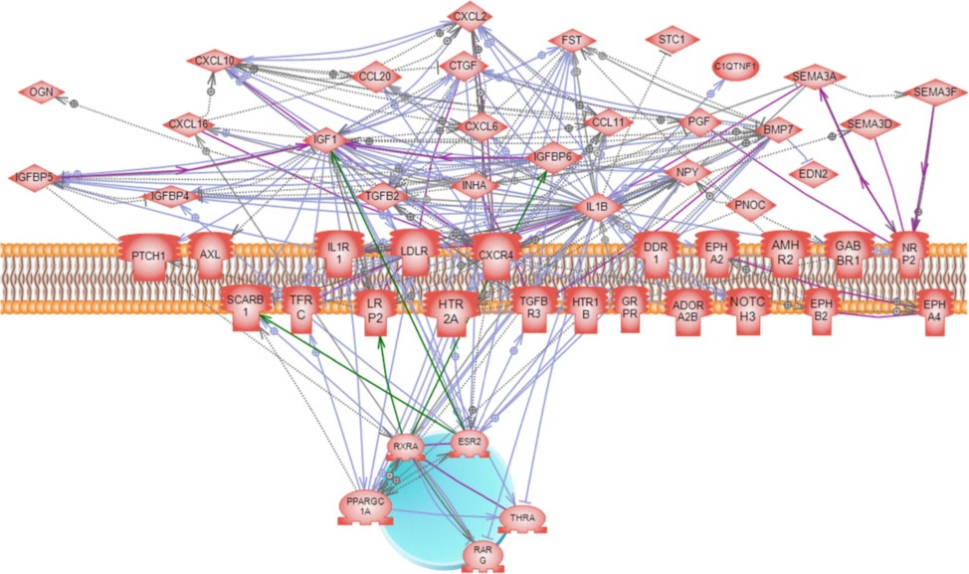
**Gene network of known relationships among those differentially expressed genes that were extracellular signaling factors, growth factors, growth factor binding proteins, growth factor receptors or hormone receptors (see Additional file****2****: Table S1: categories ‘Growth Factors’ and ‘Receptors &Binding Proteins’).** Node shapes code: oval – protein; diamond – ligand; circle/oval on tripod platform – transcription factor/hormone receptor; ice cream cone – receptor. Grey arrows represent regulation, lilac – expression, green – promoter binding, olive – protein modification, purple - binding. Plasma membrane and cell nucleus are indicated for gene expression localization.

Other groups of differentially expressed genes examined were the growth factor, hormone and receptor families. These genes provide novel regulatory factors to be investigated in future studies in their role in controlling ovarian primordial follicle assembly. A subset of the entire set of 1081 differentially expressed genes, comprised of growth factors, hormones and their receptors was evaluated for their ability to form a sub-network of regulatory connections. A sub-network of 52 genes was identified showing the regulatory connections between these growth factors and receptors (Figure 9).

### Primordial follicle assembly signaling pathway modulation and validation

**Figure 10:**
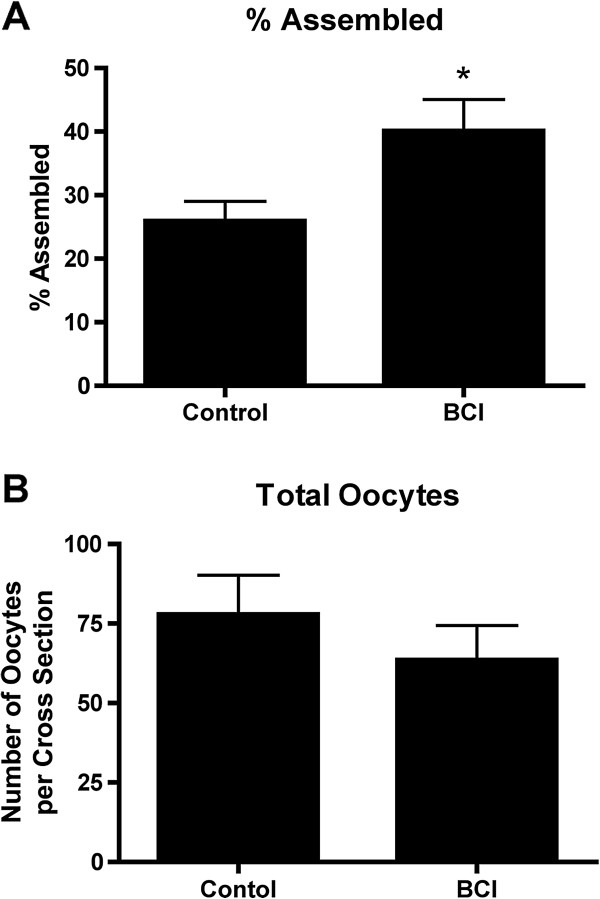
**Results of experiments in which 0-day old ovaries were cultured two days with or without BCI (an inhibitor of DUSP6 that results in increased ERK12/MAPK1 activity). A)** Proportion of oocytes still assembled into follicles. **B)** Total number of oocytes per ovarian cross-section after culture. * = p<0.05 compared to untreated vehicle-treated Control by Student’s *t*-test. Mean ± SEM from three different experiments is presented.

As described above, specific physiological processes and pathways are over-represented in the lists of differentially expressed genes identified in these studies (Table 1 and Additional file 3: Table S2), and so are predicted to be important in regulating follicle assembly. The MAPK signaling, focal adhesion and chemokine signaling pathways are over-represented in particular gene modules or in the global set of all 1081 differentially expressed genes. ERK1/2 (MAPK1) is a kinase that plays a prominent role in these pathways (Figure 4). ERK1/2 activity is inhibited by Dusp6 (dual specificity phosphatase 6; MKP-3) [38]. In order to test if these physiological pathways are in fact important to the assembly process, ovaries from 0-day old rats were treated in vitro for 2 days with the inhibitor of DUSP6: BCI ((E)-2-benzylidene-3-(cyclohexylamino)-2,3-dihydro-1H-inden-1-one [39,40]. Dusp6 inhibition resulted in a significant increase in the proportion of assembled follicles at the end of ovary culture with no effect on oocyte numbers (Figure 10). Therefore, alteration of ERK1/2 signaling in physiological pathways predicted to be important for follicle assembly resulted in a change in the rate of assembly of primordial follicles.

### Primordial follicle assembly regulated gene correlation with ovarian disease

**Figure 11:**
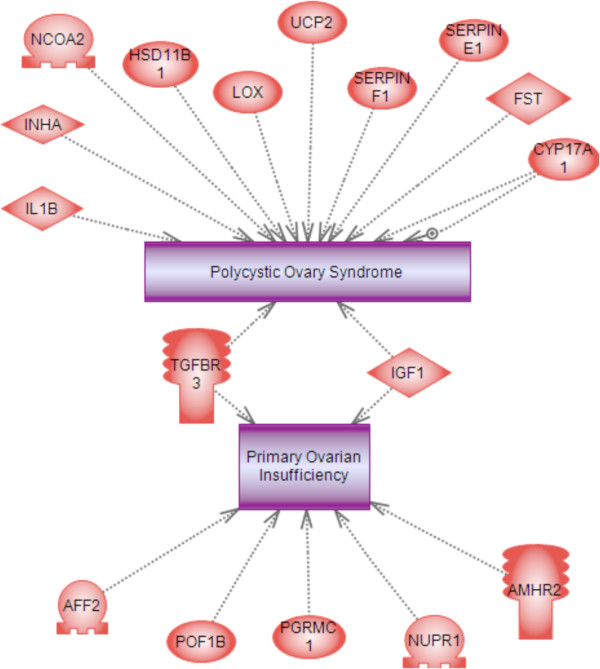
**Genes differentially expressed during follicle assembly that are also known to be associated with the adult-onset diseases primary ovarian insufficiency and polycystic ovarian syndrome.** Node shapes code: oval – protein; diamond – ligand; circle/oval on tripod platform – transcription factor; ice cream cone – receptor.

The final analysis identified those differentially expressed genes associated with ovarian disease. The genes within the differentially expressed gene lists (Additional file 2: Table S1) that have previously been shown to be linked in the literature with primary ovarian insufficiency (POI) and polycystic ovarian syndrome (PCOS) were identified (Figure 11). Seventeen genes associated with POI and PCOS were identified and two genes, Igf1 and Tgfbr3 were common to both POI and PCOS.

## Discussion

A systems biology approach was used to elucidate how regulatory factors alter gene expression to influence ovarian primordial follicle assembly. Neonatal rat ovaries were treated with different growth factors or hormones and changes in gene expression at the transcriptome level were assayed and analyzed. The strength of a systems biology approach is that it is unbiased and examines the genome wide complexity of gene expression to elucidate regulatory networks that control developmental processes. These genes are identified regardless of whether they have known functions consistent with the follicle assembly process or whether they have known functions at all. The unbiased systems analysis allows the complexity of the biology to be considered to elucidate the developmental process. Future investigations can now target identified genes to further characterize their specific actions in the networks regulating follicle assembly.

### Primordial follicle assembly growth factors and hormones investigated

Ovaries were treated with several different growth factors or hormones that affect the assembly process to get a more complete view of the gene expression changes accompanying primordial follicle assembly. A number of growth factors and hormones previously shown to influence primordial follicle assembly were selected including: AMH [1], CTGF [16], estradiol [17-21], progesterone [18,22,23], activin A [21], and TNFa [23,24]. The combined actions of factors on assembly has not been thoroughly investigated, but studies on follicle transition have shown combined stimulatory or inhibitory factors does not provide an additional response different from the individual factors [3]. All of the growth factors and corresponding receptors, as well as hormone receptors, associated with the factors used have been shown to be expressed in the ovary during primordial follicle assembly. For example, AMH is expressed in the stromal/interstitial cells of the 0 day ovary followed by expression later in development by the secondary and developing follicles [1]. Interestingly, some of the factors promote follicle assembly (CTGF, activin A, estradiol), while others (AMH, progesterone, TNFa, FGF2) inhibit follicle assembly. Therefore, both positive and negative regulation of follicle assembly is considered when characterizing the regulatory gene networks. Comparison of the stimulatory versus inhibitory factors did not show any major differences in regards to regulated genes or gene networks.

In addition to these known regulatory agents, a novel factor was examined and included in the analysis. Previous research suggested FGF2 may be a regulator of follicle assembly. A receptor for FGF2, FGFR2B, has previously been shown to be differentially expressed in 0-day old rat ovaries with oocyte nests compared to 4-day old ovaries that had completed assembly [30]. Treatment of neonatal rat ovaries with the known inhibitor of follicle assembly AMH resulted in differential expression of *Nudt6* (*nudix 6*) [41], which acts as an antisense inhibitor of *Fgf2* expression [42]. In order to determine if FGF2 would be included as a treatment in the current investigations, organ culture experiments were performed to empirically test the effects of FGF2 on follicle assembly. It was found that FGF2 acts as an inhibitor of follicle assembly (Figure 1). The magnitude of the inhibitory actions can be increased with an extended culture duration (four days), but then alterations in follicle number and ovarian morphology become confounders influencing data interpretation. A short 2-day culture was used to reduce these confounder effects. The optimal dose for the analysis of in vitro follicle assembly analysis was lower than that for the short-term 24-hour culture gene expression analysis, in part due to the negative feedback of the extended culture duration. The FGF2 was found to alter the expression of 303 genes with approximately 10% of these genes in common with the differentially expressed gene sets of the other features. Although the differentially expressed genes influenced by FGF were unique, 50–70 different pathways were affected and in common with other factors investigated, (Figure 2). Therefore FGF2 was included as one of the seven treatments used to perturb the neonatal rat ovary experimental system.

### Primordial follicle assembly differentially expressed genes

One thousand and eight-one genes were found to be differentially expressed compared to controls when results were combined across the different treatment groups. These genes were predominantly from the functional categories of metabolism and transport, signaling proteins and receptors. The genes differentially expressed in response to each growth factor or hormone treatment were compared across the different treatments. Interestingly, only a small proportion of differentially expressed genes (<10%) were found to be common between any two treatments (Figure 2). This is somewhat surprising in light of the fact that all the treatments affect the same process of follicle assembly. However, this same finding was observed in a systems biology investigation of genes regulating ovarian primordial to primary follicle transition [31]. In that study different treatments, all known to regulate the transition of arrested primordial follicles to developing primary follicles, were found to have few differentially expressed genes in common. Although neither this previous study nor the current study found a high degree of overlap between the specific treatment signature gene lists, there was a high degree of overlap among the specific signaling pathways and cellular processes impacted by the differentially expressed genes of each treatment (Figures 2 and 3). This indicates that all the treatments affect similar cellular pathways and processes as follicle assembly occurs (44% to 87%), but that each treatment affects different genes in those pathways. As shown in Additional file 2: Table S2, some pathways had a highly statistically significant over-representation of differentially expressed genes while others did not, suggesting the analysis was not unduly influenced by data set size or simply an artifact of the analysis. An example of this is shown in Figure 4 where five of the different factors affected different genes in the focal adhesion pathway. Similar observations have shown this phenomena in other biological systems [43]. Multiple input points into these cellular pathways and processes may allow for more precise regulation and for a more robust regulatory network in the face of disruptions.

A cluster analysis of coordinated gene expression grouped the differentially expressed genes into gene modules containing genes whose expression responded in concert to the different growth factor and hormone treatments. This approach of generating a weighted gene network and then clustering genes making use of a topological overlap matrix has been used extensively for uncovering biologically meaningful gene modules [31,32,44-46]. The gene modules identified in the current study were, on the whole, each enriched with genes associated with differing cellular pathways and processes (Table 1). For example, the turquoise module contains genes coding for ribosomal components, while the blue module contains genes involved in processes related to tissue morphogenesis, and the red module has genes associated with meiosis. This suggests that these modules of genes are each responsible for controlling distinct functions necessary for primordial follicle assembly. In contrast, most modules were enriched for genes involved in apoptosis and extracellular matrix function, perhaps underscoring the importance of these processes to follicle assembly. Oocyte apoptosis is known to have a vital role in the assembly of primordial follicles [3,6,12,13]. Identification and examination of the gene modules helps elucidate the molecular control of follicle assembly.

When genes whose expression changed in response to treatments were cross-matched with the genes assigned to each module, it was found that in most cases differentially expressed genes from each treatment group could be found in all the gene modules (Figure 6). This is consistent with the idea that all the treatments affect the same cellular processes, but that each treatment affects different genes within those pathways. However, it should be noted that in some cases a particular treatment shared a disproportionate number of genes within a specific module. For example, the activin A treatment resulted in differential expression of 287 genes, of which 184 were in common with the 240 genes assigned to the turquoise module, and these included almost all the genes that coded for ribosomal components. Therefore, in some cases signaling from a particular growth factor will induce a suite of genes that may be targeted toward specific physiological tasks.

### Primordial follicle assembly gene networks

In order to identify functional relations among differentially expressed genes, an analysis of published literature was used to detect connections among listed genes to form gene networks of putative regulatory relationships. Examination of these networks can yield inferences about how genes interact to regulate primordial follicle assembly, and can identify potentially important control points within these regulatory networks. When the entire list of genes found to be differentially expressed during follicle assembly was analyzed in this way it was found that the genes *Il1b* (interleukin 1 beta), *Fn1* (fibronectin 1) and *Igf1* (insulin-like growth factor 1) had the most regulatory connections to neighbors. These genes are considered important in controlling the follicle assembly process as either regulators of the assembly network or as downstream effectors of the network. However, it should be kept in mind that genes that have been extensively studied are more likely to have relationships with other genes in the published literature, and that un-studied genes may in fact be important. Nonetheless, gene networks of regulatory connections provide a good starting point toward understanding the control of processes such as follicle assembly.

Since the genes of each module may act in concert to accomplish distinct cellular processes during follicle assembly, each module was analyzed separately for gene networks of putative regulatory relationships. The genes of the turquoise, blue and black modules each formed distinct gene networks (Figure 7 and Additional file 5: Figure S3), implicating the genes *Fn1*, *Stat1* (signal transducer and activator of transcription 1), *Vcam* (vascular cell adhesion molecule 1), *Cav1* (caveolin), *Anxa2* (annexin A2), *F3* (coagulation factor 3), *Ccnd1* (cyclin D1), *Igf1* and *Il1b* as being important within their respective modules. Interestingly, genes of the brown, yellow, green, red, pink and magenta modules did not contain gene networks of known regulatory relationships, even though some of these modules contained many more genes than the black module, which did have such a network. This suggests that the genes within each of these modules may have as yet un-characterized regulatory relationships with each other. Furthermore, the genes of the red module were not found to be over-represented in association with any particular known cellular pathways or processes, and many of the red module genes are poorly characterized expressed sequence tags (ESTs). However, genes of the red module had relatively high connectivity scores (k. in.), indicating that this group of genes was quite tightly co-regulated (Figure 5). Observations suggest that groups of poorly characterized genes are likely playing important roles in primordial follicle assembly. Further research is needed to uncover the functions of these genes and their roles in developmental processes such as follicle assembly.

The most highly interconnected (k. in.) genes from each module were compared to databases of genes present in specific cellular pathways and processes. This group of highly interconnected genes was found to be over-represented in the processes of apoptosis, cell migration, cell differentiation and cell proliferation (Figure 8). This is consistent with the activities that occur during follicle assembly and supports the idea that these highly interconnected genes contribute to follicle assembly. Genes were also over-represented in the process of vascularization which is not known to be a part of follicle assembly. Investigations into the role vascularization plays in follicle assembly are suggested in future studies.

Analysis of a specific gene sub-network of differentially expressed genes in the growth factor, hormone and receptor functional gene categories identified a large number of such regulatory signaling factors that appear to regulate ovarian primordial follicle assembly (Figure 9). Many of these growth factors and receptors in this sub-network have multiple connections with each other, indicating that these genes are known to regulate other signaling factors within the sub-network. The growth factors IL1B, IGF1 and CXCL10, and the receptor CXCR4 have the most connections to other genes, so these are candidates in the regulation of follicle assembly to investigate in future studies. The CXCR4 and IGF1 genes have been shown to be involved in primordial follicle transition [47,48], but not previously been associated with assembly.

### Primordial follicle assembly modulation and pathway validation

Analyses of the differentially expressed genes of the current study implicate specific physiological pathways and gene networks as being important to the follicle assembly process. In order to test the validity of some of these predictions, organ culture experiments were preformed in which neonatal rat ovaries were treated with BCI. BCI has the effect of increasing ERK1/2 (MAPK1) activity *via* inhibition of DUSP6 [38,39]. ERK1/2 plays a prominent role in the MAPK signaling, focal adhesion and chemokine signaling pathways, all of which were implicated as important to follicle assembly (Figure 4, Additional file 3: Table S2, Table 1). BCI-treated ovaries with increased ERK1/2 activity were shown to have an increase in assembled follicles (Figure 10), supporting the predicted role of these physiological pathways in primordial follicle assembly. This experiment helps validate the systems biology approach used in the current study.

### Primordial follicle assembly and ovarian disease

Since follicle assembly provides each female mammal with the pool of primordial follicles from which their ovulated eggs are derived, abnormal follicle assembly could result in defective primordial follicles that may lead to a reduced follicle pool size. This in turn can lead to infertility and the cessation of reproduction early in life, as is seen in women with primary ovarian insufficiency (POI). Women with POI deplete their pool of primordial follicles prior to age 40 and undergo early menopause [14,15]. Forty-nine genes that have been implicated in POI in humans have been compiled and are listed with the Ovarian Kaleidoscope Database (http://ovary.stanford.edu/). Seven of these genes were found to be in common with the 1081 differentially expressed genes found in the current study to be associated with follicle assembly (significant at p<0.05 by Fisher’s Exact test). These genes were *Tgfbr3* (transforming growth factor beta receptor type 3), *Amhr2* (anti-Müllerian hormone receptor type 2), *Pgrmc1* (progesterone receptor membrane component 1), *Nupr1* (nuclear protein transcriptional regulator 1), *Pof1b* (premature ovarian failure 1b), IGF1 and AFF2 (AF4/FMR2 family, member 20). AMH, progesterone and *Pgrmc1* are known to play roles in follicle assembly [1,17,18,22,23]. It is notable that *Pof1b*, the gene named for its association with premature ovarian failure (i.e. POI), is linked to follicle assembly in the current study. These observations suggest that some cases of POI may have abnormal follicle assembly as an underlying cause. In addition to specific gene links with POI, a number of links were also made to polycystic ovarian syndrome (PCOS) (Figure 11). PCOS is the most predominant female reproductive disease affecting 7-18% of the female population [49]. A number of the differentially expressed genes identified in the current study correlated to previously known genes linked to PCOS (Figure 11). Observations suggest abnormal ovarian primordial follicle assembly may be a component of POI and PCOS later in life as some of the genes involved are in common. Future analysis of these genes and the gene networks identified is anticipated to help elucidate the molecular etiology of POI and PCOS, as well as provide novel therapeutic targets.

## Conclusions

In summary, a systems biology experimental approach can provide an unbiased global view of the relationships important to a particular developmental process. For the primordial follicle assembly process the systems approach evaluated genes that were differentially expressed in response to growth factor and hormone treatments. It was found that different treatments all affected similar cellular pathways and processes, but that each treatment affected expression of different genes within those pathways. Cluster analyses identified modules of coordinately regulated genes and the different modules appear to accomplish distinct cellular functions during follicle assembly. The regulatory gene networks identified provide predictions about important regulatory genes, signaling pathways and cellular processes involved in ovarian primordial follicle assembly. An organ culture experiment in which ovaries were treated to increase ERK1/2 activity confirmed some of the predicted physiological pathways were in fact important in follicle assembly regulation. The regulatory genes and gene networks identified as controlling primordial follicle assembly, when disrupted or altered, were suggested to be linked to the etiology of ovarian diseases such as primary ovarian insufficiency POI and polycystic ovarian syndrome PCOS. Future investigations can now utilize the observations from this systems approach to further elucidate the molecular control of ovarian primordial follicle development and associated diseases.

## Methods

### Ovarian organ culture

Zero-day old female Sprague–Dawley rats (Harlan Laboratories, Inc., USA) were euthanized within six hours after birth according to Washington State University IACUC approved (#02568) protocols and the ovaries removed and cultured whole as described previously [50]. Zero-day old rat ovaries contain primarily oocytes in nests, prior to being assembled into follicles. For ovary culture experiments in which ovarian RNA was collected, 2–3 ovaries per well were cultured for one day in the absence (controls) or presence (treated) of either AMH (human Anti-Müllerian hormone)(50 ng/ml, R&D Systems Inc., USA), FGF2 (rat Fibroblast growth factor 2)(50 ng/ml, R&D Systems Inc., USA), CTGF (human Connective Tissue Growth Factor)(500 ng/ml, PeproTech Inc., NJ USA), TNFa (rat Tumor Necrosis Factor alpha)(1ng/ml, R&D Systems Inc., USA), activin A (human/mouse/rat activin beta-A homodimer)(100 ng/ml, R&D Systems Inc., USA), E2 (Estradiol)(1×10^-6^M, Sigma-Aldrich, USA), or P4 (Progesterone)( 1×10^-6^M, Sigma-Aldrich, USA). After only one day of culture there are few morphological differences between control and treated ovaries, so measurements of whole-ovary gene expression reflect differences in RNA transcription, rather than differing proportions of cell types due to differing cell proliferation between treatments. After culture the 2–3 ovaries receiving the same treatment from one culture well were pooled and homogenized in one ml Trizol™ reagent (Sigma-Aldrich, USA), then stored at −70°C. There were three different biological experiments (biological replicates) for each of the seven treatment compounds, and seven replicates of the controls, for a total of 28 RNA samples.

In order to determine the effect of FGF2 on primordial follicle assembly, ovaries were cultured as above for two days in the absence or presence of FGF2 (50 ng/ml). Similarly, in order to determine the effect of increased ERK1/2 signaling on follicle assembly, ovaries were cultured in the presence or absence of BCI (1μM; Sigma-Aldrich #B4313). After 2 days culture ovaries were fixed with Bouin’s solution, paraffin embedded, sectioned onto microscope slides and stained with hematoxylin and eosin as described previously [50].

### Morphometric analysis

The number of oocytes at each developmental stage was counted and averaged in two serial sections from the largest cross-section through the center of the ovary. Oocytes in ovarian cross sections were classified as unassembled, or as assembled into primordial (stage 0), or developing follicles (stages 1–4: early primary, primary, transitional and preantral) as previously described [1,51]. Oocytes in nests are contiguous with other oocytes, without intervening stromal cells. An oocyte was still considered to be part of a nest if, for any region of its perimeter, one quarter of its circumference or more was contiguous with other oocytes. Primordial follicles consist of an oocyte arrested in prophase I of meiosis that is encapsulated by squamous (i.e*.* flattened) pregranulosa cells. Early transition primary follicles have initiated development (i.e., undergone primordial to primary follicle transition) and contain at least two cuboidal granulosa cells. Primary and preantral follicles exhibit one or more complete layers of cuboidal granulosa cells [30,52]. Hematoxylin/eosin stained ovarian sections were analyzed at 400× magnification using light microscopy. Degenerating red eosin-stained oocytes were not counted. Oocytes in which the cell nucleus was not clearly visible in the plane of section were not counted.

### RNA preparation

RNA was isolated from whole rat ovaries after homogenization in one ml Trizol™ reagent (Sigma-Aldrich, USA), according to manufacturer’s instructions. Two or three ovaries from the same culture well (from different rat pups) and receiving the same treatment were pooled and homogenized together. Homogenized samples were stored at −70°C until the time of RNA isolation. After isolation from Trizol, RNA was further purified using RNeasy MinElute Cleanup Kits (Qiagen, USA) and stored in aqueous solution at −70°C.

### Microarray analysis

The microarray analysis was performed by the Genomics Core Laboratory, Center for Reproductive Biology, Washington State University, Pullman, WA using standard Affymetrix reagents and protocol. Briefly, mRNA was transcribed into cDNA with random primers, cRNA was transcribed, and single-stranded sense DNA was synthesized which was fragmented and labeled with biotin. Biotin-labeled ssDNA was then hybridized to the Rat Gene 1.0 ST microarrays containing 27,342 transcripts (Affymetrix, Santa Clara, CA, USA). Hybridized chips were scanned on Affymetrix Scanner 3000. CEL files containing raw data were then pre-processed and analyzed with Partek Genomic Suite 6.3 software (Partek Incorporated, St. Louis, MO) using an RMA GC-content adjusted algorithm. Comparison of all array histogram graphs demonstrated that the data for 27 of 28 chips were similar and appropriate for further analysis. One chip, from a P4-treated sample, was an outlier and so was discarded (Additional file 1: Figure S1). In addition, a batch effect associated with RNA processing date was noted and incorporated into the analysis. The data from the remaining 27 chips were again pre-processed and analyzed as a group, with the RNA processing batch used as a blocking factor.

The microarray quantitative data involves over 10 different oligonucleotides arrayed for each gene and the hybridization must be consistent to allow for a statistically significant quantitative measure of gene expression and regulation. In contrast, a quantitative PCR procedure only uses two oligonucleotide primers, and primer bias is a major factor in this type of analysis. Therefore, we did not attempt to use PCR based approaches as we feel the microarray analysis is more accurate and reproducible without the primer bias of PCR based approaches.

All microarray CEL files (MIAME compliant raw data) from this study have been deposited with the NCBI gene expression and hybridization array data repository (GEO, http://www.ncbi.nlm.nih.gov/geo) (GEO Accession number: Pending), all arrays combined with one accession number, and can be also accessed through http://www.skinner.wsu.edu. For gene annotation, Affymetrix annotation file RaGene-1_0-st-v1.na32.rn4.transcript.csv was used unless otherwise specified.

### Gene bionetwork analysis

The cluster coordinated expression analysis was restricted to genes differentially expressed between the control and the treatment groups based on previously established criteria: (1) fold change of group means ≥1.2 or ≤0.83; (2) *T* test p-value ≤0.05 compared to control; and (3) absolute difference of group means ≥10. The less stringent cutoff for fold change avoids loss of important genes at such an early stage of analysis since these candidate genes will go through subsequent systems-level coexpression network and pathway analyses that can further filter noisy signal, as we have shown in the previous study [53]. The union of the differentially expressed genes from the different treatments resulted in 1081 genes (i.e*.* Affymetrix probesets) being identified and used for constructing a weighted gene co-expression network [32,44]. Unlike traditional un-weighted gene co-expression networks in which two genes (nodes) are either connected or disconnected, the weighted gene co-expression analysis assigns a connection weight to each gene pair using soft-thresholding and thus is robust to parameter selection. The weighted network analysis begins with a matrix of the Pearson correlations between all gene pairs, then converts the correlation matrix into an adjacency matrix using a power function *f(x) = x*^*β*^. The parameter *β* of the power function is determined in such a way that the resulting adjacency matrix (i.e., the weighted co-expression network), is approximately scale-free. To measure how well a network satisfies a scale-free topology, we use the fitting index proposed by Zhang & Horvath [32] (i.e., the model fitting index *R*^*2*^ of the linear model that regresses *log(p(k))* on *log(k)* where k is connectivity and *p(k)* is the frequency distribution of connectivity). The fitting index of a perfect scale-free network is 1. For this dataset, we select the smallest *β ( = 7)* which leads to an approximately scale-free network with the truncated scale-free fitting index *R*^*2*^ greater than 0.75. The distribution *p(k)* of the resulting network approximates a power law: *p(k)~k*^−1.29^.

To explore the modular structures of the co-expression network, the adjacency matrix is further transformed into a topological overlap matrix [37]. The topological overlap between two genes reflects not only their direct interaction, but also their indirect interactions through all the other genes in the network. Previous studies [32,37] have shown that topological overlap leads to more cohesive and biologically meaningful modules. To identify modules of highly co-regulated genes, we used average linkage hierarchical clustering to group genes based on the topological overlap of their connectivity, followed by a dynamic cut-tree algorithm to dynamically cut clustering dendrogram branches into gene modules [54]. A total of nine modules were identified and the module size was observed to range from 20 to 240 genes.

To distinguish between modules, each module was assigned a unique color identifier, with the remaining, poorly connected genes colored grey. In this type of map, the rows and the columns represent genes in a symmetric fashion, and the color intensity represents the interaction strength between genes (Figure 5). This connectivity map highlights the fact that differentially expressed genes fall into distinct network modules, where genes within a given module are more interconnected with each other (blocks along the diagonal of the matrix) than with genes in other modules. There are several network connectivity measures, but one particularly important one is the within module connectivity (k.in). The k.in of a gene was determined by taking the sum of its connection strengths (co-expression similarity) with all other genes in the module to which the gene belonged.

In order to compile a shorter list of the most tightly co-regulated genes from each module, the top 10% of genes from each module with highest k.in. scores (connectivity within module) were selected. Additional genes were added from each module, above 10%, if those genes had k. in. scores >8. If the list for any module did not include enough named genes (i.e. genes that were not EST’s) to equal 10% of the module size, then additional genes with the highest k.in. scores were added until 10% named genes was achieved.

### Pathway analysis

Lists of differentially expressed genes for each regulatory factor treatment, as well as for each module generated in the network analysis, were analyzed for KEGG (Kyoto Encyclopedia for Genes and Genome, Kyoto University, Japan) pathway enrichment using Pathway-Express, a web-based tool freely available as part of the Onto-Tools (http://vortex.cs.wayne.edu) [55]. Additionally, gene lists were analyzed using the KEGG website (http://www.genome.jp/kegg/pathway.html). KEGG pathways were considered ‘impacted’ and were included in analyses for this manuscript if three or more differentially expressed genes were present within a KEGG pathway. Statistical over-representation of differentially expressed genes within a pathway was determined by Fischer’s exact test for two by two contingency tables, and by calculating hypergeometric probability of obtaining exactly the listed number of genes in common with that pathway.

Global literature analysis of various gene lists was performed using Pathway Studio (Ariadne, Genomics Inc. Rockville MD) software, which performs pathway and interaction analysis and identifies genes that have regulatory or binding relationships. Pathway Studio was also used to identify cellular functions and diseases (including polycystic ovarian syndrome) linked in the published literature to the genes in these lists. In addition, Pathway Studio was used to determine over-represented physiological processes for gene lists based on KEGG, PANTHER (Protein ANalysis THrough Evolutionary Relationships) and NCBI GO (National Center for Biotechnology Information Gene Ontology) databases.

## Abbreviations

POI: Primary ovarian insufficiency; PCOS: Polycystic ovarian disease syndrome; AMH: Anti-Müllerian hormone; CTGF: Connective tissue growth factor; E2: Estradiol; P4: Progesterone; TNFa: Tumor necrosis factor alpha; BDNF: Brain derived neurotrophic factor; KITL: Kit ligand; GDF9: Growth differentiation factor-9; FGF2: Fibroblast growth factor-2; k.in.: Connectivity index.

## Competing interest

The authors declare that no competing interests exist.

## Authors’ contribution

MKS designed the study. EN performed the ovary cultures and microarray analysis and network design. BZ performed the bionetwork analysis and bioinformatics. EN wrote the manuscript and all authors edited the manuscript. All authors read and approved the final manuscript.

## Supplementary Material

Additional file 1: Figure S1 Sample histograms and box plots for ovary RNA sample microarray signal values prior to pre-processing and normalization. Note that one of the P4-treated samples was an outlier, and was discarded. X-axis shows hybridization intensity value. Y-axis (Hybridization Frequency) shows the number of genes having a given hybridization intensity. Click here for file

Additional file 2: Table S1 Differentially expressed genes: A) Genes influenced by treatment with anti-Müllerian hormone (AMH). B) Genes influenced by treatment with CTGF. C) Genes influenced by treatment with FGF2. D) Genes influenced by treatment with ActivinA. E) Genes influenced by treatment with P4. F) Genes influenced by treatment with TNFa. G) Genes influenced by treatment with E2. Click here for file

Additional file 3: Table S2 Treatment and module differentially expressed genes correlated to cellular pathways and processes. Click here for file

Additional file 4: Figure S2 Gene network of known relationships among all 1081 genes found to be differentially expressed in treated *versus* Control ovaries. Genes with the greatest number of connections (relationships) to other genes have enlarged gene symbols. Network is derived from an un-biased search of literature using Pathway Studio™. Node shapes code: oval – protein; diamond – ligand; irregular polygon – phosphatase; circle/oval on tripod platform – transcription factor; ice cream cone – receptor. Grey arrows represent regulation, lilac – expression, green – promoter binding, olive – protein modification, purple - binding. Click here for file

Additional file 5: Figure S3 Gene network of known relationships among differentially expressed genes assigned to specific co-expression modules. A) Blue module. B) Black module. Network is derived from an un-biased search of literature using Pathway Studio™. Node shapes code: oval – protein; diamond – ligand; irregular polygon – phosphatase; circle/oval on tripod platform – transcription factor; ice cream cone – receptor. Grey arrows represent regulation, lilac – expression, green – promoter binding, olive – protein modification, purple - binding. Click here for file

Additional file 6: Figure S4 Gene network of known relationships among genes differentially expressed in ovaries receiving specific treatments, compared to controls. A) E2 (estrogen). B) FGF2. C) Activin A. Network is derived from an un-biased search of literature using Pathway Studio™. Node shapes code: oval – protein; diamond – ligand; irregular polygon – phosphatase; circle/oval on tripod platform – transcription factor; ice cream cone – receptor. Grey arrows represent regulation, lilac – expression, green – promoter binding, olive – protein modification, purple - binding. Click here for file
