# Supplementary figures and images for: Gene bionetworks that regulate ovarian primordial follicle assembly
Source: BMC Genomics. 2013 Jul 23;14:496. doi: 10.1186/1471-2164-14-496 (PMC3726361; doi:10.1186/1471-2164-14-496)

Supplemental Figure S1 (Color)

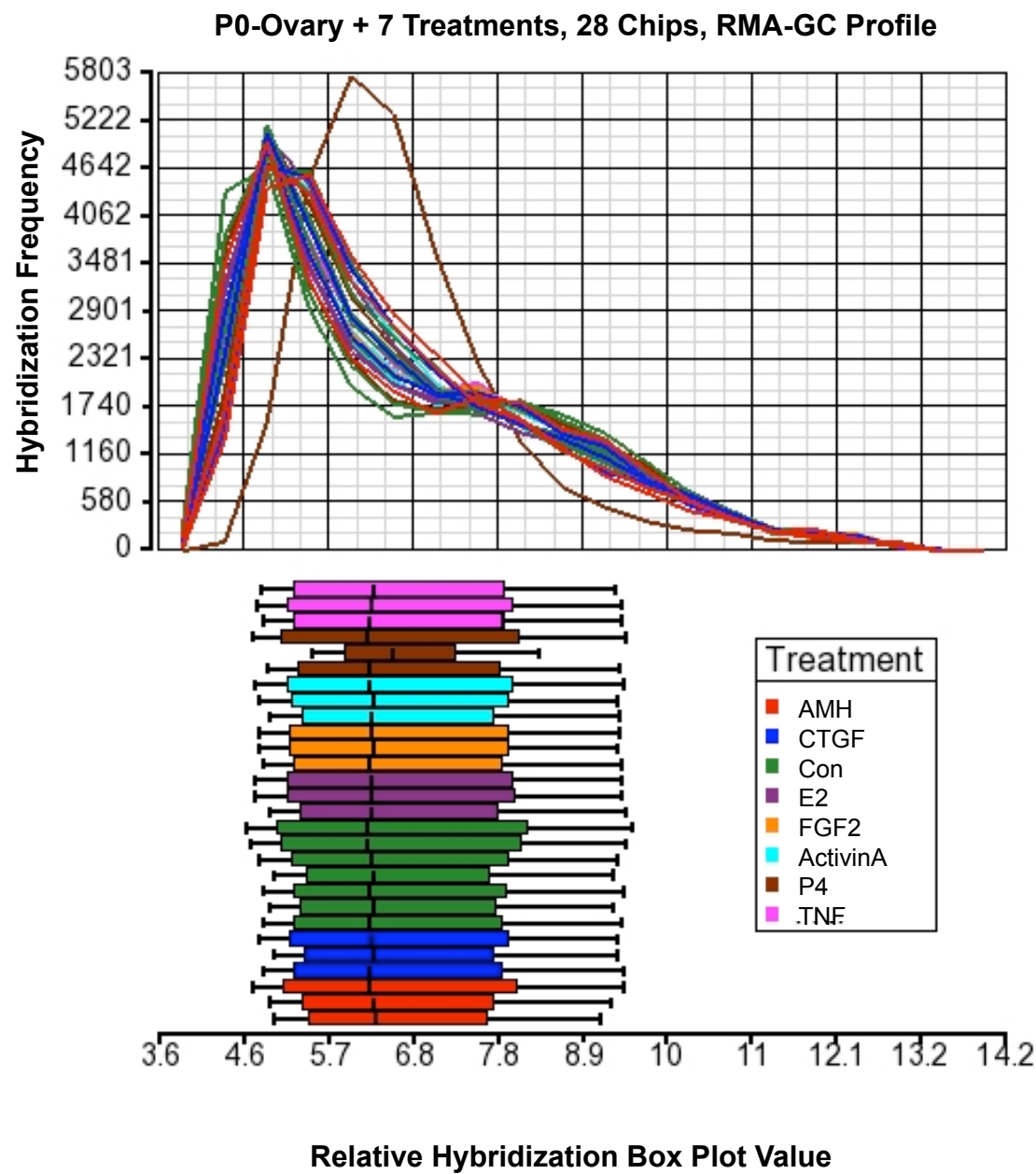

Supplement: Additional file 1: Figure S1 — Sample histograms and box plots for ovary RNA sample microarray signal values prior to pre-processing and normalization. Note that one of the P4-treated samples was an outlier, and was discarded. X-axis shows hybridization intensity value. Y-axis (Hybridization Frequency) shows the number of genes having a given hybridization intensity. [file 1471-2164-14-496-S1.pdf]

Supplemental Figure S2 (Color)

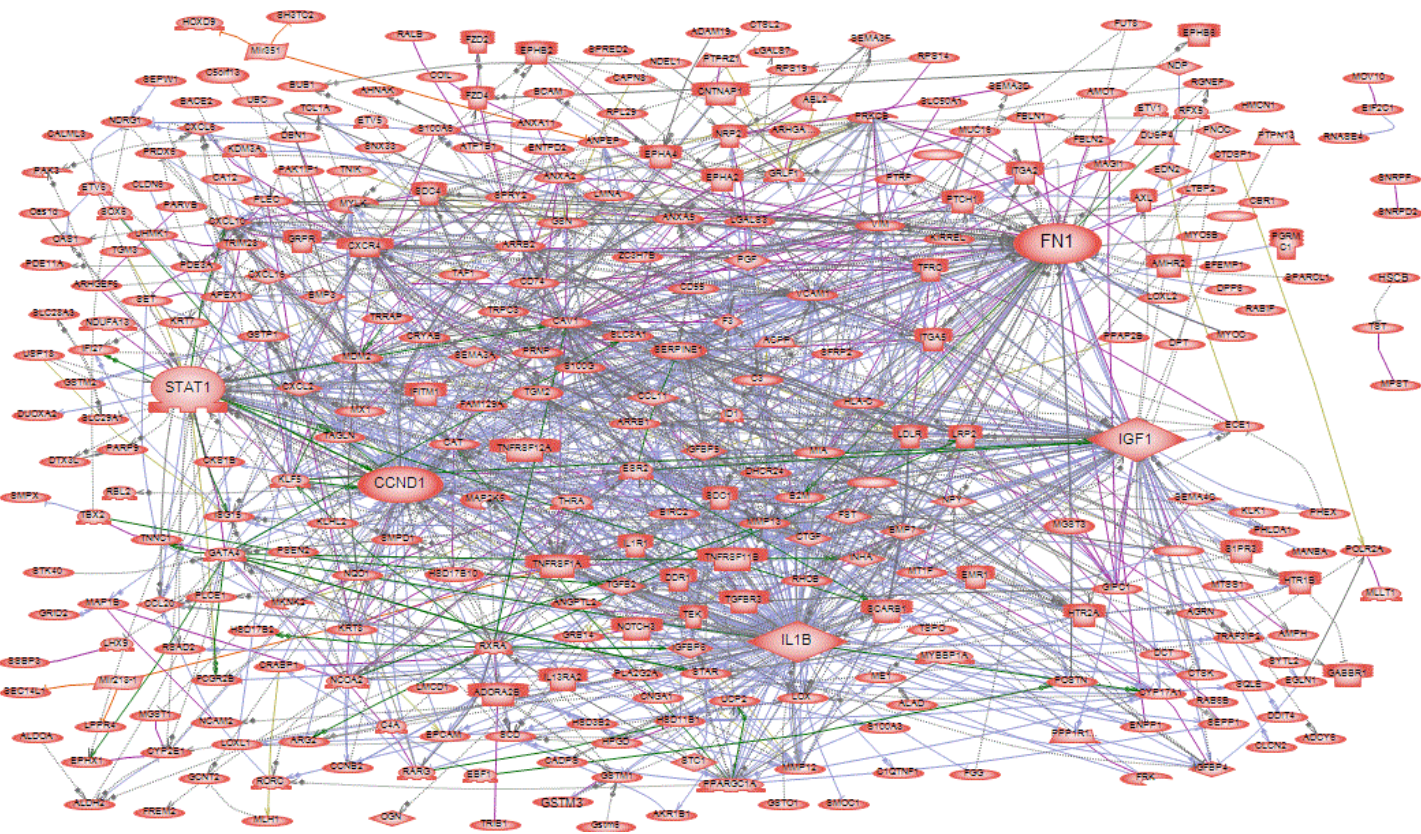

Supplement: Additional file 4: Figure S2 — Gene network of known relationships among all 1081 genes found to be differentially expressed in treated versus Control ovaries. Genes with the greatest number of connections (relationships) to other genes have enlarged gene symbols. Network is derived from an un-biased search of literature using Pathway Studio™. Node shapes code: oval – protein; diamond – ligand; irregular polygon – phosphatase; circle/oval on tripod platform – transcription factor; ice cream cone – receptor. Grey arrows represent regulation, lilac – expression, green – promoter binding, olive – protein modification, purple - binding. [file 1471-2164-14-496-S4.pdf]

Supplemental Figure S3

A

Blue Module

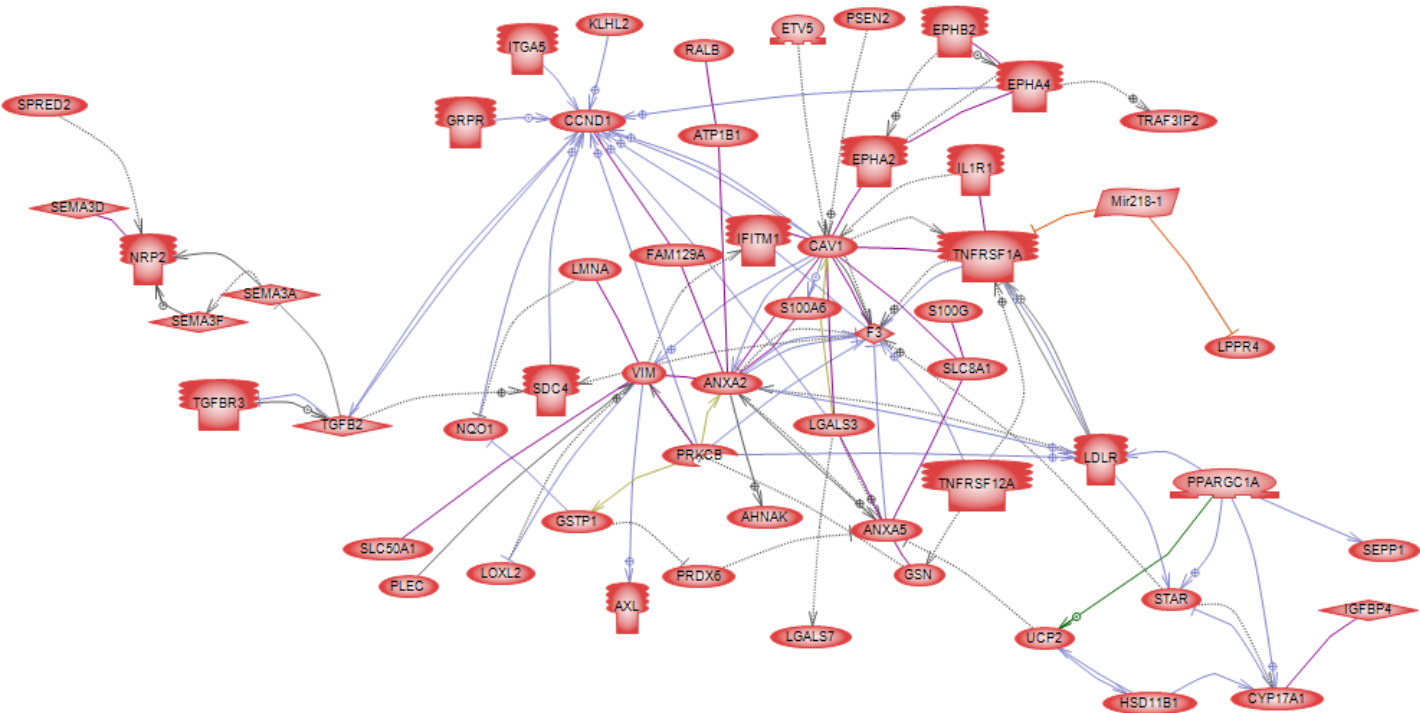

B

Black Module

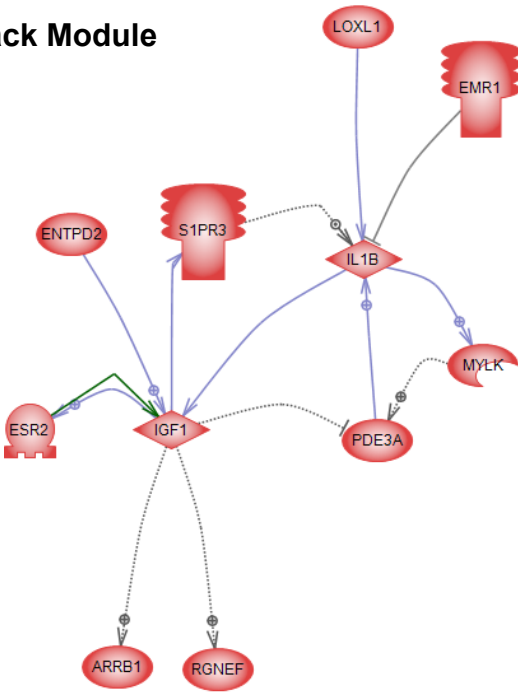

Supplement: Additional file 5: Figure S3 — Gene network of known relationships among differentially expressed genes assigned to specific co-expression modules. A) Blue module. B) Black module. Network is derived from an un-biased search of literature using Pathway Studio™. Node shapes code: oval – protein; diamond – ligand; irregular polygon – phosphatase; circle/oval on tripod platform – transcription factor; ice cream cone – receptor. Grey arrows represent regulation, lilac – expression, green – promoter binding, olive – protein modification, purple - binding. [file 1471-2164-14-496-S5.pdf]

**A** **E2**

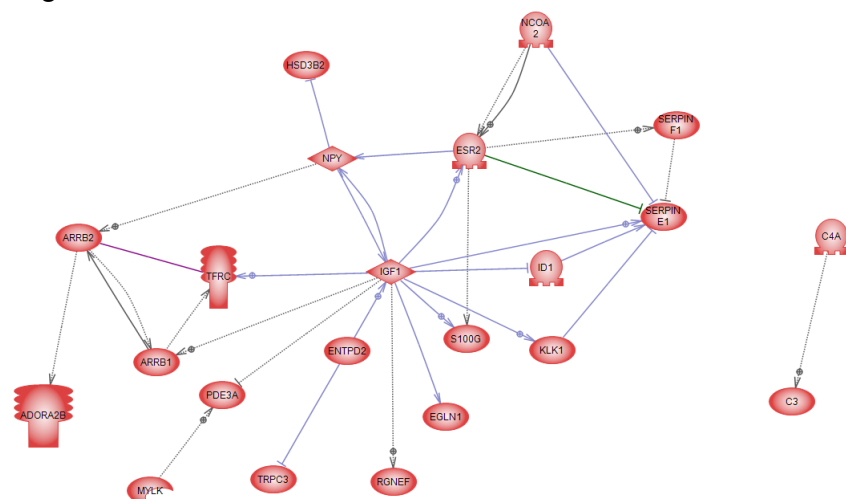

**B** FGF2

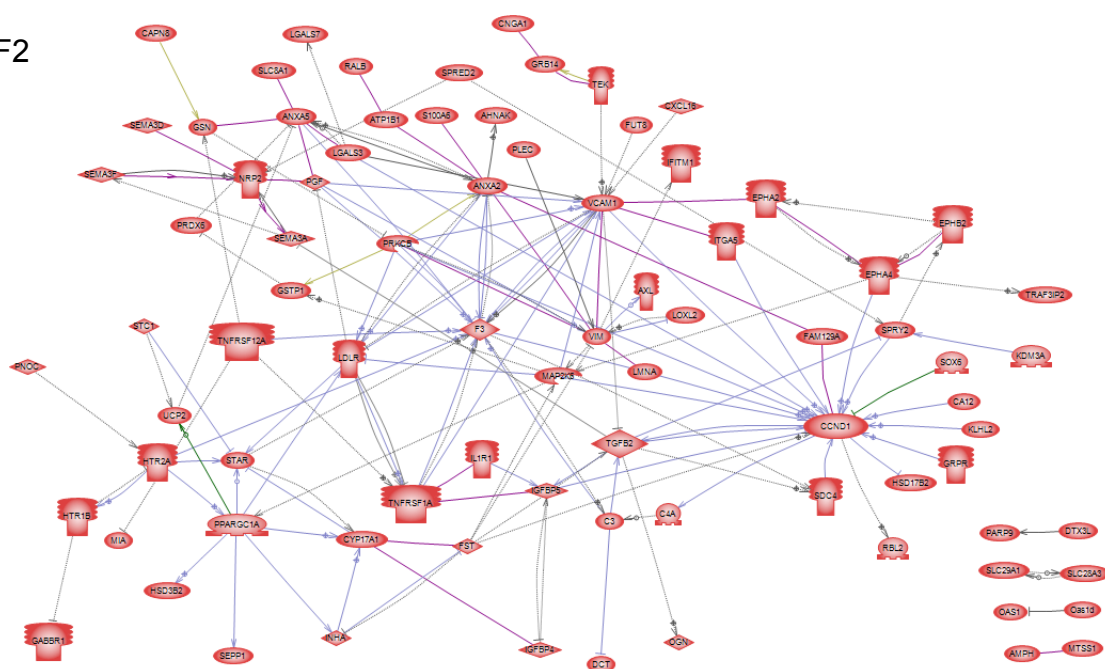

**C** Activin A

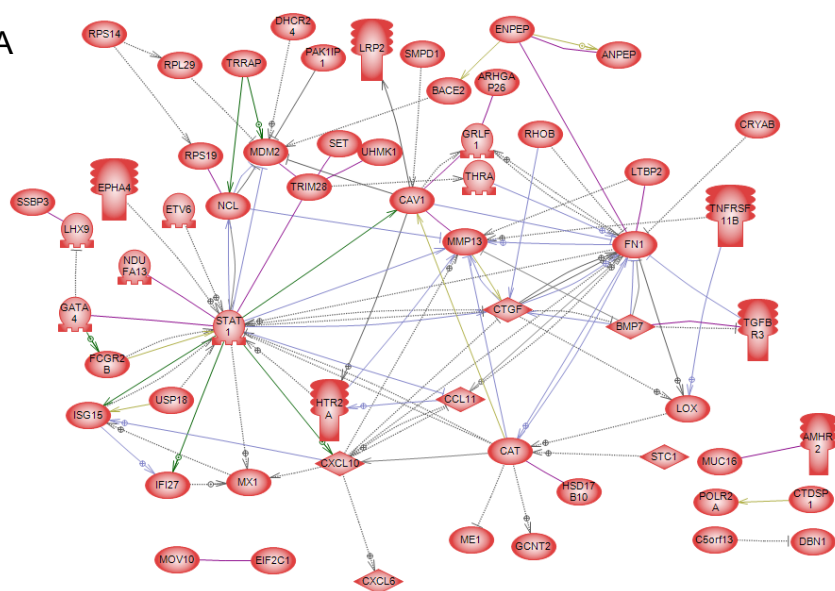

Supplement: Additional file 6: Figure S4 — Gene network of known relationships among genes differentially expressed in ovaries receiving specific treatments, compared to controls. A) E2 (estrogen). B) FGF2. C) Activin A. Network is derived from an un-biased search of literature using Pathway Studio™. Node shapes code: oval – protein; diamond – ligand; irregular polygon – phosphatase; circle/oval on tripod platform – transcription factor; ice cream cone – receptor. Grey arrows represent regulation, lilac – expression, green – promoter binding, olive – protein modification, purple - binding. [file 1471-2164-14-496-S6.pdf]
