# Supplementary material for: Gene bionetworks that regulate ovarian primordial follicle assembly
Source: BMC Genomics. 2013 Jul 23;14:496. doi: 10.1186/1471-2164-14-496 (PMC3726361; doi:10.1186/1471-2164-14-496)
Supplement: Additional file 2: Table S1 — Differentially expressed genes: A) Genes influenced by treatment with anti-Müllerian hormone (AMH). B) Genes influenced by treatment with CTGF. C) Genes influenced by treatment with FGF2. D) Genes influenced by treatment with ActivinA. E) Genes influenced by treatment with P4. F) Genes influenced by treatment with TNFa. G) Genes influenced by treatment with E2. [file 1471-2164-14-496-S2.pdf]

Supplemental Table S1

*Fold change is the ratio of the gene's expression level in treated samples divided by that in control samples.
†Mean difference is the difference between the gene's mean expression level in treated samples minus that in control samples, in arbitraty units after microarray data pre-processing and normalization (see Methods).

**A. Genes influenced by treatment with anti-Müllerian hormone (AMH)**						
**Gene Symbol**	**Entrez ID**	**Probeset ID Fold Change***	**Mean Diff†**	**Module**	**Present in Other Lists**	**Gene Title**
**Apoptosis**						
Cd55	64036	1.449956759	188.6537491	yellow		Cd55 molecule
Pcbp4	363133	0.76741369	-53.35736142	grey	TNFa	poly(rC) binding protein 4
**Cell Cycle**						
Ccnb2	363088	1.284435087	284.6970392	brown		cyclin B2
Ccnyl1	316452	1.233737667	117.4246841	brown		cyclin Y-like 1
Frk	79209	1.251608606	71.39146834	grey		fyn-related kinase
Oip5	499873	1.238255277	17.6751442	green		Opa interacting protein 5
RGD1563620	310511	1.252785149	157.76747	brown		similar to retinoblastoma binding protein 4
**Cytoskeleton-ECM**						
Coil	50998	1.217886491	22.10967467	brown		coilin
Eml4	313861	1.362650036	33.40056408	grey	TNFa	echinoderm microtubule associated protein like 4
Gipc1	83823	0.831775946	-32.71571306	brown		GIPC PDZ domain containing family, member 1
Krt8	25626	1.250839633	37.98726377	grey		keratin 8
Lgals3bp	245955	0.807573489	-59.76135739	turquoise		lectin, galactoside-binding, soluble, 3 binding protein
Map1b	29456	1.265097113	30.14655882	brown		microtubule-associated protein 1B
Muc16	315451	1.376163809	128.0966178	yellow	ActivinA	mucin 16, cell surface associated
Myo1e	25484	1.254036608	80.15684726	brown		myosin IE
Myo5b	25132	1.204299814	39.5290838	green		myosin Vb
Pls1	315926	1.233404161	76.52386216	brown		plastin 1 (I isoform)
Sdc1	25216	1.312248005	66.33663166	green		syndecan 1
Sorbs2	114901	1.258850778	154.2825114	turquoise	ActivinA, TNFa	sorbin and SH3 domain containing 2
Zp4	282833	1.469037386	258.1057294	green		zona pellucida glycoprotein 4
**Development**						
Giot1	171090	0.797569891	-34.72151236	grey		gonadotropin inducible ovarian transcription factor 1 /// zinc finger protein 347
LOC499234	499234	1.230496793	388.934445	brown		similar to NACHT, leucine rich repeat and PYD containing 14-like
LOC691658	691658	1.299704641	84.87790937	green		similar to anterior pharynx defective 1b homolog
Mustn1	290553	1.204537459	11.63286889	grey		musculoskeletal, embryonic nuclear protein 1
Pkd2l2	291683	1.361955735	17.99132924	grey		polycystic kidney disease 2-like 2
Sh3tc2	307393	1.245581913	19.6470528	yellow		SH3 domain and tetratricopeptide repeats 2
**DNA Repair**						
Apex1	79116	1.210452724	111.5452712	grey		APEX nuclease (multifunctional DNA repair enzyme) 1
Neil3	290729	1.218548471	11.13592566	turquoise		nei endonuclease VIII-like 3 (E. coli)
Trex1	100049583	0.675801778	-36.01601964	grey		three prime repair exonuclease 1
**Electron Transport**						
Hscb	360826	0.807348173	-122.8456654	grey		HscB iron-sulfur cluster co-chaperone homolog (E. coli)
**Epigenetics**						
Cxxc5	291670	0.798543098	-126.8700549	pink		CXXC finger 5
**EST's**						
RGD1311378	296408	0.75208955	-58.35361359	grey		similar to RIKEN cDNA 2010011I20
RGD1561277	497911	1.30619837	38.44448896	yellow		RGD1561277
---	---	0.826970292	-39.72170929	grey		---
---	---	1.591676624	17.57008138	grey		---
---	---	0.557459453	-34.3372808	grey		---
---	---	1.202829863	38.05213426	brown		---
---	---	1.339636079	19.71053434	magenta		---
---	---	1.276194633	105.0765637	yellow		---
---	---	1.641345178	23.45264738	green		---
---	---	1.329054202	24.9163447	turquoise		---
---	---	1.230834467	21.08988836	grey		---
---	---	1.276536425	177.1621676	green		---
---	---	1.243360998	17.14644711	blue		---
---	---	1.235420548	79.2685755	grey		---

---	---	0.815773222	-12.98248561	turquoise		---
---	---	0.790454792	-11.6709461	grey		---
---	---	1.334345274	45.61800381	yellow		---
---	---	1.301077196	27.00684969	turquoise		---
---	---	1.270446422	11.77779782	grey		---
---	---	1.407978385	13.08688309	grey		---
---	---	1.22088565	42.47135057	grey		---
---	---	1.22088565	42.47135057	grey		---
---	---	1.221675748	49.86628938	brown		---
---	---	1.279745752	75.96709206	green		---
---	---	0.739615419	-22.95006468	green		---
---	---	1.622456879	35.20746901	grey		---
---	---	1.200918267	10.44079577	grey		---
---	---	1.319249501	104.8995462	grey		---
---	---	0.805424552	-16.31685454	grey		---
---	---	1.277359003	14.73027557	grey		---
---	---	1.226635101	32.11564592	turquoise		---
---	---	0.758765095	-121.8355629	green		---
---	---	1.329753333	98.4888906	grey		---
---	---	0.744378856	-10.17526357	yellow		---
---	---	1.258588471	16.01906229	green		---
---	---	1.312134486	90.9138399	grey		---
---	---	0.822261677	-90.21568822	green		---
---	---	1.230685411	31.12775193	brown		---
---	---	1.513758596	23.84899282	grey		---
---	---	1.212085568	30.06130697	turquoise		---
---	---	0.818399074	-16.10382562	green		---
---	---	1.229366085	19.41481098	grey		---
---	---	1.24178966	47.92688561	grey		---
**Golgi Apparatus**						
B3gnt9	291958	1.210267434	11.62730488	green		UDP-GlcNAc:betaGal beta-1,3-N-acetylglucosaminyltransferase 9
Cog8	291990	0.804833397	-17.04926453	grey		component of oligomeric golgi complex 8
Mgat4a	367252	1.22593793	75.15324845	brown		mannosyl (alpha-1,3-)-glycoprotein beta-1,4-N-acetylglucosaminyltransferase, isozyme A
**Growth Factors**						
Cxcl1	81503	1.263898552	69.01020479	magenta		chemokine (C-X-C motif) ligand 1 (melanoma growth stimulating activity, alpha)
Stc1	81801	1.703172244	171.8066011	pink	E2, FGF2, ActivinA, P4	stanniocalcin 1
**Immune Response**						
Cd24	25145	1.201927845	81.44350851	yellow	CTGH	CD24 molecule
Cd9	24936	1.296316471	138.9109684	brown		CD9 molecule
Ly6c	56778	1.30205742	63.5752168	blue	FGF2, ActivinA	Ly6-C antigen
Mal2	362911	1.369847812	90.66381503	yellow	FGF2	mal, T-cell differentiation protein 2
**Metabolism & Transport**						
Akr1b1	24192	1.216711198	364.5743017	green		aldo-keto reductase family 1, member B1 (aldose reductase)
Aqp9	65054	1.319056807	37.03678388	blue	FGF2	aquaporin 9
Arg2	29215	1.211826719	10.57766019	brown		arginase type II
Atp6v0a4	296981	1.364857161	45.42246165	pink	E2, ActivinA, P4	ATPase, H+ transporting, lysosomal V0 subunit A4
Bcmo1	114106	1.222909267	17.98557407	green		beta-carotene 15,15'-monooxygenase 1
Car12	363085	1.234545191	27.04036266	brown	FGF2, P4	carbonic anyhydrase 12
Doc2b	81820	0.673606785	-73.47609163	grey		double C2-like domains, beta
Hpgd	79242	1.260306527	14.90024379	pink	E2	hydroxyprostaglandin dehydrogenase 15 (NAD)
Iah1	298917	1.267724432	416.8159253	green		isoamyl acetate-hydrolyzing esterase 1 homolog (S. cerevisiae)
Mpst	192172	0.792182505	-47.30194795	turquoise		mercaptopyruvate sulfurtransferase
Nmnat1	298653	1.207908785	63.43025364	brown		nicotinamide nucleotide adenylyltransferase 1
Oas1d	304508	1.312475361	22.01442751	yellow	FGF2	2'-5' oligoadenylate synthetase 1D
Phkg2	140671	1.269388584	57.6879476	green		phosphorylase kinase, gamma 2 (testis)
Serinc5	170907	1.205165258	191.2070984	green		serine incorporator 5
Slc15a3	246239	0.801933428	-10.28125205	brown		solute carrier family 15, member 3
Slc30a6	298786	1.294150633	95.14698839	brown		solute carrier family 30 (zinc transporter), member 6
Slc39a1	361986	0.812170605	-117.4643589	grey		solute carrier family 39 (zinc transporter), member 1
Slc7a6	307811	1.205197439	54.92784168	yellow	E2	solute carrier family 7 (cationic amino acid

						transporter, y+ system), member 6
Slc7a9	116726	0.746421538	-12.5451657	grey		solute carrier family 7 (cationic amino acid transporter, y+ system), member 9
Sqle	29230	1.315720659	80.72584287	grey		squalene epoxidase
**Miscellaneous & Unknown**						
Aff2	293922	1.254255513	110.1430375	yellow		AF4/FMR2 family, member 2
Gpatch4	295228	1.215274937	17.94476706	green		G patch domain containing 4
Lrrc8a	311846	0.81300204	-56.76981509	grey		leucine rich repeat containing 8 family, member A
Mfsd5	315329	0.784916753	-28.96856965	grey		major facilitator superfamily domain containing 5
RGD1307722	362824	0.806977863	-65.47605873	grey	CTGH, TNFa	similar to hypothetical protein MGC20700
RGD1308616	362573	0.798993448	-36.48842114	grey	TNFa	similar to KIAA0467 protein
RGD1560755	361165	1.258677699	21.65845609	turquoise		similar to D8Ertd354e protein
RGD1562037	498764	1.337049641	45.54637397	green		similar to OTTHUMP00000046255
RGD1564257	500595	0.801660468	-29.29753631	grey		similar to hypothetical protein FLJ32825
RGD1565493	500853	1.220820588	14.9746838	grey	FGF2	similar to DKFZP434I092 protein
Tmem50b	360698	1.209875694	305.6672393	brown	FGF2	transmembrane protein 50B
**Proteolysis**						
Psmd9	161475	1.200991592	21.64353189	green		proteasome (prosome, macropain) 26S subunit, non-ATPase, 9
Serpine2	29366	1.30564197	89.73844826	brown		serine (or cysteine) peptidase inhibitor, clade E, member 2
Usp49	316211	1.227425387	30.82085154	green		ubiquitin specific peptidase 49
**Receptors & Binding Proteins**						
Adora2b	29316	1.202767847	12.18856954	grey	E2	adenosine A2B receptor
Axl	308444	1.267011117	100.4838724	blue	FGF2	Axl receptor tyrosine kinase
Ddr1	25678	0.821890912	-42.20975024	turquoise		discoidin domain receptor tyrosine kinase 1
Htr2a	29595	1.369092038	170.3716945	yellow	FGF2, ActivinA	5-hydroxytryptamine (serotonin) receptor 2A
Lrp2	29216	1.352726103	47.60115199	pink	ActivinA, P4	low density lipoprotein-related protein 2
Olr1084	404958	1.240255985	11.22550037	grey		olfactory receptor 1084
Olr1619	361033	0.744892921	-19.64755449	grey	ActivinA	olfactory receptor 1619
Rarg	685072	0.816022509	-14.65539256	brown		retinoic acid receptor, gamma
Rxra	25271	0.816947192	-50.22452165	grey		retinoid X receptor alpha
S100g	24249	1.543212192	46.34978358	blue	E2	S100 calcium binding protein G
Sorcs3	294043	0.650143582	-53.96958097	grey	E2	sortilin-related VPS10 domain containing receptor 3
Vom2r16	690691	0.768549338	-63.02459038	grey	ActivinA	vomeronasal 2 receptor, 16
**Signaling**						
Ano10	301111	0.776379792	-26.81538048	grey		anoctamin 10
Arhgef6	363509	1.235917652	55.16585842	grey		Rac/Cdc42 guanine nucleotide exchange factor (GEF) 6
Calml3	307100	0.685540563	-127.682783	magenta		calmodulin-like 3
Cks1b	499655	1.273768874	382.8481592	grey		CDC28 protein kinase regulatory subunit 1B
Dtx3l	498089	1.423548943	55.81590955	yellow	FGF2	deltex 3-like (Drosophila)
Gng13	685451	1.312186408	13.29440076	yellow	FGF2	guanine nucleotide binding protein (G protein), gamma 13
Gng5	79218	1.310396594	92.35924791	grey		guanine nucleotide binding protein (G protein), gamma 5 /// brix domain containing 5
Pak3	29433	1.242562068	179.7098921	yellow	P4	p21 protein (Cdc42/Rac)-activated kinase 3
Pde1b	29691	1.214769718	33.03341436	blue		phosphodiesterase 1B, calmodulin-dependent
Ppp1r15a	171071	0.83213355	-11.38733762	grey		protein phosphatase 1, regulatory (inhibitor) subunit 15A
Rab6b	363123	1.260941876	26.04034554	pink	ActivinA	RAB6B, member RAS oncogene family
Rabif	304807	1.207236251	98.91955974	brown		RAB interacting factor
Tcl1a	690575	1.420811917	236.2085078	green		T-cell leukemia/lymphoma 1A
**Transcription**						
Id3	25585	0.735368861	-107.6544317	turquoise		inhibitor of DNA binding 3
Klf5	84410	0.822916766	-10.71292804	green		Kruppel-like factor 5
Mlh1	81685	1.295973876	87.77899983	brown		mutL homolog 1 (E. coli)
Nrbp2	680451	1.259797427	93.72008687	turquoise		nuclear receptor binding protein 2
Nudt5	361274	1.278099142	85.56649521	brown		nudix (nucleoside diphosphate linked moiety X)-type motif 5
Pole3	298098	1.298285562	26.77100173	yellow		polymerase (DNA directed), epsilon 3 (p17 subunit)
Ptrf	287710	0.791553607	-25.28003068	brown		polymerase I and transcript release factor
Zfp185	689949	1.224676183	13.27201987	yellow	E2	zinc finger protein 185
Zfp39	303173	1.367659893	100.0988856	blue		zinc finger protein 39
Zfp9	100158232	1.27664531	27.24868728	turquoise		zinc finger protein 9
**Translation & Protein Modification**						

Hecw1	291209	1.205148221	24.93407245	pink		HECT, C2 and WW domain containing E3 ubiquitin protein ligase 1
Rbm46	310548	1.256217938	65.36430751	brown	TNFa	RNA binding motif protein 46
Snrpd2	680309	1.243231368	140.1843003	grey		small nuclear ribonucleoprotein D2 polypeptide
Tgm3	366189	0.767954979	-14.48336314	grey		transglutaminase 3, E polypeptide
Thyn1	300470	1.260814324	114.3405456	yellow		thymocyte nuclear protein 1

B. Genes influenced by treatment with CTGF						
Gene Symbol	Entrez ID	Fold Change	Mean Diff	Module	Present in Other Lists	Gene Title
**Cell Cycle**						
Ppp2r2b	60660	0.772824945	-59.28889205	pink		protein phosphatase 2 (formerly 2A), regulatory subunit B, beta isoform
**Development**						
Rsbn1l	311987	1.207588342	29.41621848	grey		round spermatid basic protein 1-like
Tex19.1	498033	0.692667141	-25.71126921	grey		testis expressed gene 19.1
**EST's**						
---	---	1.628571659	123.9651465	red		---
---	---	1.628571659	123.9651465	red		---
---	---	1.266384592	12.35719711	blue		---
---	---	0.832565936	-10.2341197	grey		---
---	---	0.821392324	-12.44358325	magenta		---
---	---	1.222107968	27.89828991	black		---
---	---	0.827555553	-41.82891974	grey		---
---	---	0.831670516	-19.68731767	brown		---
---	---	0.793349285	-128.7781619	grey		---
---	---	1.217991646	19.57347221	grey		---
---	---	1.248420892	19.06666414	grey		---
---	---	1.604619263	13.93073189	grey		---
---	---	1.232651424	14.16740144	grey		---
---	---	0.825819943	-23.18111222	grey		---
---	---	1.62790157	31.7031872	grey		---
---	---	1.37330258	23.62457099	grey		---
---	---	0.805461445	-283.1386683	magenta		---
---	---	0.829302528	-21.03369728	brown		---
---	---	0.812451418	-74.52564077	grey		---
---	---	0.775757775	-95.79031416	blue		---
---	---	1.347689939	132.5298197	red		---
---	---	1.347689939	132.5298197	red		---
---	---	1.288490848	18.45084936	grey		---
---	---	0.826327575	-20.63515994	blue		---
---	---	0.795195749	-478.9222552	grey		---
---	---	0.769035475	-30.20397834	green		---
**Immune Response**						
Cd24	25145	0.814084756	-110.7092834	yellow	AMH	CD24 molecule
**Metabolism & Transport**						
Gstm6	499688	1.260047017	38.28870404	grey		glutathione S-transferase, mu 6
Tmc4	308310	0.805967249	-10.99506874	grey		transmembrane channel-like 4
**Miscellaneous & Unknown**						
Fam63b	363089	1.211045876	22.54038431	brown		family with sequence similarity 63, member B
LOC100361234	100361234	1.236784146	29.43705235	blue	FGF2	BTB (POZ) domain containing 19-like
RGD1307569	360695	0.826076626	-18.06828968	grey		similar to Protein C21orf63 homolog precursor
RGD1307722	362824	1.205572224	46.67756941	grey	AMH, TNFa	similar to hypothetical protein MGC20700
**Receptors & Binding Proteins**						
Olr1339	300639	0.827344642	-12.97650381	brown		olfactory receptor 1339
Olr1608	290006	0.830234775	-17.18696577	brown		olfactory receptor 1608
Olr1629	405389	0.787453646	-15.47445199	brown		olfactory receptor 1629
Olr422	296684	0.814939327	-15.9981571	brown		olfactory receptor 422
RGD1562717	363767	1.20808993	94.94473365	magenta	FGF2	similar to ABI gene family, member 3 (NESH) binding protein
**Signaling**						
LOC100361238	100361238	1.209958489	44.16192411	magenta		RasGEF domain family, member 1B
Plekhg1	679812	1.224662194	21.87067799	pink		pleckstrin homology domain containing, family G (with RhoGef domain) member 1
Purg	361162	1.202624974	15.33639815	brown		purine-rich element binding protein G
Tnik	294917	0.821911527	-43.7942579	pink		TRAF2 and NCK interacting kinase
**Transcription**						
Epcam	171577	0.81085107	-75.18772615	red		epithelial cell adhesion molecule
Fat4	310341	1.217898491	79.29607809	black		FAT tumor suppressor homolog 4 (Drosophila)
Zdhhc22	299211	1.217447012	11.51430572	grey		zinc finger, DHHC-type containing 22
**Translation &**						

**Protein Modification**						
Rbm4b	474154	1.218788609	89.91963084	grey		RNA binding motif protein 4B /// RNA binding motif protein 4
RGD1559743	316048	0.798859733	-13.25434823	grey		similar to 40S ribosomal protein S16

C. Genes influenced by treatment with FGF2						
Gene Symbol	Entrez ID	Fold Change	Mean Diff	Module	Present in Other Lists	Gene Title
**Apoptosis**						
Pdcd7	363082	1.255274323	65.58853688	yellow		programmed cell death 7
Rnf34	282845	1.239231538	94.99706107	green		ring finger protein 34
Tnfrsf1a	25625	0.768312239	-35.3040397	blue		tumor necrosis factor receptor superfamily, member 1a
Tnfrsf12a	302965	0.712387034	-126.5993501	blue		tumor necrosis factor receptor superfamily, member 12a
Traf3ip2	361857	0.674737931	-30.46750417	blue		Traf3 interacting protein 2
**Cell Cycle**						
Ccnd1	58919	0.75910663	-34.85666798	blue		cyclin D1
Cdc14a	310806	0.726344125	-138.4896142	blue		CDC14 cell division cycle 14 homolog A (S. cerevisiae)
Hmg20b	362825	1.206551799	45.84814432	grey		high mobility group 20 B
Klhl13	313445	1.202250811	43.64456126	brown		kelch-like 13 (Drosophila)
Rfpl4a	292583	1.224074208	40.78712222	brown		ret finger protein-like 4A
Wee2	502750	1.228749006	35.72485564	brown		WEE1 homolog 2 (S. pombe)
Ypel4	502643	0.787380069	-20.20313217	blue		yippee-like 4 (Drosophila)
**Cytoskeleton - ECM**						
Ahnak	191572	0.796421876	-88.52096956	blue		AHNAK nucleoprotein
Ankh	114506	0.71801695	-39.27642253	blue		ankylosis, progressive homolog (mouse)
Cdc42ep2	309175	0.725572405	-100.7677734	blue		CDC42 effector protein (Rho GTPase binding) 2
Cntnap1	84008	0.701843836	-44.18179399	blue		contactin associated protein 1
Eml5	444982	1.251603384	89.48839506	brown		echinoderm microtubule associated protein like 5
Emp1	25314	0.733687891	-106.5109003	blue	TNFa	epithelial membrane protein 1
Emp3	81505	0.730565774	-268.7915098	blue		epithelial membrane protein 3
Fbln2	282583	1.205788779	18.36057904	grey	E2	fibulin 2
Fgg	24367	1.360354431	18.07272967	grey		fibrinogen gamma chain
Flrt2	299236	0.516523574	-141.3412014	blue		fibronectin leucine rich transmembrane protein 2
Fndc3b	294925	0.819859926	-91.39665749	blue		fibronectin type III domain containing 3B
Frem2	310418	1.307308589	88.60343993	grey	TNFa	Fras1 related extracellular matrix protein 2
Gsn	296654	0.775063791	-35.83935029	blue		gelsolin
Klhl2	290692	0.829311965	-38.84638107	blue		kelch-like 2, Mayven (Drosophila)
Lgals3	83781	0.790980226	-89.5553613	blue		lectin, galactoside-binding, soluble, 3
Lgals7	29518	0.718802028	-30.78396567	blue		lectin, galactoside-binding, soluble, 7
Lmna	60374	0.789001333	-43.02415723	blue		lamin A
LOC686753	686753	1.427484072	22.64357594	blue		similar to nephronectin isoform a
Mpp7	307035	1.206353332	35.23458359	brown		membrane protein, palmitoylated 7 (MAGUK p55 subfamily member 7)
Mtss1	362918	0.805776413	-85.67123341	blue	ActivinA	metastasis suppressor 1
Ncdn	89791	0.827383041	-37.77365409	blue		neurochondrin
Ninl	311529	0.748008917	-75.56431465	blue		ninein-like
Pfn2	81531	1.261232219	381.5751286	yellow		profilin 2
Plec	64204	0.809048242	-39.76949721	blue		plectin
Plp2	302562	0.823993699	-427.7281061	blue		proteolipid protein 2 (colonic epithelium-enriched)
Pragmin	306506	0.664427269	-29.58043011	blue		pragma of Rnd2
Sdc4	24771	0.62482525	-467.6702646	blue		syndecan 4
Stom	296655	0.662231628	-44.22593288	blue		stomatin
Vcam1	25361	1.221282459	11.91102149	turquoise		vascular cell adhesion molecule 1
Vim	81818	0.787409866	-783.9670981	blue		vimentin
**Development**						
Amot	300289	0.627706764	-78.79204321	blue		angiomotin
Asap2	362719	0.80300327	-130.0429609	blue		ArfGAP with SH3 domain, ankyrin repeat and PH domain 2
Dcdc2	291130	1.261433717	12.63047346	yellow		doublecortin domain containing 2
Fam189a2	309415	0.76158742	-38.1648667	blue		family with sequence similarity 189, member A2
Lhfpl1	300286	0.396077302	-436.6146125	blue		lipoma HMGIC fusion partner-like 1
LOC100362819	100362819	0.831786801	-32.0470247	blue		autism susceptibility candidate 2-like
LOC500331	500331	1.295134537	43.24252612	brown		similar to osteoclast inhibitory lectin
Lrrn1	500280	1.288375577	81.43055555	blue		leucine rich repeat neuronal 1
Pof1b	302328	0.534277581	-40.71938892	blue		premature ovarian failure 1B
Rbl2	81758	1.234028713	114.0286766	yellow		retinoblastoma-like 2
RGD1559891	309028	1.26958985	71.99486535	brown		similar to synaptonemal complex protein 3
Rsad2	65190	1.326561631	15.69198628	turquoise		radical S-adenosyl methionine domain containing 2
Sfrp2	310552	1.322620827	106.1824639	grey		secreted frizzled-related protein 2
Smpx	84416	1.22789965	40.65568578	brown		small muscle protein, X-linked
Sox6	293165	1.212368682	22.35874091	yellow		SRY (sex determining region Y)-box 6
Sytl2	361604	1.257912069	19.81231226	brown		synaptotagmin-like 2

Wbscr17	288611	1.310144873	17.88609537	grey		Williams-Beuren syndrome chromosome region 17 homolog (human)
**Electron Transport**						
Cox6b2	654441	0.78694585	-73.94172948	blue		cytochrome c oxidase subunit VIb polypeptide 2
Pde11a	140928	1.204501685	14.84740299	brown		phosphodiesterase 11A /// cytochrome c, testis
Ucp2	54315	0.762011547	-400.8659214	blue		uncoupling protein 2 (mitochondrial, proton carrier)
**Epigenetics**						
Hist1h1a	291145	0.813937	-87.53396922	blue		histone cluster 1, H1a
Hist1h2bb	679910	0.760778018	-241.4304051	grey		histone cluster 1, H2bb
Hist2h3c2	310678	0.780909578	-232.6116482	blue		histone cluster 2, H3c2
Hist2h4	295277	0.816591755	-83.85823514	brown		histone cluster 2, H4
Kdm3a	312440	1.345272641	222.7763272	brown		lysine (K)-specific demethylase 3A
LOC308670	308670	1.320866362	100.7061063	brown		pink-eyed dilution
LOC679840	679840	0.789773439	-99.17569746	grey		similar to germinal histone H4 gene
Mettl4	316731	1.200621744	20.9280622	brown		methyltransferase like 4
Mettl7a	315306	0.763464411	-54.38134246	blue		methyltransferase like 7A
RGD1566401	500717	0.670779765	-186.8103732	blue		similar to GTL2, imprinted maternally expressed untranslated
**EST's**						
MGC112883	500651	1.254658594	424.1884759	blue		LOC500651
---	---	0.799271297	-45.64036868	blue		---
---	---	0.683191796	-36.82384616	blue		---
---	---	0.814509352	-26.48183912	blue		---
---	---	0.64722598	-98.27464177	blue		---
---	---	0.694334276	-20.96060051	blue		---
---	---	1.207713555	19.99926511	brown		---
---	---	0.768762415	-26.20236276	grey		---
---	---	1.24274902	11.47494328	blue		---
---	---	0.780018941	-136.8468944	grey		---
---	---	0.615798472	-107.2231675	blue		---
---	---	1.205191185	23.21130745	grey		---
---	---	1.285583735	127.339962	grey		---
---	---	0.816111085	-61.74331699	grey		---
---	---	1.237671449	27.18509773	magenta		---
---	---	0.719526182	-86.29120427	blue		---
---	---	0.783804509	-15.41223245	blue		---
---	---	0.784014152	-45.7769842	blue		---
---	---	0.827045283	-215.6147148	magenta		---
---	---	0.751343857	-49.75634254	blue		---
---	---	1.218496878	23.16201427	blue		---
---	---	1.328562897	19.56614674	blue		---
---	---	1.570388238	33.33206542	grey		---
---	---	1.384626047	17.86334563	brown		---
---	---	1.498379547	51.06728925	grey		---
---	---	1.437958379	18.28043339	green		---
---	---	0.763980275	-22.55679537	green		---
---	---	1.376931315	11.75880507	grey		---
---	---	1.258605789	93.88223645	brown		---
---	---	0.693105926	-29.89954593	blue		---
---	---	1.239602611	70.57720127	brown		---
---	---	0.720604115	-103.3391483	blue		---
---	---	0.806348515	-93.33925669	blue		---
---	---	0.812079549	-22.25828456	red		---
---	---	0.812066379	-205.5699975	blue		---
---	---	0.832578367	-38.78337556	magenta		---
---	---	0.807217357	-81.57966386	grey		---
---	---	0.760490495	-48.55378843	grey		---
---	---	1.285542152	21.78499221	turquoise		---
---	---	0.806328846	-26.398178	grey		---
---	---	0.806235124	-34.21566195	blue		---
---	---	0.413147521	-137.0478928	blue		---
---	---	0.795287915	-50.69745515	grey		---
---	---	0.832092116	-17.53395329	blue		---
---	---	1.214975547	16.59469604	blue		---
---	---	0.767576203	-280.2802107	blue		---
---	---	0.75582643	-440.0116511	grey		---
---	---	0.712275775	-614.0072552	blue		---
---	---	0.818017208	-63.57172256	blue		---
---	---	0.725130333	-29.80287421	green		---
**Golgi Apparatus**						
Manba	310864	0.704036606	-76.32931331	blue		mannosidase, beta A, lysosomal
**Growth**						

**Factors**						
Ccl20	29538	0.750210851	-30.10816135	grey		chemokine (C-C motif) ligand 20
C1qtnf1	303701	0.58681573	-68.71952888	blue		C1q and tumor necrosis factor related protein 1
Cxcl16	497942	0.660465431	-390.24059	blue		chemokine (C-X-C motif) ligand 16 /// zinc finger, MYND-type containing 15
Efemp1	305604	1.240552564	55.84182632	brown		EGF-containing fibulin-like extracellular matrix protein 1
Fgf12	170630	0.717291714	-54.94376095	blue		fibroblast growth factor 12
Fst	24373	1.297885599	184.9621526	grey		follistatin
Grb14	58844	1.234157897	44.21001132	brown		growth factor receptor bound protein 14
Inha	24504	1.394846249	109.506321	yellow		inhibin alpha
Ogn	291015	1.22968578	432.6448322	yellow		osteoglycin
Pgf	94203	1.205156673	17.64738199	brown		placental growth factor
Pnoc	25516	0.291149393	-227.6386495	blue		prepronociceptin
Sema3a	29751	0.441937465	-544.0429421	blue	P4	sema domain, immunoglobulin domain (Ig), short basic domain, secreted, (semaphorin) 3A
Sema3d	246262	0.623304122	-35.97591267	blue		sema domain, immunoglobulin domain (Ig), short basic domain, secreted, (semaphorin) 3D
Sema3f	315996	0.798532552	-48.07695148	blue		sema domain, immunoglobulin domain (Ig), short basic domain, secreted, (semaphorin) 3 F
Stc1	81801	0.579155305	-302.3872783	pink	AMH, ActivinA, P4, E2	stanniocalcin 1
Tgfb2	81809	0.611038491	-185.1948286	blue		transforming growth factor, beta 2
**Immune Response**						
C3	24232	1.282543136	80.70886075	grey	E2	complement component 3
C4b	24233	1.521386566	48.7510472	grey	E2	complement component 4B (Chido blood group) /// complement component 4, gene 2
Defb29	641519	0.723557885	-25.6525581	blue		defensin beta 29
F3	25584	0.819441937	-53.42038907	blue		coagulation factor III (thromboplastin, tissue factor)
Hmcn1	289094	0.673901347	-220.6364442	blue	ActivinA	hemicentin 1
Ifitm1	293618	0.716553047	-427.8713082	blue		interferon induced transmembrane protein 1
LOC100364751	100364751	0.795761798	-10.06433143	grey		immunoreceptor Ly49si3-like
Ly6c	56778	0.387964853	-432.3270976	blue	AMH, ActivinA	Ly6-C antigen
Mal2	362911	2.413149028	196.6468875	yellow	AMH	mal, T-cell differentiation protein 2
Nlrp4	499069	1.278378526	50.30292345	brown		NLR family, pyrin domain containing 4
**Metabolism & Transport**						
Adcy6	25289	0.832214299	-25.82744821	blue		adenylate cyclase 6
Ak3l1	29223	1.404859672	134.0140647	yellow	E2	adenylate kinase 3-like 1
Akr1c14	191574	1.416745519	33.23941998	brown		aldo-keto reductase family 1, member C14
Aqp9	65054	0.361843343	-270.0445822	blue	AMH	aquaporin 9
Atp1b1	25650	0.738365297	-425.9729534	blue		ATPase, Na+/K+ transporting, beta 1 polypeptide
Bace2	288227	0.488004743	-272.988624	blue	ActivinA	beta-site APP-cleaving enzyme 2
Btd	306262	0.782800592	-39.91297943	blue		biotinidase
Cadps	26989	0.722066885	-10.51388176	blue		Ca++-dependent secretion activator
Cadps2	312166	1.256808344	53.58940143	brown		Ca++-dependent secretion activator 2
Capn8	170808	0.769365338	-131.5506702	pink	Innhba, P4	calpain 8
Car12	363085	1.279536697	31.09417615	brown	AMH, P4	carbonic anyhydrase 12
Catsperg1	292767	1.207679815	40.81465714	green		cation channel, sperm-associated, gamma 1
Chst11	314694	0.797188364	-65.82596137	blue		carbohydrate (chondroitin 4) sulfotransferase 11
Cnga1	85259	1.971543938	32.42179355	brown		cyclic nucleotide gated channel alpha 1
Crot	83842	0.830211681	-130.9412093	blue		carnitine O-octanoyltransferase
Ctbs	81652	0.81983657	-38.7194417	blue		chitobiase, di-N-acetyl- /// spermatogenesis associated 1
Cyp17a1	25146	1.238173171	13.97476501	blue		cytochrome P450, family 17, subfamily a, polypeptide 1
Dct	290484	1.229588399	26.3458068	grey		dopachrome tautomerase (dopachrome delta-isomerase, tyrosine-related protein 2)
Elovl6	171402	0.722604086	-61.97761682	blue		ELOVL family member 6, elongation of long chain fatty acids (yeast)
Fads1	84575	0.774204092	-73.32414398	blue		fatty acid desaturase 1
Fut8	432392	1.225295325	162.8881997	yellow		fucosyltransferase 8 (alpha (1,6) fucosyltransferase)
Gstp1	24426	0.628517405	-488.929501	blue		glutathione S-transferase pi 1
Hsd17b2	79243	1.368794587	70.64105742	yellow	TNFa	hydroxysteroid (17-beta) dehydrogenase 2
Hsd3b1	360348	1.26017282	78.65833516	yellow	E2	hydroxy-delta-5-steroid dehydrogenase, 3 beta- and steroid delta-isomerase 1 /// 3 beta-hydroxysteroid dehydrogenase/delta-5-delta-4 isomerase type II
Kirrel	310695	0.739490893	-57.13615406	blue		kin of IRRE like (Drosophila)
Loxl2	290350	0.74433126	-25.76031875	blue		lysyl oxidase-like 2
Mgst1	171341	0.658010809	-912.9369489	blue		microsomal glutathione S-transferase 1
Mgst2	295037	0.728826192	-277.2604293	blue		microsomal glutathione S-transferase 2

Oas1a	192281	1.376605249	158.9690398	yellow		2'-5' oligoadenylate synthetase 1A
Oas1d	304508	1.291575808	20.87441738	yellow	AMH	2'-5' oligoadenylate synthetase 1D
Pdha1	29554	1.248015023	50.81220232	brown		pyruvate dehydrogenase (lipoamide) alpha 1 /// similar to mitogen-activated protein kinase kinase kinase 15
Phyhd1	296621	1.435001245	107.7587454	grey	TNFa	phytanoyl-CoA dioxygenase domain containing 1
Prdx6	94167	0.759918131	-207.0107942	blue		peroxiredoxin 6
Retsat	246298	0.730613557	-73.7839636	blue		retinol saturase (all trans retinol 13,14 reductase)
Selm	498398	0.804807904	-23.38979943	blue		selenoprotein M
Sepp1	29360	0.798853369	-241.395099	blue		selenoprotein P, plasma, 1 /// coiled-coil domain containing 152
Slc28a3	140944	1.576808139	60.84092363	yellow	P4	solute carrier family 28 (sodium-coupled nucleoside transporter), member 3
Slc29a1	63997	0.742347866	-44.58204893	blue		solute carrier family 29 (nucleoside transporters), member 1
Slc35f1	502421	1.487732151	56.07452676	yellow		solute carrier family 35, member F1
Slc35f2	300713	0.796361013	-21.68517822	blue		solute carrier family 35, member F2
Slc43a1	311168	0.686457914	-33.49959786	blue		solute carrier family 43, member 1
Slc45a4	315054	0.74684354	-61.22811626	blue		solute carrier family 45, member 4
Slc8a1	29715	0.827164311	-28.40419713	blue		solute carrier family 8 (sodium/calcium exchanger), member 1
Star	25557	0.7528862	-38.49412728	blue		steroidogenic acute regulatory protein
Sult1a1	83783	1.320850038	82.53927557	yellow	P4	sulfotransferase family, cytosolic, 1A, phenol-preferring, member 1
Tpcn1	246215	0.81115442	-23.92515025	blue		two pore segment channel 1
**MicroRNA & RNA Processing**						
Cpeb3	309510	1.215830155	16.43546224	green		cytoplasmic polyadenylation element binding protein 3
Mir23b	100314002	0.640271976	-87.51389963	blue		microRNA mir-23b
**Miscellaneous & Unknown**						
Btn1a1	306956	1.217953241	20.22442408	blue		butyrophilin, subfamily 1, member A1
Ccdc73	499848	1.22669184	25.0355428	brown		coiled-coil domain containing 73
Exd1	311334	1.326083459	78.66352679	brown		exonuclease 3'-5' domain containing 1
Fam129a	63912	0.80986585	-26.63117912	blue	ActivinA	family with sequence similarity 129, member A
Fam132a	313774	1.503555512	181.8692149	grey		family with sequence similarity 132, member A
Fam13a1	362378	1.229978914	32.70695803	brown		family with sequence similarity 13, member A1
Fam176b	362597	0.811266293	-43.78182646	brown	ActivinA	family with sequence similarity 176, member B
Kdelc2	315664	1.261114662	125.8403044	green		KDEL (Lys-Asp-Glu-Leu) containing 2
LOC100359720	100359720	1.290589765	29.11836013	brown	P4	rCG64386-like
LOC100360053	100360053	0.773865128	-45.62256153	blue		histone cluster 1, H2ae-like
LOC100361234	100361234	0.728625443	-57.26646866	blue	CTGF	BTB (POZ) domain containing 19-like
LOC684822	684822	0.822894822	-25.90813483	grey		similar to transcription elongation factor B (SIII), polypeptide 2
Magee2	302392	1.202649428	28.07515408	brown		melanoma antigen, family E, 2
RGD1305184	307412	1.562066611	119.4348789	turquoise		similar to CDNA sequence BC023105
RGD1305899	311715	1.227872308	24.29697468	brown		similar to Protein C20orf158
RGD1307119	303743	1.332798171	17.86308117	grey		hypothetical LOC303743
RGD1308626	361087	1.223402786	19.90383439	green		similar to 9630044O09Rik protein
RGD1563065	289633	1.329796704	70.92055586	yellow		similar to 3110047P20Rik protein
RGD1565493	500853	1.259440464	12.86174106	grey	AMH	similar to DKFZP434I092 protein
Sfxn2	294011	1.206046426	24.38456165	grey		sideroflexin 2
Sh2d4a	306376	1.214022512	29.83832968	brown		SH2 domain containing 4A
Tmem116	690442	1.230373221	20.90460742	grey		transmembrane protein 116
Tmem117	500921	1.211282512	49.48086932	blue		transmembrane protein 117
Tmem164	367763	0.819600065	-52.92524832	blue		transmembrane protein 164
Tmem50b	360698	1.207446382	302.7370058	brown	AMH	transmembrane protein 50B
Trim2	361970	1.344915627	24.86294443	yellow	P4, E2	tripartite motif-containing 2
Wdr35l	503018	1.233688232	47.13515033	brown		WD repeat domain 35-like /// WD repeat domain 35
**Proteolysis**						
Adamts17	293004	1.202961042	37.25172617	yellow	TNFa, E2	ADAM metallopeptidase with thrombospondin type 1 motif, 17
Ctsk	29175	0.69209846	-148.3007293	blue		cathepsin K
Ctsl1	25697	0.722297782	-1204.310545	blue		cathepsin L1
Dpp6	29272	1.535517381	77.5038239	yellow		dipeptidylpeptidase 6
Naaladl2	499583	1.351814348	17.61651012	grey		N-acetylated alpha-linked acidic dipeptidase-like 2
Prss35	315866	1.285467734	86.93617653	grey		protease, serine, 35
Rnf144b	364681	0.815601521	-29.68851659	brown	ActivinA	ring finger protein 144B
Rnf149	363222	1.204755992	77.09091499	brown		ring finger protein 149
**Receptors &**						

**Binding Proteins**						
Amph	60668	0.652406642	-193.9887327	blue		amphiphysin
Axl	308444	0.669476447	-235.4043884	blue	AMH	Axl receptor tyrosine kinase
Epha2	366492	0.701070707	-19.63579657	blue		Eph receptor A2
Epha4	316539	0.77646297	-106.7011316	blue	ActivinA	Eph receptor A4
Ephb2	313633	0.801246842	-46.62327893	blue		Eph receptor B2
Fzd2	64512	0.799321183	-19.01152489	grey		frizzled homolog 2 (Drosophila)
Gabbr1	81657	0.815415895	-24.91221605	blue		gamma-aminobutyric acid (GABA) B receptor 1
Grina	266668	0.558179853	-246.59543	blue		glutamate receptor, ionotropic, N-methyl D-aspartate-associated protein 1 (glutamate binding)
Grpr	24938	0.62364032	-25.91908832	blue		gastrin releasing peptide receptor
Htr1b	25075	1.307506938	56.85367216	grey		5-hydroxytryptamine (serotonin) receptor 1B
Htr2a	29595	1.219528579	113.7612866	yellow	AMH, ActivinA	5-hydroxytryptamine (serotonin) receptor 2A
Igfbp4	360622	0.739540982	-92.45760519	blue		insulin-like growth factor binding protein 4
Igfbp5	25285	1.367820807	577.6935458	grey	TNFa	insulin-like growth factor binding protein 5
Il1r1	25663	0.509107314	-252.2668171	blue		interleukin 1 receptor, type I
Ldlr	300438	0.75064141	-46.10255543	blue		low density lipoprotein receptor
Lpar4	302378	0.744775539	-273.8607396	blue		lysophosphatidic acid receptor 4
Nrp2	81527	0.787836988	-28.77392401	blue		neuropilin 2
Pgrmc1	291948	0.783927905	-153.8101781	blue		progesterone receptor membrane component 1
Ppargc1a	83516	0.424493601	-177.9232143	blue		peroxisome proliferator-activated receptor gamma, coactivator 1 alpha
PVR	25066	0.662539864	-50.02065016	blue		poliovirus receptor
RGD1562717	363767	1.224487033	101.0546147	magenta	CTGF	similar to ABI gene family, member 3 (NESH) binding protein
S100a11	445415	0.795460581	-746.1113136	blue		S100 calcium binding protein A11 (calizzarin)
S100a3	114216	0.423703322	-62.88749185	blue		S100 calcium binding protein A3
S100a6	85247	0.509665891	-429.3313893	blue		S100 calcium binding protein A6
Smoc1	314280	1.302028324	84.26195819	yellow		SPARC related modular calcium binding 1
**Signaling**						
Ano6	315272	0.68960026	-263.1442845	blue		anoctamin 6
Anxa2	56611	0.828488504	-346.7968511	blue		annexin A2
Anxa5	25673	0.662371638	-683.1328392	blue		annexin A5
Baiap2l1	304282	0.816525601	-104.088345	blue		BAI1-associated protein 2-like 1
Dtx3l	498089	1.243972898	36.7923454	yellow	AMH	deltex 3-like (Drosophila)
Dusp4	60587	0.697054364	-18.34214213	blue		dual specificity phosphatase 4
Itga5	315346	0.814269453	-37.60991516	blue		integrin, alpha 5 (fibronectin receptor, alpha polypeptide)
Gng13	685451	1.24556727	11.01675282	yellow	AMH	guanine nucleotide binding protein (G protein), gamma 13
Grid2	79220	0.756001235	-37.29810641	blue		glutamate receptor, ionotropic, delta 2
Hcn1	84390	1.366228103	39.28606879	grey		hyperpolarization-activated cyclic nucleotide-gated potassium channel 1
Lppr4	295401	0.731762996	-36.6785618	blue		lipid phosphate phosphatase-related protein type 4
Map2k6	114495	1.22211354	26.57590364	yellow		mitogen-activated protein kinase kinase 6
Neto2	307757	1.218690838	58.09294689	green		neuropilin (NRP) and tolloid (TLL)-like 2
Phlda3	363989	0.815451744	-16.76692964	blue		pleckstrin homology-like domain, family A, member 3
Plekho2	315764	1.356658112	28.95302665	blue		pleckstrin homology domain containing, family O member 2
Prkcb	25023	0.688167275	-102.5161045	blue		protein kinase C, beta
Ptpn13	498331	0.766360683	-181.0123595	blue		protein tyrosine phosphatase, non-receptor type 13
Ralb	116546	1.231929426	30.1850367	blue		v-ral simian leukemia viral oncogene homolog B (ras related; GTP binding protein)
Scube1	315174	1.511345997	61.1490529	blue		signal peptide, CUB domain, EGF-like 1
Shc4	679845	0.709304932	-15.94972813	blue		SHC (Src homology 2 domain containing) family, member 4
Spred2	305539	0.687450837	-42.22679848	blue		sprouty-related, EVH1 domain containing 2
Spry2	306141	0.821315824	-28.7913127	grey		sprouty homolog 2 (Drosophila)
Stk40	360230	0.809663165	-29.37364526	blue		serine/threonine kinase 40
Syngap1	192117	0.830324842	-22.01321049	blue		synaptic Ras GTPase activating protein 1 homolog (rat)
Tek	89804	0.649109523	-92.39185766	blue	P4	TEK tyrosine kinase, endothelial
Trib1	78969	0.751680664	-20.58117905	blue		tribbles homolog 1 (Drosophila)
Ubash3b	315579	0.325178896	-190.1393843	blue		ubiquitin associated and SH3 domain containing, B
Upk1b	303924	0.62182627	-51.15342518	blue		uroplakin 1B /// rCG52781-like
**Transcription**						
Etv5	303828	0.801457542	-15.26425665	blue		ets variant 5
Mdfic	362325	1.230229618	55.779862	brown	ActivinA	MyoD family inhibitor domain containing
Mia	81510	1.337115157	33.55128275	yellow		melanoma inhibitory activity
Ndrg1	299923	1.223111026	39.87697397	blue		N-myc downstream regulated gene 1
Prr13	363004	0.780955264	-367.3470183	blue		proline rich 13
Noto	502857	1.218598404	12.44430602	grey		notochord homeobox
Rai14	294804	0.75034542	-100.5449016	blue		retinoic acid induced 14
Rhox8	503423	1.400773359	36.34205418	blue		reproductive homeobox 8

Shisa5	301013	0.748566871	-212.8171144	blue		shisa homolog 5 (Xenopus laevis)
Tceanc	367782	1.276389296	59.47979954	brown		transcription elongation factor A (SII) N-terminal and central domain containing
Trerf1	316219	0.788036014	-58.23676895	blue		transcriptional regulating factor 1
Zfp192	306974	1.242479318	41.57474484	brown		zinc finger protein 192
**Translation & Protein Modification**						
Arl4a	29308	1.218434067	40.35473296	brown		ADP-ribosylation factor-like 4A
Parp9	303905	1.230307778	124.5385557	yellow		poly (ADP-ribose) polymerase family, member 9
Parp11	500323	1.228936419	47.0331845	brown		poly (ADP-ribose) polymerase family, member 11
Psen2	81751	0.808871961	-32.80423076	blue		presenilin 2
Rbm47	305340	0.76227625	-27.12619072	blue		RNA binding motif protein 47

D. Genes influenced by treatment with ActivinA						
Gene Symbol	Entrez ID	Fold Change	Mean Diff	Module	Present in Other Lists	Gene Title
Apoptosis						
Ddx58	297989	1.545465093	38.21850204	turquoise		DEAD (Asp-Glu-Ala-Asp) box polypeptide 58
Tnfrsf11b	25341	0.685885224	-73.83015515	turquoise		tumor necrosis factor receptor superfamily, member 11b
Cell Cycle						
Cenpq	363198	0.817061328	-19.09851113	brown		centromere protein Q /// glycine-N-acyltransferase-like 3
Hmgb3	305373	0.815899757	-68.64647412	grey		high mobility group box 3
Ndel1	170845	0.773447252	-79.76753196	brown		nuclear distribution gene E-like homolog 1 (A. nidulans)
Uhmk1	246332	0.78289535	-55.10075998	brown		U2AF homology motif (UHM) kinase 1
Cytoskeleton-ECM						
Bcam	78958	1.45095992	137.0363368	turquoise		basal cell adhesion molecule
Cldn15	304388	1.395275844	67.78739549	pink		claudin 15
Cldn6	287098	0.778376164	-24.19722351	turquoise		claudin 6
Fn1	25661	0.572959892	-239.0387405	turquoise	P4	fibronectin 1
Krt7	300242	0.768455767	-49.64962116	turquoise		keratin 7
Lgals9	25476	1.319189478	92.48588531	turquoise	P4	lectin, galactoside-binding, soluble, 9
Mtss1	362918	1.253629205	71.90809091	blue	FGF2	metastasis suppressor 1
Muc16	315451	1.432438213	141.4747554	yellow	AMH	mucin 16, cell surface associated
Parvb	362973	1.259458286	39.00661027	blue		parvin, beta
Pdlim3	114108	0.547213819	-47.34136447	turquoise		PDZ and LIM domain 3
Shroom3	305230	1.298438509	55.39610254	turquoise		shroom family member 3
Sorbs2	114901	1.216733555	133.6509798	turquoise	AMH, TNFa	sorbin and SH3 domain containing 2
Tspan4	293627	1.305369363	56.03982587	turquoise		tetraspanin 4 /// polymerase (RNA) II (DNA directed) polypeptide L
Tubb2b	291081	0.793362319	-21.42145664	turquoise		tubulin, beta 2b
Tubb2c	296554	1.242189049	113.9693318	turquoise		tubulin, beta 2c
Development						
Cryab	25420	1.334934145	25.54536046	pink	P4	crystallin, alpha B
Dbn1	81653	0.81147308	-61.7069265	turquoise		drebrin 1
Fry	304244	0.799506889	-13.81839128	turquoise		furry homolog (Drosophila)
Hoxc5	315341	1.350810929	49.26737028	turquoise		homeo box C5
Lrrn4	311443	1.343876969	185.2152917	turquoise		leucine rich repeat neuronal 4
Ndnl2	309259	0.818937467	-15.3168142	brown		necdin-like 2
Nrep	338475	0.715438426	-273.5641442	brown		neuronal regeneration related protein
Spin2a	317395	0.75165961	-41.87049072	brown		spindlin family, member 2A
DNA Repair						
Mdc1	309595	1.243679666	28.51309366	green		mediator of DNA damage checkpoint 1
Electron Transport						
Atp5s	362749	0.74892349	-31.98022651	brown		ATP synthase, H+ transporting, mitochondrial F0 complex, subunit s (factor B)
Cyb5r1	304805	1.460898247	58.96485171	turquoise		cytochrome b5 reductase 1
Ndufa13	314759	1.285467536	55.79399382	turquoise		NADH dehydrogenase (ubiquinone) 1 alpha subcomplex, 13
Ndufb10	681418	1.20195944	81.15408178	turquoise		NADH dehydrogenase (ubiquinone) 1 beta subcomplex, 10
Epigenetics						
Cav1	25404	1.276365399	330.8843785	blue		caveolin 1, caveolae protein
Hist1h2ail	291159	1.260891376	228.8617092	turquoise		histone cluster 1, H2ai-like
Hist1h2bf	306969	1.284777793	45.88893347	grey	TNFa	histone cluster 1, H2bf
Hist1h2bm	361247	1.227141681	46.58477544	brown		histone cluster 1, H2bm
Set	307947	0.788776316	-219.3421002	turquoise		SET nuclear oncogene
Trrap	288471	1.203031918	55.11088527	turquoise		transformation/transcription domain-associated protein
EST's						
LOC498465	498465	1.223595566	28.0832215	red		similar to RIKEN cDNA 1700001F09 /// similar to Spetex-2C protein /// similar to Spetex-2C protein /// similar to RIKEN cDNA 1700001F09
RGD1306917	361805	1.257014783	88.2129961	turquoise		similar to RIKEN cDNA 2900010M23 /// similar to RIKEN cDNA 2900010M23
---	---	1.274505302	73.35879968	turquoise		---
---	---	1.363405163	198.7045535	turquoise		---
---	---	0.783299521	-17.92117375	grey		---
---	---	0.816747889	-34.19327692	pink		---
---	---	1.355779324	34.89051776	red		---
---	---	1.315016083	280.8276842	magenta		---

---	---	1.242422956	18.11334132	turquoise		---
---	---	1.213970213	35.06706955	red		---
---	---	0.818650054	-99.44589404	turquoise		---
---	---	1.309141031	180.8626551	turquoise		---
---	---	0.800739234	-116.2346066	grey		---
---	---	0.785270505	-33.54153893	turquoise		---
---	---	1.257001207	24.14329445	grey		---
---	---	1.244417611	59.17388617	red		---
---	---	1.315770267	61.27708368	blue		---
---	---	0.746695391	-39.06952106	turquoise		---
---	---	1.293824413	33.16873144	blue		---
---	---	0.830246989	-99.4906392	turquoise		---
---	---	0.803303068	-466.0056334	grey		---
---	---	1.359002304	31.70613511	red		---
---	---	1.383519326	55.02011918	blue		---
---	---	1.555358968	250.381379	red		---
---	---	0.806631402	-23.11378927	turquoise		---
---	---	1.428729268	26.38215045	pink		---
---	---	1.202637791	43.4998685	blue		---
---	---	1.319586789	28.16262023	blue		---
---	---	1.284977212	51.6428924	turquoise		---
---	---	0.795190265	-25.88689027	red		---
---	---	1.287875999	117.6693744	turquoise		---
---	---	1.288294215	25.09521501	grey		---
---	---	1.235045754	12.25650176	turquoise		---
---	---	1.424862455	235.8995112	turquoise		---
---	---	1.257095297	99.29639265	yellow		---
---	---	0.772909917	-19.56201661	grey		---
---	---	1.322441843	58.37409907	turquoise		---
---	---	1.240022183	11.58664695	turquoise		---
---	---	0.776374127	-169.5331031	grey		---
---	---	1.281625357	75.18638444	turquoise		---
---	---	0.827129577	-16.2949892	turquoise		---
---	---	0.728667432	-31.23090972	turquoise		---
---	---	0.772069403	-28.95474785	turquoise		---
---	---	1.57133123	105.7052162	turquoise		---
---	---	1.229298008	47.9457072	turquoise		---
**Golgi Apparatus**						
Gcnt2	306860	1.559465222	48.92426353	pink		glucosaminyl (N-acetyl) transferase 2, I-branching enzyme
Large	361368	1.208659036	45.5676084	turquoise	TNFa	like-glycosyltransferase
Plbd2	246120	1.232196713	36.88451507	turquoise		phospholipase B domain containing 2
St3gal1	362924	1.921657129	294.6406489	pink		ST3 beta-galactoside alpha-2,3-sialyltransferase 1
St8sia4	116696	1.245882648	41.71289601	pink		ST8 alpha-N-acetyl-neuraminide alpha-2,8-sialyltransferase 4 /// similar to CMP-N-acetylneuraminate-poly-alpha-2,8-sialyltransferase (Alpha-2,8-sialyltransferase 8D) (ST8Sia IV) (Polysialyltransferase-1)
Uap1	498272	0.814530364	-38.66648998	brown		UDP-N-acteylglucosamine pyrophosphorylase 1
**Growth Factors**						
Bmp7	85272	1.257556333	56.98516679	turquoise		bone morphogenetic protein 7
Ccl11	29397	2.395916094	315.4322755	yellow		chemokine (C-C motif) ligand 11
Ctgf	64032	0.830197677	-11.54765652	turquoise		connective tissue growth factor
Cxcl10	245920	1.916582791	256.1948254	turquoise		chemokine (C-X-C motif) ligand 10
Cxcl6	60665	1.30972391	131.6079194	turquoise		chemokine (C-X-C motif) ligand 6 (granulocyte chemotactic protein 2)
Sema6d	311384	1.298766289	342.9162587	turquoise		sema domain, transmembrane domain (TM), and cytoplasmic domain, (semaphorin) 6D
Stc1	81801	1.983388204	206.3260034	pink	FGF2, AMH, P4, E2	stanniocalcin 1
**Immune Response**						
Cd320	362851	1.244908431	91.26535612	turquoise	P4	CD320 molecule
Fcgr2b	289211	0.716441396	-15.181661	turquoise		Fc fragment of IgG, low affinity IIb, receptor (CD32) /// Fc fragment of IgG, low affinity IIa, receptor (CD32)
Hmcn1	289094	1.266359745	95.90385467	blue		hemicentin 1
Ly6c	56778	1.926914216	131.8273857	blue	FGF2, AMH	Ly6-C antigen
Mx1	24575	1.511027718	27.163822	turquoise	P4	myxovirus (influenza virus) resistance 1
Mx2	286918	2.509608746	245.7346095	turquoise	P4	myxovirus (influenza virus) resistance 2
Rorc	368158	1.32575947	85.66731216	turquoise		RAR-related orphan receptor C /// leucine rich repeat and

						Ig domain containing 4
**Metabolism & Transport**						
Acsm5	361637	1.232784719	19.66378589	yellow	P4	acyl-CoA synthetase medium-chain family member 5
Aldoa	24189	1.317386763	299.6714692	turquoise		aldolase A, fructose-bisphosphate
Anpep	81641	1.674548404	205.2177497	turquoise	TNFa	alanyl (membrane) aminopeptidase
Arsb	25227	1.202076941	107.1570232	turquoise		arylsulfatase B
Atp6v0a4	296981	1.311914956	40.3985253	pink	AMH, P4, E2	ATPase, H+ transporting, lysosomal V0 subunit A4
Bace2	288227	1.246901483	51.52217784	blue	FGF2	beta-site APP-cleaving enzyme 2
Bckdk	29603	1.253150209	107.7659839	turquoise		branched chain ketoacid dehydrogenase kinase
Capn8	170808	1.480015362	142.3278919	pink	FGF2, P4	calpain 8
Cat	24248	1.225655454	130.180181	turquoise		catalase
Cbr1	29224	1.237414405	192.682038	turquoise		carbonyl reductase 1 /// inducible carbonyl reductase-like
Dhcr24	298298	1.273545768	38.68207415	turquoise		24-dehydrocholesterol reductase
Edem2	296304	1.350276374	51.08953806	turquoise		ER degradation enhancer, mannosidase alpha-like 2
Enpep	64017	1.52931137	278.7936025	turquoise		glutamyl aminopeptidase
Extl3	56819	1.372104504	225.2743408	turquoise		exostoses (multiple)-like 3
Galm	313843	1.346877122	109.6443372	turquoise		galactose mutarotase (aldose 1-epimerase)
Gstm5	64352	1.273156633	213.4880931	turquoise		glutathione S-transferase, mu 5
Hsd17b10	63864	1.212975867	186.6761415	turquoise		hydroxysteroid (17-beta) dehydrogenase 10
Lox	24914	0.7484832	-81.62660729	turquoise		lysyl oxidase
Me1	24552	0.821279631	-294.8476884	brown		malic enzyme 1, NADP(+)-dependent, cytosolic
Mgst3	289197	1.317830941	104.1055483	turquoise		microsomal glutathione S-transferase 3
Ndst2	114002	1.244458504	39.68599593	turquoise		N-deacetylase/N-sulfotransferase (heparan glucosaminyl) 2
Nme6	58964	1.226962132	43.16882544	grey		non-metastatic cells 6, protein expressed in (nucleoside-diphosphate kinase)
Oas1b	246268	1.510598197	23.79266765	turquoise		2-5 oligoadenylate synthetase 1B
Oas1i	304507	1.254069925	32.38618647	turquoise		2 ' -5 ' oligoadenylate synthetase 1I
Oasl2	304549	1.742054999	81.0957709	turquoise		2'-5' oligoadenylate synthetase-like 2
Pafah1b3	114113	1.210579803	68.49252732	turquoise		platelet-activating factor acetylhydrolase, isoform 1b, subunit 3
Rpgr	367733	0.737767623	-86.65242012	grey		retinitis pigmentosa GTPase regulator
Sec14l1	360668	1.212928825	43.19712329	turquoise		SEC14-like 1 (S. cerevisiae)
Sepw1	25545	1.216389548	21.75627203	turquoise		selenoprotein W, 1
Slc37a2	500973	1.408831512	89.9951506	blue		solute carrier family 37 (glycerol-3-phosphate transporter), member 2
Slc4a4	84484	1.247440487	153.8070358	turquoise		solute carrier family 4 (anion exchanger), member 4
Tomm6	681123	1.218255467	95.06517009	turquoise		translocase of outer mitochondrial membrane 6 homolog (yeast)
Tspo	24230	1.447182064	27.56448294	brown		translocator protein
Tst	25274	1.394463136	90.53544519	brown		thiosulfate sulfurtransferase
**MicroRNA & RNA Processing**						
Mov10	310756	1.376341696	44.17781671	turquoise		Moloney leukemia virus 10
Trmt1	288914	1.223649593	27.91083651	turquoise		TRM1 tRNA methyltransferase 1 homolog (S. cerevisiae)
**Miscellaneous & Unknown**						
Fam129a	63912	1.303222929	26.39287365	blue	FGF2	family with sequence similarity 129, member A
Fam176b	362597	1.242790411	36.76558453	brown	FGF2	family with sequence similarity 176, member B
Fam3c	312159	0.816856931	-119.4465725	turquoise		family with sequence similarity 3, member C
Fam49a	298890	0.814998796	-32.1646697	brown		family with sequence similarity 49, member A
LOC300308	300308	1.277664934	18.59626946	red		similar to hypothetical protein 4930509O22 /// similar to hypothetical protein 4930509O22
LOC501427	501427	1.208694368	30.81027463	red		similar to Spetex-2F protein /// similar to Spetex-2C protein /// similar to Spetex-2C protein /// similar to RIKEN cDNA 1700001F09
LOC679578	679578	0.804526177	-25.36019079	turquoise		similar to Protein C18orf1
LOC685617	685617	1.200109246	24.29701258	grey		similar to spermatogenesis associated glutamate (E)-rich protein 4b /// similar to spermatogenesis associated glutamate (E)-rich protein 4b
LOC685872	685872	1.29594056	26.98763859	red		similar to Spetex-2C protein /// similar to Spetex-2C protein /// similar to Spetex-2C protein
LOC685904	685904	1.333872313	30.13960652	red		similar to Spetex-2F protein /// similar to Spetex-2C protein /// similar to Spetex-2C protein
Lsmd1	287429	1.376781267	117.5609397	turquoise		LSM domain containing 1
Nacc1	171454	1.355200956	169.8909281	turquoise		nucleus accumbens associated 1, BEN and BTB (POZ) domain containing
Nap5	363974	0.826572803	-27.16663377	brown		Nck-associated protein 5
RGD1309540	295930	1.38649204	74.44823755	blue		similar to hypothetical protein MGC40841; similar to hypothetical protein MGC4707

RGD1311849	313346	1.213397261	41.18115872	turquoise		similar to mKIAA1797 protein
RGD1564742	503128	1.249308758	19.97891283	red		similar to hypothetical protein 4930509O22
RGD1565927	304020	0.659855671	-28.59668779	turquoise		similar to 4631422O05Rik protein
Thap4	363291	1.25214096	15.77117967	turquoise		THAP domain containing 4
Tmem132c	363915	1.338910605	40.06198832	turquoise		transmembrane protein 132C
Tmem229b	503035	1.436818862	18.01090042	blue		transmembrane protein 229B
Trim28	116698	1.287561529	158.7375339	turquoise		tripartite motif-containing 28
**Proteolysis**						
Cpa2	296959	1.319385746	161.3547662	blue		carboxypeptidase A2 (pancreatic)
Cpa4	502736	1.329445919	17.65483016	blue		carboxypeptidase A4
Mcpt1	29265	1.456912726	65.81483136	turquoise		mast cell protease 1
Mmp13	171052	0.163947368	-195.7595652	turquoise	TNFa	matrix metallopeptidase 13
Pgpep1	290648	0.826584805	-24.37581108	grey		pyroglutamyl-peptidase I
Prss23	308807	0.826034616	-177.4870107	blue		protease, serine, 23
Rnf144b	364681	1.24649877	25.96761675	brown	FGF2	ring finger protein 144B
Rnf213	303735	1.327924186	153.015911	turquoise		ring finger protein 213
Senp17	408216	1.236494287	10.94501925	red		Sumo1/sentrin/SMT3 specific peptidase 17 /// Sumo1/sentrin/SMT3 specific peptidase 18
Serpinb8	288937	0.705062647	-13.88429885	turquoise		serpin peptidase inhibitor, clade B (ovalbumin), member 8
Usp18	312688	1.974072983	79.88296731	turquoise		ubiquitin specific peptidase 18
**Receptors & Binding Proteins**						
Amhr2	29530	1.363449003	123.134241	turquoise		anti-Mullerian hormone receptor, type II
Epha4	316539	1.28326455	81.81187153	blue	FGF2	Eph receptor A4
Ephb6	312275	1.216613538	21.7255384	turquoise		Eph receptor B6
Htr2a	29595	1.297460325	144.8873178	yellow	FGF2, AMH	5-hydroxytryptamine (serotonin) receptor 2A
Igfbp6	25641	1.201830842	26.46995848	pink		insulin-like growth factor binding protein 6
Olr1619	361033	0.820202513	-12.57599518	grey	AMH	olfactory receptor 1619
Lrp2	29216	1.511862287	61.80599925	pink	AMH, P4	low density lipoprotein-related protein 2
Ltbp2	59106	0.721674863	-16.73159805	turquoise		latent transforming growth factor beta binding protein 2
Olr769	296022	1.216920189	16.86093142	grey		olfactory receptor 769
Ptk7	301242	1.308833575	33.13760243	turquoise		PTK7 protein tyrosine kinase 7
Rtp4	360733	1.444111783	44.49019318	turquoise	P4	receptor (chemosensory) transporter protein 4
Selenbp1	140927	1.280593984	38.07152574	turquoise		selenium binding protein 1
Tgfbr3	29610	1.267975791	68.09068253	blue		transforming growth factor, beta receptor III
Thra	81812	1.236117526	50.41262886	turquoise		thyroid hormone receptor alpha /// nuclear receptor subfamily 1, group D, member 1
Vom2r16	690691	0.777576145	-59.86345701	grey	AMH	vomeronasal 2 receptor, 16
**Signaling**						
Acpp	56780	1.327954856	28.09278453	blue		acid phosphatase, prostate
Agrn	25592	1.256833649	56.2579496	turquoise		agrin
Arhgap26	307459	1.237283595	128.8449108	turquoise		Rho GTPase activating protein 26
Clcn2	29232	1.330822741	23.96542565	turquoise		chloride channel 2
Ehd2	361512	1.416971828	45.50585187	turquoise		EH-domain containing 2
Epb4.1l4a	307514	1.228831736	55.96131847	blue		erythrocyte protein band 4.1-like 4a
Grlf1	306400	1.213350682	80.3974742	turquoise		glucocorticoid receptor DNA binding factor 1
Ifi27	170512	1.364873828	145.3360832	turquoise		interferon, alpha-inducible protein 27
Inpp5a	365382	1.247044287	65.74919003	turquoise	TNFa	inositol polyphosphate-5-phosphatase A
Isg15	298693	1.676397801	45.79519533	turquoise		ISG15 ubiquitin-like modifier
Lgr5	299802	1.236022484	55.17314268	turquoise		leucine rich repeat containing G protein coupled receptor 5
Lynx1	300018	1.402385375	56.98943906	pink		Ly6/neurotoxin 1
Mapkapk3	315994	1.322479647	76.62326984	turquoise		mitogen-activated protein kinase-activated protein kinase 3
Pak1ip1	361232	0.826617736	-63.86296608	brown		PAK1 interacting protein 1
Phlda1	29380	1.241546349	84.92798128	turquoise		pleckstrin homology-like domain, family A, member 1
Rab6b	363123	1.349637942	32.59862682	pink	AMH	RAB6B, member RAS oncogene family
Rhbdl2	298512	0.605252619	-24.86661491	turquoise		rhomboid, veinlet-like 2 (Drosophila)
Rhob	64373	0.724815159	-135.4755093	brown		ras homolog gene family, member B
Rraga	117044	0.819800914	-101.5385671	turquoise		Ras-related GTP binding A
Smpd1	308909	1.498380184	147.3631388	turquoise		sphingomyelin phosphodiesterase 1, acid lysosomal
Snx33	315696	1.219046182	21.37574893	turquoise		sorting nexin 33
Taok2	64666	1.20316034	46.94026744	turquoise		TAO kinase 2
Vrk3	361565	1.331502963	48.78332782	turquoise		vaccinia related kinase 3
**Transcription**						
Etv6	312777	1.314752172	28.29379658	blue		ets variant 6
Fam134b	619558	0.799568137	-22.7823152	turquoise		family with sequence similarity 134, member B
Gata4	54254	1.338363543	161.5569478	turquoise		GATA binding protein 4
Gatad2a	290669	1.217045287	95.32041129	turquoise	TNFa	GATA zinc finger domain containing 2A
Gtf2ird1	246770	0.82946527	-26.95657454	turquoise		GTF2I repeat domain containing 1
Lhx9	289048	1.256551136	237.0674257	turquoise		LIM homeobox 9

LOC100362634	100362634	1.214324735	46.75011474	turquoise		myeloid/lymphoid or mixed-lineage leukemia 2 /// myeloid/lymphoid or mixed-lineage leukemia 2
Mdfic	362325	0.775155421	-86.456182	brown	FGF2	MyoD family inhibitor domain containing
Mybbp1a	60571	1.233618961	67.21865578	green		MYB binding protein (P160) 1a
Polr2a	363633	1.202726369	111.5329218	turquoise		polymerase (RNA) II (DNA directed) polypeptide A
Rag1ap1	295245	1.205123138	146.7066164	blue		recombination activating gene 1 activating protein 1
Stat1	25124	1.209670601	46.58118228	turquoise	TNFa	signal transducer and activator of transcription 1 /// signal transducer and activator of transcription 4
Zfp37	115768	0.77903308	-27.62324153	turquoise		zinc finger protein 37
**Translation & Protein Modification**						
Bat2	294250	1.336869893	75.10488935	turquoise		HLA-B associated transcript 2
Ctdsp1	363249	1.216272757	22.26865256	turquoise		CTD (carboxy-terminal domain, RNA polymerase II, polypeptide A) small phosphatase 1
Ece1	94204	1.222770802	44.5037609	turquoise		endothelin converting enzyme 1
Eif2c1	313594	1.252102484	40.44334703	turquoise		eukaryotic translation initiation factor 2C, 1
Herc6	362376	1.540383063	87.43164293	turquoise		hect domain and RLD 6
LOC499782	499782	1.202116941	796.6917056	turquoise		similar to 60S ribosomal protein L12
Mrpl10	691075	1.237496106	43.24311262	turquoise		mitochondrial ribosomal protein L10
Ncl	25135	0.761603923	-693.8256688	turquoise		nucleolin
Rbm11	288321	0.819511821	-13.72751317	turquoise		RNA binding motif protein 11
RGD1561195	298126	1.371909592	17.13161507	turquoise		similar to ribosomal protein L31
RGD1565415	500123	1.302922631	190.4124439	turquoise		similar to ribosomal protein L27a
Rpl10a	81729	1.274390295	449.9054158	turquoise		ribosomal protein L10A /// similar to ribosomal protein L10a /// similar to ribosomal protein L10a
Rpl13	81765	1.217093352	286.8936754	turquoise		ribosomal protein L13 /// similar to 60S ribosomal protein L13
Rpl29	29283	1.30962087	618.874947	turquoise		ribosomal protein L29 /// similar to 60S ribosomal protein L29 (P23) /// similar to 60S ribosomal protein L29 (P23)
Rpl31	64298	1.256156844	186.5479717	turquoise		ribosomal protein L31
Rpl36	58927	1.21513615	291.152876	turquoise		ribosomal protein L36 /// ribosomal protein L36-like /// ribosomal protein L36-like /// ribosomal protein L36-like
Rpl7a	296596	1.282110936	345.9750197	brown		ribosomal protein L7a /// similar to Rpl7a protein /// similar to 60S ribosomal protein L7a /// similar to 60S ribosomal protein L7a
Rps14	29284	1.236521931	254.9861287	turquoise		ribosomal protein S14
Rps15	29285	1.257283867	250.6397425	turquoise		ribosomal protein S15
Rps19	29287	1.286875814	543.0346539	turquoise		ribosomal protein S19 /// ribosomal protein S19-like /// ribosomal protein S19-like
Rps3	140654	1.225023145	755.3078934	turquoise		ribosomal protein S3
Ssbp3	84354	1.22425925	163.7972713	turquoise		single stranded DNA binding protein 3
Ssbp4	364534	1.254886486	43.14264786	turquoise		single stranded DNA binding protein 4

E. Genes influenced by treatment with P4						
**Gene Symbol**	**Entrez ID**	**Fold Change**	**Mean Diff**	**Module**	**Present in Other Lists**	**Gene Title**
**Apoptosis**						
Birc2	60371	0.798625252	-55.92096666	yellow		baculoviral IAP repeat-containing 2
Dhx37	288647	0.794578737	-16.20648251	grey		DEAH (Asp-Glu-Ala-His) box polypeptide 37
**Cell Cycle**						
Ddit4	140942	0.830409973	-28.70689055	grey		DNA-damage-inducible transcript 4
**Cytoskeleton-ECM**						
Fbln1	315191	1.235599573	72.46044804	black		fibulin 1
Fn1	25661	1.435308214	97.26924366	turquoise	ActivinA	fibronectin 1
Fndc3c1	317224	0.806771901	-17.5552568	grey		fibronectin type III domain containing 3C1
Gpm6a	306439	0.816995062	-100.9556048	grey		glycoprotein m6a
Lgals9	25476	1.281940634	84.06666699	turquoise	ActivinA	lectin, galactoside-binding, soluble, 9
Llgl1	54265	0.829183691	-13.4600042	grey		lethal giant larvae homolog 1 (Drosophila)
LOC689770	689770	1.291099244	19.0813132	grey		similar to osteoclast inhibitory lectin
Lysmd3	315923	0.792022745	-21.69759735	brown		LysM, putative peptidoglycan-binding, domain containing 3
Sync	362606	1.533021164	48.40619477	grey		syncoilin
Tagln	25123	1.434808261	45.39142569	turquoise		transgelin
Tnnc1	290561	1.352133163	92.18175074	grey		troponin C type 1 (slow)
**Development**						
Cryab	25420	0.819779568	-22.38305639	pink	ActivinA	crystallin, alpha B
Hoxd9	688999	0.782093759	-27.42215477	grey		homeo box D9
Dpt	289178	1.413183746	215.6759386	grey		dermatopontin
Phex	25512	1.376704608	40.6216909	grey		phosphate regulating endopeptidase homolog, X-linked
Pkhd1l1	314917	0.757096192	-38.33070633	yellow	E2	polycystic kidney and hepatic disease 1-like 1
Spata7	192225	0.833325342	-94.95862888	brown		spermatogenesis associated 7
**EST's**						
---	---	0.761200444	-31.30395123	grey		---
---	---	0.789979062	-35.05748924	red		---
---	---	0.809808371	-23.89430616	turquoise		---
---	---	1.420931401	113.7911398	grey		---
---	---	0.777742284	-166.5414809	grey		---
---	---	1.214813601	12.35585151	grey		---
---	---	0.746600812	-40.71033231	brown		---
---	---	0.794293144	-22.02304156	grey		---
---	---	0.828443812	-26.67753468	grey		---
---	---	0.833089659	-26.77576109	grey		---
---	---	0.821377814	-32.53249211	brown		---
---	---	1.288032621	44.04136124	brown		---
---	---	0.75918491	-57.43906185	grey		---
---	---	0.805953565	-22.23086513	magenta		---
---	---	1.224326327	10.97711815	brown		---
---	---	0.715165106	-25.4488469	brown		---
---	---	0.819268192	-59.45438663	brown		---
---	---	0.831531591	-67.19247717	brown		---
---	---	0.740539903	-37.58327618	grey		---
---	---	1.222622233	15.67952738	brown		---
---	---	0.788111086	-21.44004219	brown		---
---	---	1.225986364	21.937091	turquoise		---
---	---	0.766290802	-46.82595271	grey		---
---	---	1.398351528	23.41475532	grey		---
---	---	0.811150216	-25.41970895	grey		---
---	---	1.244682248	32.88694566	brown		---
---	---	1.246989518	22.69508283	red		---
---	---	0.787904999	-10.25181038	grey		---
---	---	1.257041701	21.30615433	brown		---
---	---	0.832265969	-12.96031788	brown		---
---	---	1.21775179	192.056277	red		---
---	---	1.21775179	192.056277	red		---
---	---	0.802207527	-131.723543	grey		---
---	---	1.345676023	20.2973372	brown		---
---	---	1.242370588	47.31681637	turquoise		---
---	---	1.225107356	19.95684756	grey		---
---	---	1.322007139	11.60697583	brown		---
---	---	1.246294844	26.44216909	brown		---
---	---	0.831974333	-236.7576139	magenta		---

---	---	1.237393375	45.32775668	grey		---
---	---	0.802190787	-32.65457938	grey		---
---	---	1.352196177	236.8356208	brown		---
---	---	1.223142913	11.82921809	grey		---
---	---	0.765331585	-61.55021792	grey		---
---	---	0.806231409	-21.13005706	pink		---
**Golgi Apparatus**						
B3gnt2	305571	0.789362641	-85.95735195	brown		UDP-GlcNAc:betaGal beta-1,3-N-acetylglucosaminyltransferase 2
**Growth Factors**						
Angptl2	171100	1.241266013	59.22469829	blue		angiopoietin-like 2
Bmp3	25667	1.330362631	19.90551929	grey		bone morphogenetic protein 3
C1qtnf6	315114	1.200322813	29.38671873	grey		C1q and tumor necrosis factor related protein 6
Il1b	24494	1.258007723	11.98166266	black		interleukin 1 beta
Sema3a	29751	0.80648803	-103.3763137	blue	FGF2	sema domain, immunoglobulin domain (Ig), short basic domain, secreted, (semaphorin) 3A
Stc1	81801	0.68849034	-188.2826771	pink	FGF2, ActivinA, AMH, E2	stanniocalcin 1
**Immune Response**						
B2m	24223	1.205762184	986.2381239	grey		beta-2 microglobulin
Cd320	362851	0.816899996	-103.9822196	turquoise	ActivinA	CD320 molecule
Defb21	641636	1.207920542	14.37511247	brown		defensin beta 21
Igha	314487	1.300876443	15.02540095	grey		immunoglobulin heavy chain, alpha /// rCG64227-like
Mx1	24575	1.503452073	26.89598139	turquoise	ActivinA	myxovirus (influenza virus) resistance 1
Mx2	286918	1.802322747	181.854681	turquoise	ActivinA	myxovirus (influenza virus) resistance 2
Rfx5	310659	0.832634713	-36.06352593	grey		regulatory factor X, 5 (influences HLA class II expression)
RT1-CE14	414270	0.823186983	-11.42415719	grey		RT1 class I, locus CE14 /// RT1 class I, locus CE13
**Metabolism & Transport**						
Acsbg1	171410	0.829733861	-75.34557128	turquoise		acyl-CoA synthetase bubblegum family member 1
Acsm5	361637	0.828896807	-21.49599526	yellow	ActivinA	acyl-CoA synthetase medium-chain family member 5
Appbp2	303396	0.750949477	-377.4342951	brown		amyloid beta precursor protein (cytoplasmic tail) binding protein 2
Atp6v0a4	296981	0.741936219	-59.10108828	pink	ActivinA, AMH, E2	ATPase, H+ transporting, lysosomal V0 subunit A4
Capn8	170808	0.786478587	-119.1394625	pink	FGF2, ActivinA	calpain 8
Car12	363085	0.716254504	-56.38383123	brown	FGF2, AMH	carbonic anyhydrase 12
Chst15	286974	0.802525558	-25.6032235	grey		carbohydrate (N-acetylgalactosamine 4-sulfate 6-O) sulfotransferase 15
Cyp2e1	25086	0.780159812	-54.29237161	brown		cytochrome P450, family 2, subfamily e, polypeptide 1
Cyp4f6	266689	1.202824923	25.89822006	grey		cytochrome P450, family 4, subfamily f, polypeptide 6
Ephx1	25315	0.833202544	-76.32442405	grey		epoxide hydrolase 1, microsomal
Gstm2	24424	0.829538315	-289.1498104	turquoise		glutathione S-transferase mu 2
Gstm7	81869	0.747739841	-226.2644812	turquoise		glutathione S-transferase, mu 7
Gsto2	309465	0.795480559	-19.48361008	grey		glutathione S-transferase omega 2
Hsd11b1	25116	1.209417342	26.22919784	blue		hydroxysteroid 11-beta dehydrogenase 1
Myrip	360034	0.830455962	-19.61183821	grey		myosin VIIA and Rab interacting protein
Scd1	246074	0.802395299	-35.67508698	magenta	TNFa	stearoyl-Coenzyme A desaturase 1
Slc24a6	498185	1.219049861	14.66190422	black		solute carrier family 24 (sodium/potassium/calcium exchanger), member 6
Slc28a3	140944	0.683742643	-76.92920467	yellow	FGF2	solute carrier family 28 (sodium-coupled nucleoside transporter), member 3
Sult1a1	83783	0.690841501	-152.0599491	yellow	FGF2	sulfotransferase family, cytosolic, 1A, phenol-preferring, member 1
Vnn1	29142	0.762261681	-71.39348911	yellow	E2	vanin 1
**Miscellaneous & Unknown**						
Cep290	314787	0.832553925	-66.99946425	brown		centrosomal protein 290
LOC100359720	100359720	0.808065507	-30.71717541	brown	FGF2	rCG64386-like
LOC100360437	100360437	0.814579685	-22.79740618	brown		NOL1/NOP2/Sun domain family member 3-like
LOC502822	502822	1.313807192	44.12691008	brown		mCG130744-like
Pramel3	503465	0.775719249	-28.52627719	grey		preferentially expressed antigen in melanoma-like 3
RGD1306404	296733	0.826090664	-28.1292633	grey		similar to mKIAA1402 protein /// microRNA mir-671
RGD1307155	302998	0.813040278	-17.31131683	brown		similar to CG18661-PA

RGD1561507	292078	0.827948345	-45.88184289	brown		similar to hypothetical protein FLJ31606
RGD1562079	499125	0.81942471	-12.90811459	grey		RGD1562079
RGD1563556	315409	0.822780221	-72.6639627	brown		similar to mKIAA1377 protein
RGD1564791	498766	1.201625548	13.8735039	grey		similar to hypothetical protein 4930474N05
Trim2	361970	0.816329646	-21.81263945	yellow	FGF2, E2	tripartite motif-containing 2
**Proteolysis**						
Klk1l	24523	1.248576123	10.61863582	red	E2	kallikrein 1-like peptidase
Mcpt1l2	408238	1.246345345	22.31452948	brown		mast cell protease 1-like 2 /// mast cell protease 1-like 4 /// mast cell protease 1-like 3
Mmp12	117033	1.365130314	17.79691646	turquoise		matrix metallopeptidase 12
Rnf8	361815	0.825365398	-29.1619793	red		ring finger protein 8 /// FtsJ methyltransferase domain containing 2
Serpina9	299274	0.828132239	-114.1392601	pink		serine (or cysteine) peptidase inhibitor, clade A (alpha-1 antiproteinase, antitrypsin), member 9
Ubc	50522	0.795768834	-163.2679925	magenta	E2	ubiquitin C
Ubox5	296161	0.82295239	-31.31771426	brown		U-box domain containing 5
Usp26	302488	0.771722049	-98.05848112	brown		ubiquitin specific peptidase 26
**Receptors & Binding Proteins**						
Cxcr4	60628	1.236318203	35.46763926	grey		chemokine (C-X-C motif) receptor 4
Il13ra2	171060	0.771352997	-70.24582004	yellow	E2	interleukin 13 receptor, alpha 2 /// rCG23169-like
Lrp2	29216	0.770507139	-54.37286879	pink	ActivinA, AMH	low density lipoprotein-related protein 2
Notch3	56761	1.217190134	14.23854696	grey		Notch homolog 3 (Drosophila)
Olr104	293243	1.264456459	37.11842223	brown		olfactory receptor 104
Olr1063	288828	1.202484213	13.73072306	brown		olfactory receptor 1063
Olr122	293258	1.266943248	13.51430444	red		olfactory receptor 122 /// olfactory receptor Olr122-like
Olr1256	405977	1.225894824	10.21498696	brown		olfactory receptor 1256
Olr1454	287343	1.310462499	26.25776075	grey		olfactory receptor 1454
Olr1469	404976	1.301954119	18.20799584	brown		olfactory receptor Olr1469
Olr1679	405193	1.202593688	16.21998283	brown		olfactory receptor 1679
Olr252	293398	1.212870048	16.50288493	brown		olfactory receptor 252
Olr27	405258	1.214916431	11.80664657	brown		olfactory receptor 27
Olr470	295741	1.249938055	18.26955862	brown		olfactory receptor 470
Olr536	366103	1.226662652	12.53419054	brown		olfactory receptor 536
Olr630	405947	1.411098769	38.02527512	grey		olfactory receptor 630
Olr770	296023	1.264808116	11.44630049	brown		olfactory receptor 770
Olr932	366802	1.459482665	23.64329181	brown		olfactory receptor 932 /// olfactory receptor 1052
Ptch1	89830	0.824892416	-66.10082976	brown		patched homolog 1 (Drosophila)
Rtp4	360733	1.575561153	52.84805606	turquoise	ActivinA	receptor (chemosensory) transporter protein 4
Vom2r34	691767	1.231632577	18.68291388	brown		vomeronasal 2 receptor, 34 /// vomeronasal 2 receptor, 35 /// vomeronasal 2 receptor, 36 /// vomeronasal 2 receptor, 33 /// similar to putative pheromone receptor (Go-VN5)
**Signaling**						
Anxa11	290527	0.802906108	-22.54067661	grey		annexin A11
Emr1	316137	1.487111478	48.89322603	black		EGF-like module containing, mucin-like, hormone receptor-like 1
LOC499607	499607	1.331452814	24.53188833	brown		similar to GTPase activating protein testicular GAP1 /// similar to GTPase activating protein testicular GAP1
LOC691033	691033	1.328015703	10.46168849	brown		similar to GTPase activating protein testicular GAP1 /// similar to GTPase activating protein testicular GAP1
Mknk2	299618	0.811617958	-43.53256971	yellow		MAP kinase-interacting serine/threonine kinase 2
Pak3	29433	0.758556328	-293.0185949	yellow	AMH	p21 protein (Cdc42/Rac)-activated kinase 3
Phactr1	306844	0.829708089	-42.36220667	yellow		phosphatase and actin regulator 1
Pla2g2a	29692	1.306602696	219.2286103	grey		phospholipase A2, group IIA (platelets, synovial fluid)
Ptprz1	25613	1.229745183	10.55608783	brown		protein tyrosine phosphatase, receptor-type, Z polypeptide 1
RGD1308274	293347	1.20747105	10.24266813	brown		similar to very large inducible GTPase-1
S1pr3	306792	1.266346774	23.57833141	black		sphingosine-1-phosphate receptor 3
Tek	89804	0.722236378	-65.7318877	blue	FGF2	TEK tyrosine kinase, endothelial
Them4	361992	0.815136275	-25.24133166	red		thioesterase superfamily member 4
**Transcription**						
Asb13	361268	0.809211543	-37.43287274	grey		ankyrin repeat and SOCS box-containing 13
Ebf1	116543	1.21906769	17.17394938	black		early B-cell factor 1
LOC100362641	100362641	0.813205741	-44.26067188	brown		zinc finger protein 197-like
Phf11l	364393	1.250944619	15.74445299	turquoise		PHD finger protein 11-like /// PHD finger protein 11
Taf1	317256	0.818908349	-145.5816542	brown		TAF1 RNA polymerase II, TATA box binding protein (TBP)-associated factor
Znf711	302327	0.803684036	-52.92714799	blue		zinc finger protein 711
**Translation &**						

**Protein Modification**						
Hace1	361866	0.78307148	-47.28482497	brown		HECT domain and ankyrin repeat containing, E3 ubiquitin protein ligase 1
Mrpl47	294963	0.812173081	-44.47001244	brown		mitochondrial ribosomal protein L47
RGD1564552	291367	0.817793211	-51.67511039	turquoise		similar to ribosomal protein L21
Snrpf	680737	0.710696457	-59.06686268	brown		small nuclear ribonucleoprotein polypeptide F
Zrsr2	302670	0.831583693	-46.12349025	brown		zinc finger (CCCH type), RNA binding motif and serine/arginine rich 2

**F. Genes influenced by treatment with TNFa**						
**Gene Symbol**	**Entrez ID**	**Fold Change**	**Mean Diff**	**Module**	**Present in Other Lists**	**Gene Title**
**Apoptosis**						
Ecop	362374	1.204939936	18.94632291	blue		EGFR-coamplified and overexpressed protein
Pcbp4	363133	0.81353518	-40.35155787	grey	AMH	poly(rC) binding protein 4
**Cell Cycle**						
Bub1	296137	1.218445339	125.8252884	brown		budding uninhibited by benzimidazoles 1 homolog (S. cerevisiae)
Cep72	308064	1.231592605	15.40775252	brown		centrosomal protein 72kDa
Sycp1	25276	1.216531692	107.0451088	brown		synaptonemal complex protein 1
**Cytoskeleton ECM**						
Eml4	313861	1.240492133	24.33084928	grey	AMH	echinoderm microtubule associated protein like 4
Emp1	25314	1.231515345	55.16383454	blue	FGF2	epithelial membrane protein 1
Frem2	310418	0.815932405	-85.03097432	grey	FGF2	Fras1 related extracellular matrix protein 2
Kif15	353302	1.219065944	57.76934849	brown		kinesin family member 15
Lysmd2	300839	1.208382858	15.66394163	brown		LysM, putative peptidoglycan-binding, domain containing 2
Magi1	500261	0.805623129	-74.37952078	blue		membrane associated guanylate kinase, WW and PDZ domain containing 1
Ncam2	288280	0.671391808	-82.32696465	grey		neural cell adhesion molecule 2
Sorbs2	114901	0.830580008	-153.046944	turquoise	ActivinA, AMH	sorbin and SH3 domain containing 2
**Development**						
Adam19	303068	1.210484119	16.11817922	turquoise		a disintegrin and metallopeptidase domain 19 (meltrin beta) /// SRY (sex determining region Y)-box 30
Postn	361945	1.234764319	363.5586189	grey	E2	periostin, osteoblast specific factor
Prnp	24686	1.268692347	249.2584923	turquoise		prion protein
Spata22	360565	1.301138667	59.83380192	brown		spermatogenesis associated 22
Yif1b	292768	0.832029717	-51.96162232	turquoise		Yip1 interacting factor homolog B (S. cerevisiae)
**Epigenetics**						
Hist1h2bf	306969	1.265658255	43.45469688	grey	ActivinA	histone cluster 1, H2bf
Six6os1	500673	1.407047215	31.09899559	brown		Six6 opposite strand transcript 1
**EST's**						
LOC363060	363060	1.29247305	18.56412428	grey	E2	similar to RIKEN cDNA 1600029D21
---	---	0.801408611	-38.03530531	grey		---
---	---	0.748516613	-393.8638413	magenta		---
---	---	0.791757264	-136.1309425	turquoise		---
---	---	1.241918073	57.5565498	brown		---
---	---	0.810558006	-16.18957438	green		---
---	---	1.384448477	24.13406746	grey		---
---	---	1.2912951	14.86222345	brown		---
---	---	1.337233002	101.2366393	blue		---
---	---	0.807490257	-81.73053836	magenta		---
---	---	0.791495259	-42.99038392	magenta		---
---	---	1.206899441	180.9493857	brown		---
---	---	1.391823818	84.00059702	grey		---
---	---	0.735780655	-48.37537666	turquoise		---
---	---	1.266681632	30.64451646	turquoise		---
---	---	0.776210831	-536.116862	grey		---
---	---	0.823597669	-64.06180842	blue		---
---	---	1.269917863	19.96764256	green		---
---	---	0.803496593	-17.24121759	magenta		---
---	---	1.204037092	19.5989173	black		---
---	---	0.799007017	-85.92801377	grey		---
---	---	0.787924759	-33.42400613	grey		---
---	---	0.831285869	-13.23048209	green		---
---	---	1.437794095	45.28896043	magenta		---
---	---	0.824161801	-11.39744938	grey		---
---	---	0.80689709	-19.22936576	green		---
---	---	1.201389606	14.83805917	brown		---
---	---	0.832181761	-241.9836504	turquoise		---
---	---	0.817687641	-20.97538403	grey		---
---	---	1.332762865	21.32862853	grey		---
---	---	0.818439771	-89.56782743	turquoise		---
**Golgi Aparatus**						
Large	361368	0.78467593	-72.43110179	turquoise	ActivinA	like-glycosyltransferase
RGD1561381	498340	0.774044943	-86.08588391	grey		similar to microsomal glutathione S-transferase 3

**Growth Factors**						
C1qtnf7	305423	1.397853878	41.1896209	turquoise		C1q and tumor necrosis factor related protein 7
Sema4c	301346	0.771141203	-19.61077346	grey		sema domain, immunoglobulin domain (Ig), transmembrane domain (TM) and short cytoplasmic domain, (semaphorin) 4C
**Immune Response**						
Cd74	25599	0.558464567	-259.1908544	grey		Cd74 molecule, major histocompatibility complex, class II invariant chain
Ly96	448830	1.243601184	49.86262011	red		lymphocyte antigen 96
RT1-CE3	414793	0.759930913	-72.5864024	grey		RT1 class I, locus CE3
RT1-EC2	24737	0.829454762	-51.02405686	turquoise		RT1 class Ib, locus EC2 /// RT1 class I, locus CE12 /// RT1 class I, locus CE14 /// RT1 class I, locus CE5 /// RT1 class I, locus1
**Metabolism & Transport**						
Alad	25374	0.747151333	-35.28983462	turquoise		aminolevulinate, delta-, dehydratase
Aldh2	29539	0.80913747	-49.25237999	turquoise		aldehyde dehydrogenase 2 family (mitochondrial)
Alkbh7	679944	0.814898947	-27.9255572	turquoise		alkB, alkylation repair homolog 7 (E. coli)
Anpep	81641	0.756893974	-163.6289657	turquoise	ActivinA	alanyl (membrane) aminopeptidase
Clic3	296566	0.818555234	-25.36969942	blue		chloride intracellular channel 3
Etnk2	360843	0.782159224	-213.6388319	turquoise		ethanolamine kinase 2
Hsd17b2	79243	0.716506021	-103.7372969	yellow	FGF2	hydroxysteroid (17-beta) dehydrogenase 2
Msra	29447	1.209120039	14.68280623	black		methionine sulfoxide reductase A
Nqo1	24314	1.202675715	10.74188811	blue		NAD(P)H dehydrogenase, quinone 1
P4ha3	361612	0.802898921	-37.55877831	turquoise		prolyl 4-hydroxylase, alpha polypeptide III
Phyhd1	296621	0.778069027	-101.3944288	grey	FGF2	phytanoyl-CoA dioxygenase domain containing 1
Pitpnm2	304474	1.263319993	22.11391746	blue		phosphatidylinositol transfer protein, membrane-associated 2
Sblf	360202	0.824554319	-46.51873831	turquoise		stoned B-like factor
Scd1	246074	0.782770614	-40.20130987	magenta	P4	stearoyl-Coenzyme A desaturase 1
Selk	290549	1.203598108	50.48639286	brown		selenoprotein K
Ust	361450	1.227849383	21.38775951	yellow		uronyl-2-sulfotransferase
**MicroRNA & RNA Processing**						
Mir218-1	100314 248	1.336630875	15.518568	blue		microRNA mir-218-1
Mir351	100314 289	1.2229855	18.77042004	brown		microRNA mir-351
**Miscellaneous & Unknown**						
LOC685203	685203	1.245691603	50.46706128	red		hypothetical protein LOC685203
LOC686123	686123	1.228229853	99.19641531	brown		similar to leucine rich repeat and coiled-coil domain containing 1 /// leucine rich repeat and coiled-coil domain containing 1
RGD1307051	314800	0.811683632	-119.0418542	yellow		similar to hypothetical protein FLJ21963
RGD1307722	362824	0.811014033	-63.78788778	grey	CTGF, AMH	similar to hypothetical protein MGC20700
RGD1308616	362573	0.8275772	-36.62389933	turquoise	AMH	similar to KIAA0467 protein
**Proteolysis**						
Adamts17	293004	0.719065934	-86.26228775	yellow	FGF2, E2	ADAM metallopeptidase with thrombospondin type 1 motif, 17
Mmp13	171052	1.537222208	13.41563178	turquoise	ActivinA	matrix metallopeptidase 13
Mt1a	24567	0.809584684	-409.0659108	turquoise		metallothionein 1a /// hypothetical protein LOC100362769
**Receptor & Binding Proteins**						
Crabp1	25061	1.20701445	17.78017351	grey		cellular retinoic acid binding protein 1
Fzd4	64558	0.810543014	-62.84646447	turquoise		frizzled homolog 4 (Drosophila)
Igfbp5	25285	0.755730403	-694.3732003	grey	FGF2	insulin-like growth factor binding protein 5
Lphn1	65096	0.796818142	-61.88507	turquoise		latrophilin 1
Olr707	405953	0.826408785	-13.05099473	brown		olfactory receptor 707
Olr995	405265	0.830829327	-16.62495942	grey		olfactory receptor 995
Ranbp3l	294789	1.237884372	13.16751678	turquoise		RAN binding protein 3-like
Reep6	362835	0.817625169	-34.98986523	turquoise		receptor accessory protein 6
Scarb1	25073	0.747350436	-86.42232963	turquoise		scavenger receptor class B, member 1
Vom2r3	502213	1.281684267	13.87028793	yellow		vomeronasal 2 receptor, 3
**Signaling**						
Cacnb3	25297	0.753230862	-42.33840799	turquoise		calcium channel, voltage-dependent, beta 3 subunit
Chn2	84031	1.220194218	37.29015277	grey		chimerin (chimaerin) 2
Dlgap1	65040	0.830629157	-21.46367146	yellow		discs, large (Drosophila) homolog-associated protein 1
Enpp1	85496	1.399232441	216.7402185	turquoise		ectonucleotide pyrophosphatase/phosphodiesterase 1

Inpp5a	365382	0.809132967	-78.29039716	turquoise	ActivinA	inositol polyphosphate-5-phosphatase A
Itpkb	54260	0.807298625	-31.74915873	turquoise		inositol 1,4,5-trisphosphate 3-kinase B
Ndp	363443	1.239836623	11.65322219	grey		Norrie disease (pseudoglioma) (human)
Pld5	289270	1.221616852	12.47392369	grey		phospholipase D family, member 5
RGD1562646	305392	1.204049353	89.22877847	brown		similar to chromosome condensation protein G
**Transcription**						
Etv1	362733	1.265116905	27.97430926	blue		ets variant 1
Gatad2a	290669	0.809506873	-125.7769489	turquoise	ActivinA	GATA zinc finger domain containing 2A
Prrx1	266813	1.271760387	36.86848963	black		paired related homeobox 1
RGD1559530	308653	1.254918368	26.13735155	brown		similar to nuclear RNA export factor 2
Stat1	25124	0.813152243	-61.75272368	turquoise	ActivinA	signal transducer and activator of transcription 1 /// signal transducer and activator of transcription 4
Tbx2	303398	1.207581983	24.91924177	turquoise		T-box 2
Zc3h7b	315158	0.822510403	-52.97024203	turquoise		zinc finger CCCH-type containing 7B
**Translation & Protein**						
Rbm46	310548	1.2818076	70.45729769	brown	AMH	RNA binding motif protein 46
Rnase4	56759	1.329130592	17.79529198	black		ribonuclease, RNase A family 4
Snrpf	680737	1.209950617	25.17814969	brown		small nuclear ribonucleoprotein polypeptide F

G. Genes influenced by treatment with E2						
**Gene Symbol**	**Entrez ID**	**Fold Change**	**Mean Diff**	**Module**	**Present in Other Lists**	**Gene Title**
**Cell Cycle**						
Cspp1	362472	1.350198474	141.365669	green		centrosome and spindle pole associated protein 1
**Cytoskeleton ECM**						
Fbln2	282583	1.210106652	18.67893346	grey	FGF2	fibulin 2
Itm2a	317218	1.281482927	154.8851962	black		integral membrane protein 2A
LOC100360205	100360205	1.208801412	43.35587656	yellow		palladin /// similar to palladin
Mylk	288057	1.261242398	25.37491809	black		myosin light chain kinase
Myoc	81523	1.208068653	12.76777688	grey		myocilin
**Development**						
Duoxa2	499879	1.432849315	26.2600611	magenta		dual oxidase maturation factor 2
Lmcd1	494021	1.204333452	42.35422123	black		LIM and cysteine-rich domains 1
Mid1ip1	404280	0.795516972	-53.95664358	pink		MID1 interacting protein 1 (gastrulation specific G12 homolog (zebrafish))
Mllt1	301119	0.83188058	-21.91304098	grey		myeloid/lymphoid or mixed-lineage leukemia (trithorax homolog, Drosophila); translocated to, 1
Oit3	294559	1.310116749	24.05877318	blue		oncoprotein induced transcript 3
Pkhd1l1	314917	0.81799774	-26.58203579	yellow	P4	polycystic kidney and hepatic disease 1-like 1
Postn	361945	1.279070748	417.2018011	grey	TNfFa	periostin, osteoblast specific factor
Rai2	501555	0.826130903	-46.58559032	yellow		retinoic acid induced 2
Trpc3	60395	0.707198947	-55.63768674	yellow		transient receptor potential cation channel, subfamily C, member 3
**EST's**						
LOC363060	363060	1.357598173	21.6089744	grey	TNFa	similar to RIKEN cDNA 1600029D21
---	---	0.832534806	-40.05653489	magenta		---
---	---	1.235919883	10.73760871	red		---
---	---	1.202409514	16.80126286	grey		---
---	---	1.27899334	24.60007481	brown		---
---	---	1.310321798	154.5997246	green		---
---	---	0.762934067	-42.55136544	pink		---
---	---	1.308622782	71.05215425	red		---
---	---	1.359837534	22.6165171	red		---
---	---	1.229206755	19.54619076	black		---
---	---	0.7854817	-15.23702434	pink		---
---	---	1.251817232	49.27313218	red		---
---	---	1.340243994	64.64375197	red		---
---	---	1.464714009	24.73686228	green		---
---	---	1.211373448	10.23953232	pink		---
---	---	1.229231147	18.99492935	green		---
---	---	1.259790504	25.70262155	grey		---
---	---	0.829240353	-10.27283135	grey		---
---	---	1.294815002	73.956491	red		---
---	---	1.294815002	73.956491	red		---
---	---	0.827539286	-16.68202137	grey		---
---	---	1.536346291	518.568822	red		---
---	---	1.262382328	32.68001195	grey		---
---	---	1.229251629	152.5188393	green		---
---	---	1.270846133	35.80111977	grey		---
---	---	0.830231996	-64.35674241	red		---
---	---	1.27897191	33.24139388	pink		---
---	---	1.222434518	161.5650867	grey		---
---	---	0.812919502	-78.611385	grey		---
---	---	0.820263146	-13.50271763	grey		---
---	---	0.795089206	-13.63523276	pink		---
---	---	1.302156074	12.96175565	yellow		---
---	---	1.212157638	30.06329523	grey		---
---	---	0.797027087	-40.15883625	grey		---
---	---	0.729592128	-25.05561647	grey		---
---	---	0.823522536	-60.71216609	grey		---
**Golgi Aparatus**						
Man1a1	294410	0.772986063	-93.28698143	yellow		mannosidase, alpha, class 1A, member 1
**Growth Factors**						
Cxcl17	308436	1.258110092	21.42857168	red		chemokine (C-X-C motif) ligand 17
Edn2	24324	0.805546426	-15.30557739	green		endothelin 2
Igf1	24482	1.285033351	396.6734777	black		insulin-like growth factor 1
Npy	24604	1.25824878	28.21052402	grey		neuropeptide Y

Stc1	81801	0.485035316	-441.8153312	pink	FGF2, ActivinA, P4, AMH	stanniocalcin 1
**Immune Response**						
C3	24232	1.224285092	67.11603358	grey	FGF2	complement component 3
C4b	24233	1.260726184	29.41897691	grey	FGF2	complement component 4B (Chido blood group) /// complement component 4, gene 2
Ica1	81024	0.83258366	-17.32553729	grey		islet cell autoantigen 1
Igsf10	310448	1.289449672	52.56538379	black		immunoglobulin superfamily, member 10
Vsig1	315920	0.792969417	-46.10377814	grey		V-set and immunoglobulin domain containing 1
**Metabolism & Transport**						
Acsf2	619561	1.252449667	22.17959907	grey		acyl-CoA synthetase family member 2
Ak3l1	29223	0.801604365	-115.093513	yellow	FGF2	adenylate kinase 3-like 1
Atp11a	306600	0.82964215	-82.20450178	grey		ATPase, class VI, type 11A
Atp1a2	24212	1.269522077	15.36504235	blue		ATPase, Na+/K+ transporting, alpha 2 polypeptide
Atp6v0a4	296981	0.609347275	-108.9333802	pink	ActivinA, P4, AMH	ATPase, H+ transporting, lysosomal V0 subunit A4
Expi	171059	1.21994844	14.75421584	grey		extracellular proteinase inhibitor
Fmo1	25256	1.257597123	34.67908505	grey		flavin containing monooxygenase 1
Fn3krp	303755	1.31674862	45.10488687	green		fructosamine-3-kinase-related protein
Gsto1	114846	0.732605039	-143.7673952	yellow		glutathione S-transferase omega 1
Hpgd	79242	0.821350173	-15.69129365	pink	AMH	hydroxyprostaglandin dehydrogenase 15 (NAD)
Hsd3b1	360348	0.803554151	-93.1409453	yellow	FGF2	hydroxy-delta-5-steroid dehydrogenase, 3 beta- and steroid delta-isomerase 1 /// 3 beta-hydroxysteroid dehydrogenase/delta-5-delta-4 isomerase type II
Loxl1	315714	1.206751602	42.58961905	black		lysyl oxidase-like 1
Pdp2	246311	0.828310782	-33.23810055	yellow		pyruvate dehyrogenase phosphatase catalytic subunit 2
Ppap2b	192270	1.450815718	28.01170961	black		phosphatidic acid phosphatase type 2B
Slc19a2	289175	0.824406845	-23.03377465	grey		solute carrier family 19 (thiamine transporter), member 2
Slc7a6	307811	0.820179851	-70.73071611	yellow	AMH	solute carrier family 7 (cationic amino acid transporter, y+ system), member 6
Vnn1	29142	0.673662138	-110.8890932	yellow	P4	vanin 1
**MicroRNA & RNA Processing**						
Mir301a	100314217	0.782791171	-14.61698127	grey		microRNA mir-301a
Trnt1	312616	0.805080241	-71.23593454	grey		tRNA nucleotidyl transferase, CCA-adding, 1
**Miscellaneous & Unknown**						
Rcan3	362627	1.264566122	24.08601919	grey		RCAN family member 3
Tmem82	298605	1.288401784	27.8317427	red		transmembrane protein 82
Trim2	361970	0.815988428	-21.86230073	yellow	FGF2, P4	tripartite motif-containing 2
**Proteolysis**						
Adamts17	293004	0.805705281	-53.24392548	yellow	FGF2, TNFa	ADAM metallopeptidase with thrombospondin type 1 motif, 17
Klk1l	24523	1.234101565	10.11760581	red	P4	kallikrein 1-like peptidase
Serpine1	24617	1.587236981	40.4324885	turquoise		serine (or cysteine) peptidase inhibitor, clade E, member 1
Serpinf1	287526	1.277471785	49.04634266	brown		serine (or cysteine) peptidase inhibitor, clade F, member 1
Ubc	50522	0.822056036	-137.7043949	magenta	P4	ubiquitin C
**Receptors & Binding Proteins**						
Adora2b	29316	1.21417325	12.75322512	grey	AMH	adenosine A2B receptor
Esr2	25149	1.227595086	19.66139007	black		estrogen receptor 2 (ER beta)
Il13ra2	171060	0.688037957	-107.4477938	yellow	P4	interleukin 13 receptor, alpha 2 /// rCG23169-like
S100g	24249	0.551829229	-106.9406146	blue	AMH	S100 calcium binding protein G
Sorcs3	294043	0.689439973	-45.17701966	grey	AMH	sortilin-related VPS10 domain containing receptor 3
Tfrc	64678	0.738022059	-261.0183253	yellow		transferrin receptor
**Signaling**						
Abcb1b	24646	0.824939332	-40.79206807	grey		ATP-binding cassette, sub-family B (MDR/TAP), member 1B /// ATP-binding cassette, sub-family B (MDR/TAP), member 1A
Abl2	304883	1.20765047	21.81187531	grey		v-abl Abelson murine leukemia viral oncogene homolog 2 (arg, Abelson-related gene)
Arap2	305367	0.783812217	-155.3935539	red		ArfGAP with RhoGAP domain, ankyrin repeat and PH domain 2

Arrb1	25387	1.246274555	26.3371539	black		arrestin, beta 1
Arrb2	25388	1.237501733	19.76092366	grey		arrestin, beta 2
Ehd4	192204	0.828230196	-58.75719251	grey		EH-domain containing 4
Entpd2	64467	1.247467713	25.86448321	black		ectonucleoside triphosphate diphosphohydrolase 2
Itga2	170921	1.215828199	33.43220484	grey		integrin, alpha 2
Itgb8	362800	0.832736204	-30.56019757	red		integrin, beta 8
Mobkl2b	366352	1.323661673	33.00651935	black		MOB1, Mps One Binder kinase activator-like 2B (yeast)
Pde3a	50678	1.277118939	32.36965452	black		phosphodiesterase 3A, cGMP inhibited
Plce1	114633	1.215267034	14.73203802	blue		phospholipase C, epsilon 1
Plekhh3	360634	0.829763421	-17.77716298	blue		pleckstrin homology domain containing, family H (with MyTH4 domain) member 3
Rasd1	64455	0.774294814	-51.36421016	yellow		RAS, dexamethasone-induced 1
Rgnef	361882	1.213433325	24.63654575	black		Rho-guanine nucleotide exchange factor
Sparcl1	25434	1.40351637	89.85653602	black		SPARC-like 1 (hevin)
Trhde	366894	0.827145937	-12.41970623	yellow		thyrotropin-releasing hormone degrading enzyme
**Transcription**						
Id1	25261	0.816086225	-77.3413855	magenta		inhibitor of DNA binding 1
Ncoa2	83724	1.448933877	91.01461675	grey		nuclear receptor coactivator 2
Nupr1	113900	1.350614063	195.6470591	blue		nuclear protein, transcriptional regulator, 1
Zfp185	689949	0.807491237	-17.24701517	yellow	AMH	zinc finger protein 185
**Translation & Protein Modification**						
Egln1	308913	0.799449598	-19.24287967	brown		EGL nine homolog 1 (C. elegans)
Gemin8	363462	1.260241211	79.68462327	green		gem (nuclear organelle) associated protein 8
Tgm2	56083	1.376775018	65.5787165	grey		transglutaminase 2, C polypeptide
