# Supplementary material for: Gene bionetworks that regulate ovarian primordial follicle assembly
Source: BMC Genomics. 2013 Jul 23;14:496. doi: 10.1186/1471-2164-14-496 (PMC3726361; doi:10.1186/1471-2164-14-496)
Supplement: Additional file 3: Table S2 — Treatment and module differentially expressed genes correlated to cellular pathways and processes. [file 1471-2164-14-496-S3.pdf]

Supplemental Table S2

KEGG ID[a]	Pathway name	Total # Genes[b]	Treatments[c]							Modules[d]									Hyper-[e] geometric Probability	Fisher's Two[f] Sided P-value
			AMH	CTGF	E2	FGF2	Activin A	P4	TNFa	Turquoise	Blue	Brown	Yellow	Green	Red	Black	Pink	Magenta		
rno01100	Metabolic pathways	59	9		6	11	23	9	10	23	6	9	8	2		1	3		0.01624	0.1113
rno05200	Pathways in cancer	19	3		3	7	3	3	2	3	5	5	1			1			0.02052	0.078
rno04740	Olfactory transduction	19	2	3	1		2	11	1			**12**			1			1	0.0177	0.09285
rno04010	MAPK signaling pathway	15			2	7	2	3	1	3	6		2			2			0.0502	0.209
rno04510	Focal adhesion	15	1		4	5	4	3		2	6	1	2		1	2			0.005	0.013
rno04062	Chemokine signaling pathway	15	3		2	6	4	1	1	3	4		2			1		1	0.003	0.006
rno04144	Endocytosis	15			5	3	3	2	2	2	4	1	1		1	1			0.021	0.059
rno00980	Metabolism of xenobiotics by cytochrome P450	14		1	1	3	3	6		5	4	1	1						8.02E-06	1.00E-05
rno04145	Phagosome	13	1		4	3	4	2	3	5	2		1				1		0.017	0.054
rno04020	Calcium signaling pathway	12	5		3	4	1	1	1	1	5		1	1		1		1	0.025	0.075
rno04514	Cell adhesion molecules	12	1		1	4	2	1	3	3	3			1	1		1		0.013	0.035
rno04360	Axon guidance	11	1			6	3	3	1	2	6		1						0.008	0.019
rno04810	Regulation of actin cytoskeleton	11	2		3	4	2	2		2	3		2		1	1			0.082	0.369
rno00480	Glutathione metabolism	11		1	1	3	3	3	1	5	3		1						6.72E-06	7.86E-06
rno00230	Purine metabolism	11	3		3	3	2		1	2	2	2	2			2			0.038	0.1101
rno03010	Ribosome	11					11			10		1							9.00E-03	2.10E-02
rno04060	Cytokine-cytokine receptor interaction	11				6	3	2		3	5					1			0.113	0.619
rno04080	Neuroactive ligand-receptor interaction	10	2		1	6	3	1		1	4	1	1			1			0.084	0.476
rno04976	Bile secretion	10	2		2	4	1	1	1	2	5								0.00044	0.00057
rno04380	Osteoclast differentiation	9				5	3	1	1	3	4		1			1			0.033	0.065
rno04640	Hematopoietic cell lineage	9	3	1	2	2	1	1	1	1	2	1	3			1			0.003	0.004
rno04110	Cell cycle	8	1			5	1		1		3	**4**	1						0.0603	0.158
rno04512	ECM-receptor interaction	7	1		2	2	2	1		2	2			1	1				0.0202	0.0304
rno04612	Antigen processing and presentation	7				1		3	3	1	1								0.041	0.094
rno04972	Pancreatic secretion	7			1	3	2	1		1	5								0.065	0.197
rno04350	TGF-beta signaling pathway	7	1		1	3	2			3	1		1					1	0.034	0.085
rno04666	Fc gamma R-mediated phagocytosis	6			1	4	1			1	4					1			0.088	0.187
rno04961	Endocrine and other factor-regulated calcium reabsorption	6			2	4		1			**5**				1				0.011	0.014
rno04115	p53 signaling pathway	6	1		2	2	1			1	2	2				1			0.041	0.065
rno04916	Melanogenesis	6	1			4			1	1	2							1	0.097	0.286
rno04610	Complement and coagulation cascades	6	1		3	4				1	1		1						3.80E-02	0.058
rno04971	Gastric acid secretion	6	1		2	3					4					1		1	0.043	0.068

rno04978	Mineral absorption	6	1		2	2	1		1	2	4								0.0075	0.0097
rno04724	Glutamatergic synapse	6	2			3		1	1		2		2						0.153	0.648
rno04726	Serotonergic synapse	6	3			4	1	1			1		2						0.153	0.648
rno04310	Wnt signaling pathway	6				4	1		1	1	2				1				0.164	1
rno04142	Lysosome	6	1		1	3	3	1		2	3						1		0.149	0.641
rno04260	Cardiac muscle contraction	6			1	3		1	1	1	4								0.067	0.149
rno05214	Glioma	6	1		1	3	1				3	1				1		1	0.024	0.035
rno04620	Toll-like receptor signaling pathway	6				2	2	1	2	2	1		1		1	1			0.081	0.173
rno04670	Leukocyte transendothelial migration	6				2	3	1		3	1						1		0.135	0.469
rno04728	Dopaminergic synapse	6	3	1	1	2					1		1				1	1	0.155	0.652
rno04910	Insulin signaling pathway	6	2		1	2		1			2		1	1		1		1	0.158	0.658
rno04270	Vascular smooth muscle contraction	6	2		2	2		1			2					1		1	0.152	0.646
rno04530	Tight junction	6		1		1	2	1	1	1	2						2		0.157	0.656
rno04914	Progesterone-mediated oocyte maturation	5	1		2	1			1		1	2				2			0.126	0.391
rno04912	GnRH signaling pathway	5	1			3		1			2		1					1	0.155	0.597
rno04727	GABAergic synapse	5	2			4					3		1						0.143	0.416
rno04630	Jak-STAT signaling pathway	5			1	3	1	1	1	1	2		1						0.172	1
rno03320	PPAR signaling pathway	5	1		1		1	3	1	1		1						2	0.107	0.238
rno04974	Protein digestion and absorption	5	1		1	2	1				**4**								0.105	0.233
rno04920	Adipocytokine signaling pathway	5	1		1	2		1		1	2								0.088	0.206
rno04070	Phosphatidylinositol signaling system	5	1		1	1	1		2	2	2							1	0.113	0.249
rno04114	Oocyte meiosis	5	2		1	1			1		1	2				1		1	0.176	0.807
rno04970	Salivary secretion	5	1		1	3					**4**							1	0.105	0.233
rno04540	Gap junction	5	1			3	3			2	2		1						0.134	0.401
rno00532	Glycosaminoglycan biosynthesis	4				1		2	1		1		1							
rno04725	Cholinergic synapse	4	2			3					2		1							
rno04120	Ubiquitin mediated proteolysis	4				1	1	2				**3**	1							
rno04012	ErbB signaling pathway	4	1		1	2		1			2		1							
rno00620	Pyruvate metabolism	4	1			1	1		1	1		2		1						
rno04623	Cytosolic DNA-sensing pathway	4	1				2	1		2						1				
rno00590	Arachidonic acid metabolism	4					1	3		1		1								
rno00010	Glycolysis / Gluconeogenesis	4				1	2		1	3		1								
rno00140	Steroid hormone biosynthesis	4			1	3		1	1		2		2							
rno04723	Retrograde endocannabinoid signaling	4	2			3					2		1							
rno00190	Oxidative phosphorylation	4	1		1	1	3	1		2	1						1			
rno04210	Apoptosis	4				2		2			2		1			1				
rno04744	Phototransduction	4	1		2	1						1				1		1		
rno03013	RNA transport	4			2		1		1			1		1	1					
rno04141	Protein processing in endo ret	4	1		1		2	1		1			1				1			
rno04960	Aldosterone-	4			2	2					3					1				

	regulated sodium reabsorption																			
rno04622	RIG-I-like receptor signaling pathway	3					3			3										
rno00240	Pyrimidine metabolism	3	1				2			1			1							
rno00533	Glycosaminoglycan biosynthesis - keratan sulfate	3				1	1	1				1	1				1			
rno04977	Vitamin digestion and absorption	3			1	1			1	1	1									
rno01040	Biosynthesis of unsaturated fatty acids	3				2		1	1		2							1		
rno00510	N-Glycan biosynthesis	3	1		1	1						1	2							
rno04964	Proximal tubule bicarbonate reclamation	3			1	1	1			1	2									
rno03410	Base excision repair	3	3							1			1							
rno00561	Glycerolipid metabolism	3	1		1				1	1				1		1				
rno04330	Notch signaling pathway	3	1			2		1			1		1							
rno04340	Hedgehog signaling pathway	3	1				2	2		1		1					1			
rno00562	Inositol phosphate metabolism	3			1		1		2	2	1									
rno04973	Carbohydrate digestion and absorption	3			1	2					**3**									
rno04621	NOD-like receptor signaling pathway	3	1					2					1			1		1		
rno00564	Glycerophospholipid metabolism	3			1			1	1	1						1				
rno00565	Ether lipid metabolism	3			1		1	1		1						1				
rno04975	Fat digestion and absorption	3			1			1	1	1						1				
rno04370	VEGF signaling pathway	3				1	1	1		1	1									
rno00330	Arginine and proline metabolism	3	1						2	2		1								
rno04150	mTOR signaling pathway	3			1	1		1				1				1				
rno04722	Neurotrophin signaling pathway	3	1			2					2							1		

[a]Identification code for the indicated physiological pathway, from KEGG (Kyoto Encyclopedia for Genes and Genome).
[b]Total number of genes in common with the indicated pathway, using the complete set of 1081 differentially expressed genes. For this table one gene = one unique Entrez ID.
[c]Number of genes differentially expressed with each treatment that are in common with the individual pathway. The same gene may be differentially expressed with more than one treatment.
[d]Number of differentially expressed in each module that are in common with the indicated pathway. Those genes not assigned to a specific module are not indicated.
[e]p-value from hypergeometric probability calculation that the total number of genes would be found in the listed KEGG pathway by chance.
[f]p-value from Fischer's Exact test that the listed total number of differentially expressed genes, or fewer, would be in common with the indicated pathway by chance.
